# Cytosporin Derivatives from Arctic-Derived Fungus *Eutypella* sp. D-1 via the OSMAC Approach

**DOI:** 10.3390/md21070382

**Published:** 2023-06-28

**Authors:** Hao-Bing Yu, Zhe Ning, Bo Hu, Yu-Ping Zhu, Xiao-Ling Lu, Ying He, Bing-Hua Jiao, Xiao-Yu Liu

**Affiliations:** 1 Naval Medical Center of PLA, Department of Marine Biomedicine and Polar Medicine, Naval Medical University, Shanghai 200433, China; yuhaobing1986@126.com (H.-B.Y.); ningzhe95@163.com (Z.N.); hb8601@163.com (B.H.); yinghe_hys@163.com (Y.H.); 2 Basic Medical Experimental Teaching Center, College of Basic Medical Sciences, Naval Medical University, Shanghai 200433, China; zhuyuping72@hotmail.com; 3 Department of Biochemistry and Molecular Biology, College of Basic Medical Sciences, Naval Medical University, Shanghai 200433, China; luxiaoling80@126.com (X.-L.L.); jiaobh@live.cn (B.-H.J.)

**Keywords:** cytosporin, arctic fungus, *Eutypella* sp., immunosuppressive activity

## Abstract

A chemical investigation of the Arctic-derived fungus *Eutypella* sp. D-1 based on the OSMAC (one strain many compounds) approach resulted in the isolation of five cytosporin polyketides (compounds **1**–**3** and **11**–**12**) from rice medium and eight cytosporins (compounds **2** and **4**–**11**) from solid defined medium. The structures of the seven new compounds, eutypelleudesmane A (**1**), cytosporin Y (**2**), cytosporin Z (**3**), cytosporin Y_1_ (**4**), cytosporin Y_2_ (**5**), cytosporin Y_3_ (**6**), and cytosporin E_1_ (**7**), were elucidated by analyzing their detailed spectroscopic data. Structurally, cytosporin Y_1_ (**4**) may be a key intermediate in the biosynthesis of the isolated cytosporins, rather than an end product. Compound **1** contained a unique skeleton formed by the ester linkage of two moieties, cytosporin F (**12**) and the eudesmane-type sesquiterpene dihydroalanto glycol. Additionally, the occurrence of cyclic carbonate moieties in compounds **6** and **7** was found to be rare in nature. The antibacterial, immunosuppressive, and cytotoxic activities of all compounds derived from *Eutypella* sp. D-1 were evaluated. Unfortunately, only compounds **3**, **6**, **8**, and **10**–**11** displayed immunosuppressive activity, with inhibitory rates of 62.9%, 59.5%, 67.8%, 55.8%, and 68.7%, respectively, at a concentration of 5 μg/mL.

## 1. Introduction

Fungi have proven to be a valuable source of new secondary metabolites with a wide spectrum of biological activities [1]. Natural products from Polar fungi remain the non-negligible sources of pharmacologically active compounds [1]. Cytosporins are a family of hexahydrobenzopyran metabolites derived from fungi with a distinct heptene side chain residue [2]. Initially isolated from endophytic *Cytospora* sp. in 1996, cytosporins were recognized as inhibitors of angiotensin II binding inhibitors [2]. To date, nearly 30 natural cytosporins of this structural class have been predominantly isolated from four genera of fungi: *Cytospora* sp. [2], *Pestalotiopsis* sp. [3], *Eutypella* sp. [4,5], and *Pseudopestalotiopsis* sp. [6]. The cytosporin family exhibits diverse bioactive effects, including cytotoxic, antibacterial, and antagonistic activity [2,4]. Besides cytosporins, *Eutypella* species have been extensively investigated as a rich source of various bioactive compounds, pimarane diterpenes, γ-lactones, benzopyrans, *ent*-eudesmanes, cytochalasins, and dipeptides, which display a spectrum of bioactivities [7,8,9].

**Figure 1:**
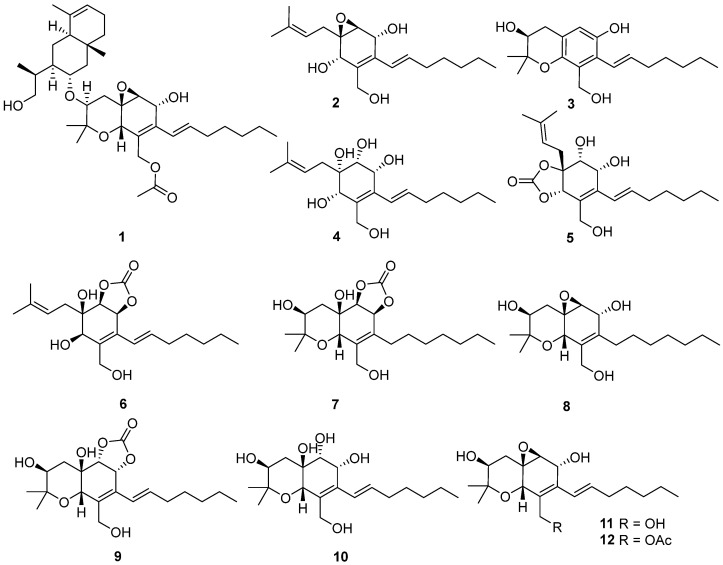
Structures of the isolated compounds **1**–**12**.

The OSMAC approach has emerged as a powerful tool in the field of natural product biodiscovery, stimulating the production of a wider range of new metabolites [10]. During our exploration of structurally diverse bioactive natural products from polar fungi, we discovered a series of terpenoids with unique skeleton characteristics from the talented Arctic fungus strain *Eutypella* sp. D-1 [5,7,9]. This strain has proven to be a prolific source of metabolites with diverse biological activities [7,9]. To enhance the chemical diversity of *Eutypella* sp. D-1, we employed the one strain many compounds (OSMAC) strategy, utilizing different culture conditions. Through high-performance liquid chromatography (HPLC) analysis, some structural analogs during fermentation on two distinct media, solid rice medium and defined solid medium, were dramatically discovered. Subsequent chemical investigation led to the isolation of 12 cytosporin derivatives, including seven new cytosporins—eutypelleudesmane A (**1**), cytosporin Y (**2**), cytosporin Z (**3**), cytosporin Y_1_ (**4**), cytosporin Y_2_ (**5**), cytosporin Y_3_ (**6**), and cytosporin E_1_ (**7**)—together with five known biogenetic-related analogs—cytosporin X (**8**), cytosporin E (**9**), cytosporin L (**10**), and cytosporins D and F (**11**–**12**) (Figure 1). Herein, we present the detailed purification, structure elucidation, and bioactive evaluation of these compounds.

## 2. Results

Eutypelleudesmane A (**1**) was isolated as a light-brown oil. The molecular formula was determined as C_36_H_56_O_7_ from HRESIMS and NMR data (Table 1), indicating the presence of nine degrees of unsaturation. The IR spectra confirmed the presence of hydroxy (3357 cm^−1^) and carbonyl (1741 cm^−1^) groups [3,4,5]. Additionally, the ^13^C NMR analysis revealed one ester carbonyl signal (δ_C_ 171.0) and six double-bond carbon signals (δ_C_ 121.2, 124.7, 125.1, 134.1, 135.6, and 136.4), accounting for four degrees of unsaturation. The remaining five degrees of unsaturation were attributed to the pentacyclic ring structure present in the molecule.

**Figure 2:**
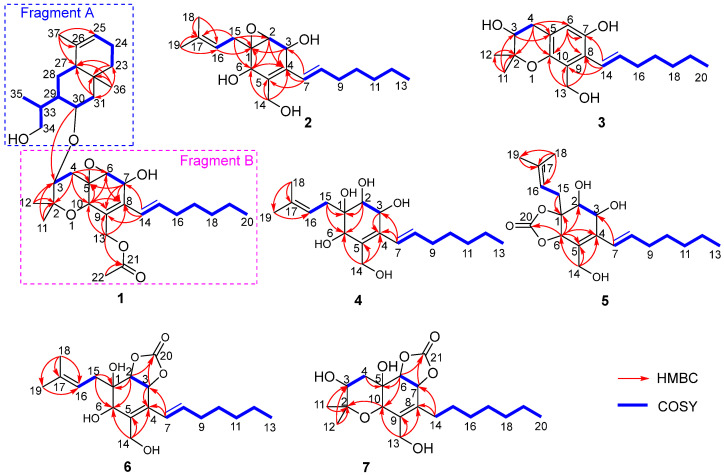
Key COSY and HMBC correlations of **1**–**7**.

Upon comparing the 1D NMR data of compound **1** and cytosporin F (**12**), it was observed that one set of signals was similar to compound **12**, while the remaining signals resembled a derivative of eudesmane-type sesquiterpene, dihydroalanto glycol. By utilizing 2D NMR correlations (Figure 2), these two structural fragments, labeled as A and B, were deduced. The COSY spectrum revealed the presence of seven continuous spin systems: (a) C-3−C-4, (b) C-6−C-7, (c) C-14–C-15–C-16–C-17–C-18–C-19–C-20, (d) C-23–C-24–C-25, (e) C-27–C-28–C-29–C-30–C-31, (f) C-29–C-33–C-34, and (g) C-33–C-35 (Figure 2). Fragment A, comprising C-2 to C-22, exhibited similarity to compound **12** based on a comparison of their 1D NMR spectra. HMBC correlations from H-4α to C-2, C-5, C-6, and C-10; from H-6 to C-8; from H-7 to C-5, C-8, and C-9; from H-10 to C-5, C-6, C-8, and C-9; from H_3_-11 and H_3_-12 to C-2 and C-3; and from H_2_-13 to C-8, C-9, and C-10 were detected. These correlations, along with the chemical shift of C-2 (δ_C_ 76.6) and C-10 (δ_C_ 67.5), indicated the formation of two six-membered rings by connecting C-5 (δ_C_ 55.7) with C-10 and C-2 with C-10 via an *O*-atom, as well as the location of the two methyl groups CH_3_-11 and CH_3_-12 both at C-2 and one methylene group CH_2_-13 at C-9. The presence of an oxirane resulting from the conjugation of C-5 and C-6 via *O*-atom was supported by the downfield shift of C-5 and C-6 (δ_C_ 59.7) [3,4]. Furthermore, the direct linkage between C-8 and C-14 was established by HMBC correlations from H-14 to C-7, C-8, and C-9. An additional acetyl group was identified to be connected to C-13 based on the HMBC correlations from H-13 and H-22 to C-21 and the chemical shift of C-13 (δ_C_ 61.5). Fragment B, spanning from C-23 to C-37, exhibited characteristics of a eudesmane-type sesquiterpene moiety, as deduced from the analysis of the remaining ^1^H and ^13^C NMR data. HMBC correlations from H-31α and H-31β to C-27 and C-32; from H_2_-23 to C-27 and C-32; and from H_3_-36 to C-23, C-27, C-31, and C-32 confirmed the presence of a linkage of C-23, C-27, and C-31 via the quaternary carbon C-32, and placed the methyl group CH_3_-36 at C-32 as well. The methyl group CH_3_-37 was demonstrated to be connected to C-25 and C-27 via C-26 by the HMBC correlations from H_3_-37 to C-25, C-26, and C-27. The linkage of fragments A with B through C-3 (δ_C_ 73.8) and C-30 (δ_C_ 66.7) via an *O*-atom was supported by the downfield resonance of C-3 and C-30, along with HMBC correlations from H-30 to C-3. Additionally, the connection of two hydroxyl groups with a downfield carbon shift at C-7 (δ_C_ 64.6) and C-34 (δ_C_ 67.5) were determined to satisfy the molecular formula. Consequently, the planar structure of **1** was established as depicted.

**Figure 3:**
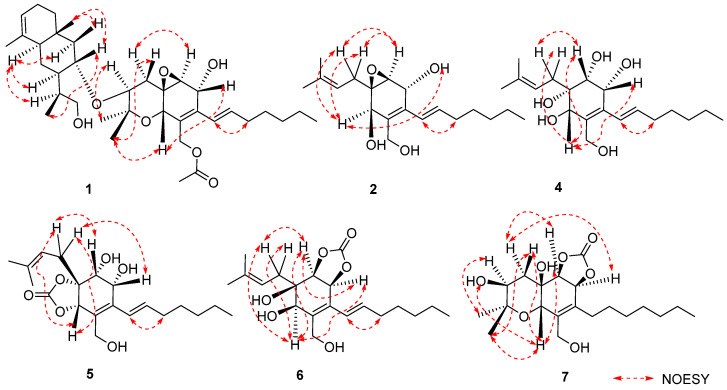
Key NOESY correlations of **1**, **2**, and **4**–**7**.

**Figure 4:**
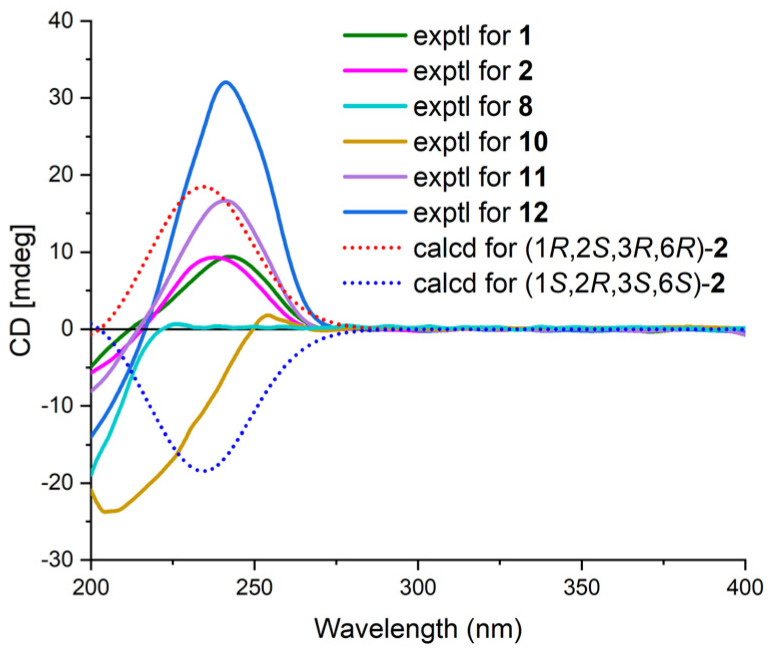
ECD spectra of **1**, **8**, and **10**–**12** and calculated and experimental ECD spectra of **2**.

The relative configuration of **1** was established by analyzing coupling constants and NOESY experiments [9]. The *trans* configuration of the conjugated C-14/C-15 double bond was inferred based on the large coupling constant (16.0 Hz) and the NOESY correlations of H-14/H_2_-16. The observed similarity in the NMR chemical shift values and NOESY correlations of H-7/H-10, H-10/H_3_-12, H_3_-12/H-4β, H-4α/H-6, and H-3/H_3_-11 indicated that the relative configurations of fragment A in **1** were identical to those of **12** [3,4,5]. Additional NOESY correlations of H-30/H_3_-35, H-30/H-36β, and H-31β/H-36β and those of H-27/H-29, H-27/H-31α, and H-27/H-33 indicated the β-orientation and α-orientation of these protons in fragment B, respectively (Figure 3). Furthermore, the absence of a NOESY correlation between H-3 and H-30 supported the *trans* relationship between these two protons [5]. Thus, the relative structure of **1** was determined. Furthermore, the characteristic positive Cotton effect at 242 nm in the CD spectrum of **1** was virtually identical to that of cytosporins D and F (**11**–**12**) (Figure 4) [5], which suggested the absolute configuration of **1** was assigned as 3*S*,5*R*,6*S*,7*R*,10*S*,27*S*,29*S*,30*R*,32*S*,33*R*.

Cytosporin X (**2**) was obtained as a light-brown oil and determined to have a molecular formula of C_19_H_30_O_4_ based on HRESIMS and NMR data, corresponding to an unsaturation index of 5. The presence of hydroxy functionality was indicated by IR absorption bands at 3359 cm^−1^. The ^13^C NMR (Table 2) and DEPT spectra revealed the presence of 19 carbons, including six double-bond carbon signals (δ_C_ 117.3, 124.6, 131.4, 131.6, 135.4, and 135.9) and five oxygenated carbon signals (δ_C_ 57.4, 59.3, 62.2, 64.3, and 69.5). The COSY spectrum of **2** showed three distinct spin systems: C-2/C-3, C-7/C-8/C-9/C-10/C-11/C-12/C-13, and C-15/C-16 (Figure 2). HMBC correlations from H-2 to C-4 and C-6; from H-3 to C-1 and C-4; from H-6 to C-1, C-2, and C-4; and from H_2_-14 to C-4, C-5, and C-6, along with the comparison of the chemical shifts of C-1 (δ_C_ 59.3) and C-2 (δ_C_ 57.5) to those of cytosporins D and F [3,4], determined the oxirane-fused cyclohexene moiety with one methylene group (CH_2_-14) attached at C-5. The isoamylene group was connected to C-1 based on the HMBC correlations from H_2_-15 to C-1, C-2, and C-6, as well as from H_3_-18 and H_3_-19 to C-16 and C-17. Further HMBC correlations from H-7 to C-3, C-4, and C-5 established the connectivity of C-4 and C-7. With this assignment secured, each of the three oxygenated carbon at C-3 (δ_C_ 64.3), C-6 (δ_C_ 69.5), and C-14 (δ_C_ 62.2) had to be substituted with a hydroxy group to satisfy the molecular formula. The relative stereocenter of 2 was determined from NOESY correlations and coupling constants in comparison with those of **11** and **12** [3,4]. The conjugated C-7/C-8 double bond was assigned as *trans* upon its large coupling constant (16.0 Hz). The NOESY correlations of H-2/H_2_-15 and H-6/H_2_-15 in CDCl_3_ and 3-OH/H-6 in DMSO-*d*_6_ (Figure S16), combined with the similarity between the calculated and the experimental ECD spectra, confirmed the absolute configurations of **2** as 1*R*,2*S*,3*R*,6*R* (Figure 4).

Cytosporin Y (**3**) exhibited a negative HRESIMS with a pseudomolecular ion at *m/z* 319.1912 [M − H]^−^, consistent with the molecular formula of C_19_H_28_O_4_. The similarity of the ^1^H and ^13^C NMR data between **3** and **11** indicated that compound **3** was the derivative of **11**. The presence of a pentasubstituted benzene moiety (δ_H_ 6.61 (1H, s); δ_C_ 115.1 (CH), 118.8 (C), 123.6 (CH), 126.8 (C), 144.8 (C), and 146.7 (C)) instead of the oxirane-fused cyclohexene moiety in **11** was suggested by the ^1^H and ^13^C NMR spectra. This was further confirmed by the further HMBC correlations from H-4β to C-5, C-6, and C-10; from H-6 to C-7, C-8, and C-10; from H_2_-13 to C-8, C-9, and C-10; and from H-14 to C-7, C-8, and C-9 (Figure 2). Additionally, one hydroxy group was attached to C-7, as evidenced by its chemical shift (δ_C_ 146.7) and the molecular formula. The conjugated C-14/C-15 double bond in **3** was assigned as *trans* based on the similar ^1^H NMR chemical shift values and coupling constants (16.5 Hz) observed in **3** and **2**. To determine the absolute configuration at C-3 in compound **3**, the specific rotation (${\left[\alpha \right]}_{D}^{25}$ +12.3, MeOH, *c* 0.1 and ${\left[\alpha \right]}_{D}^{25}$ +1.9, CDCl_3_, *c* 0.1) was measured. The configuration of C-3 could be assigned as *S* by comparison to the literature data for synthetic (*R*)-2,2-dimethylchromane-3,7-diol (${\left[\alpha \right]}_{D}^{20}$ −1.2, MeOH, *c* 0.03) [11] and (*S*)-2,2-dimethylchromane-3,7-diol (${\left[\alpha \right]}_{D}^{23}$ +11.5, CHCl_3_, *c* 1.0) [12] (differences in measured versus literature values likely stem from the different concentration and solvent). 

**Figure 5:**
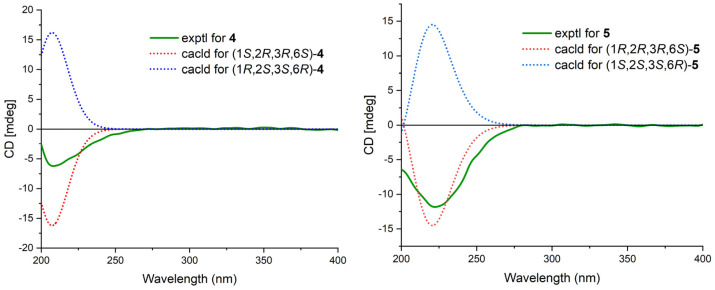
Calculated and experimental ECD spectra of **4** and **5**.

Cytosporin Y1 (**4**), in the form of a light-yellow oil, had a molecular formula of C_19_H_32_O_5_ based on HRESIMS (*m/z* 363.2139 [M + Na]^+^), which is larger than that of cytosporin Y (**2**) by 18 amu. The NMR data of **4** (Table 3) were nearly identical to those of **2**, indicating the same carbon skeleton. Considering the degrees of unsaturation of **4**, the observed downfield shift of one quaternary carbon (δ_C_ 59.3) and one methine (δ_H_/δ_C_ 3.29/57.5) in **2** to δ_C_ 74.3 and δ_H_/δ_C_ 3.76/75.0 in **4**, respectively, suggested that **4** was the oxirane ring-opening product of **2**. This hypothesis was further supported by further COSY and key HMBC correlations, as shown in Figure 2. The *E*-geometry of the Δ^7,8^ double bond was deduced from a NOESY correlation between H-7 and H_2_-9, as well as the coupling constants (16.5 Hz). Additional NOESY correlations of H-2/H_2_-15, H-2/H-6, H-3/H-6, and H-6/H_2_-15 indicated the same orientation of these protons. Furthermore, the comparison of the calculated and the experimental ECD spectra confirmed the absolute configurations of **4** as 1*S*,2*R*,3*R*,6*S* (Figure 5).

Cytosporin Y2 (**5**) was obtained as a light-yellow oil. Extensive NMR analyses and HRESIMS data (*m/z* 389.1928 [M + Na]^+^) led to the determination of its molecular formula as C_20_H_30_O_6_. The overall NMR data of **5** indicated a structure similar to **4**, with the notable difference of an additional quaternary carbon. This carbon was identified as a carbonate moiety based on the strong IR absorption at 1647 cm^−1^ and the diagnostic ^13^C NMR signal at δ_C_ 154.8 [4]. Another significant difference was observed for C-1 and C-2, resonating at δ_C_ 74.3 and 75.0 in compound **4**, whereas in compound **5**, these signals resonated at δ_C_ 84.4 and 71.3, respectively (Table 3). This observation, along with the key HMBC correlations from H-2 to C-20, led to the linkage of the carbonyl to both oxygen atoms at C-1 and C-2 to form a cyclic carbonate moiety. The relative configurations of **5** were determined via a detailed analysis of the NOESY correlations of H-2/H-15a, H-3/H-15b, H-6/H-15a, H-6/H-15b, and H-7/H_2_-9, as well as the coupling constants (16.5 Hz) of H-7/H_2_-9. Furthermore, the calculated ECD spectrum of **5** exhibited a close resemblance to the experimental one, confirming the absolute configuration as 1*R*,2*R*,3*R*,6*S* (Figure 5).

**Figure 6:**
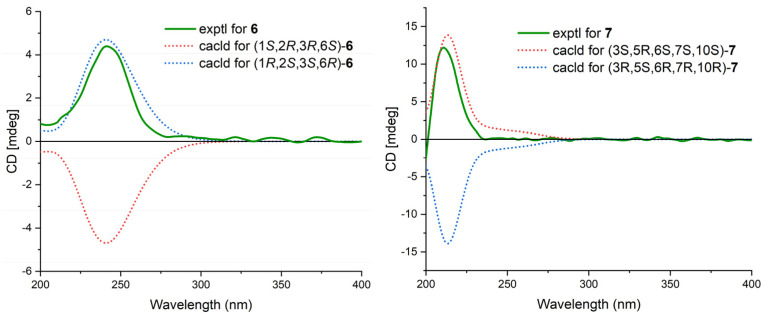
Calculated and experimental ECD spectra of **6** and **7**.

Cytosporin Y3 (**6**) was isolated as a light-yellow oil. Its molecular formula was determined to be C_20_H_30_O_6_, the same as that of **5**, based on HRESIMS data. A comparison of the IR, UV, and NMR data (Table 4) of **6** with those of **5** suggested that **6** was an isomer of **5**. Further analysis of the ^13^C NMR chemical shift of C-2 (δ_C_ 79.2) and C-3 (δ_C_ 75.2), along with the unambiguous HMBC correlations from H-2 and H-3 to C-20 of **2**, revealed that the cyclic carbonate moiety was fused with C-2-C-3 in **6**. The relative configuration and *E*-geometry of the Δ^7,8^ double bond in **6** were determined from the NOESY correlations of H-2/H-3, H-2/H-6, H-3/H-6, H-2/H-15b, H-6/H-15a, H-6/H-15b, and H-7/H_2_-9. The absolute configuration of **6** was subsequently determined to be 1*R*,2*S*,3*S*,6*R* based on the opposite CD spectra (Figure 6) and a comparison of the specific rotation (${\left[\alpha \right]}_{D}^{20}$ +37.8, MeOH, *c* 0.1) with that of **4** (${\left[\alpha \right]}_{D}^{20}$ −15.8, MeOH, *c* 0.1) and **5** (${\left[\alpha \right]}_{D}^{20}$ −60.1, MeOH, *c* 0.1) (Figure 6).

Cytosporin E1 (**7**) was also purified as a light-yellow oil and exhibited a HRESIMS ion peak at *m/z* 407.2034 [M + Na]^+^, consistent with the molecular formula C_20_H_32_O_7_ with five degrees of unsaturation. The ^1^H and ^13^C NMR data of **7** (Table 4) closely resembled those of the known compound cytosporin E (**9**), except for two additional methylenes (δ_C_/δ_H_ 31.0/2.26 and 30.0/1.49) in **7** and the absence of two olefinic methines (δ_C_/δ_H_ 123.5/6.39 and 137.1/6.03) in **9**. These observations indicated that C-8 in **7** was substituted by a heptane subunit instead of the 1-heptene part in **9**, which was also confirmed by COSY correlations of H_2_-14 (δ_H_ 2.26)/H_2_-15 (δ_H_ 1.49), H_2_-15/H_2_-16 (δ_H_ 2.25), H_2_-16/H_2_-17 (δ_H_ 1.35), H_2_-17/H_2_-18 (δ_H_ 1.31), H_2_-18/H_2_-19 (δ_H_ 1.32), and H_2_-19/H_3_-20 (δ_H_ 0.90), as well as HMBC correlations from H_2_-14 to C-7 (δ_C_ 78.5) and C-8 (δ_C_ 133.3). The relative configuration of **7** was inferred to be different from that of compound **9** through a comparison of the ^13^C NMR data between **7** (C-6 δ_C_ 81.3 and C-7 δ_C_ 78.5) and **9** (C-6 δ_C_ 81.0 and C-7 δ_C_ 75.3), as well as the analysis of NOESY correlations of H-3/H_3_-11, H-4α/H-6, H-4α/H-7, H-4β/H-10, H-4β/H_3_-12, and H-10/H_3_-12 in MeOD-*d*_4_ and 3-OH/H-10 and 5-OH/H-10 in DMSO-*d*_6_ (Figure S75). The absolute configurations of **7** were subsequently assigned as 3*S*,5*R*,6*S*,7*S*,10*S* based on the similarity of its calculated and the experimental ECD spectra (Figure 6).

In addition to the seven new compounds **1**–**7**, the five known cytosporins—cytosporin X (**8**) [13], cytosporin E (**9**) [4], cytosporin L (**10**) [14], cytosporin D (**11**) [4], and cytosporin F (**12**) [3]—were also isolated and identified through a comparison of its NMR spectroscopic data with reported values in the literature.

**Figure 7:**
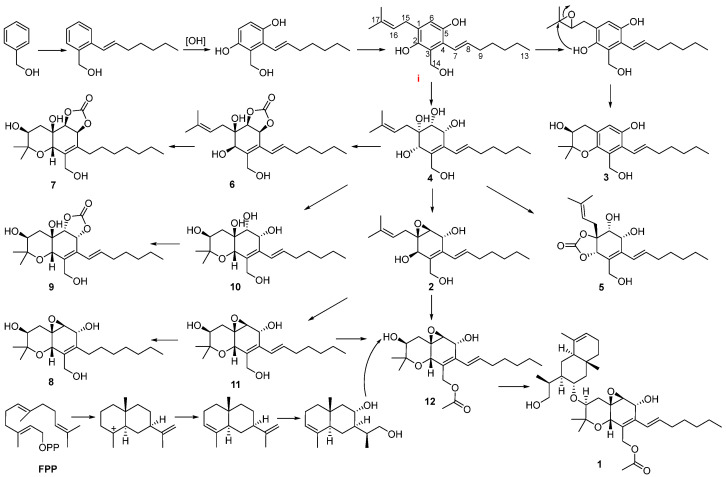
Proposed biogenesis pathway of **1**–**12**.

Structurally, considering the close relationship in biosynthesis among compounds **1**–**12**, a biosynthetic pathway different from the previous literature for these compounds is proposed (Figure 7) [3]. The possible precursor originated from phenylmethanol [3]. The subsequent addition of an isoprenyl unit, followed by hydroxylation and the addition of an aliphatic chain, would give the intermediate i. The hydroxylation of the C-1/C-6 double bond in i gave rise to the key intermediate **4**. Compound **2** was derived from **4** via a dehydration cyclization reaction. Compound **3** was generated from i via the epoxidation of the C-16/C-17 double bond and a cyclization reaction. Compounds **5** and **6** were derived from the dehydration reaction of compound **4** with carbonic acid by different attack directions and substitution positions, respectively. Compounds **7**, **10**, and **11** were obtained from **6**, **4**, and **2** via the same cyclization reaction as **3**, respectively. Compound **9** was derived from **10** via the carbonic acid substitution, while compound **8** was formed through the hydrogenation of **11**. The cyclization of compound **2**, followed by an acetylation reaction, resulted in the formation of compound **12**. Another possible precursor, the eudesmane-type sesquiterpene dihydroalanto glycol, was generated from farnesyl pyrophosphate with two steps of cyclization, dehydrogenation, and hydroxylation reaction [15]. Then, **1** was formed from the above two precursors, ii and **12**, via a condensation reaction.

All the isolated compounds **1**–**12** were evaluated for their cytotoxicity against four human cancer cell lines, including DU145, SW1990, Huh7, and PANC-1, and antibacterial activity against *Staphylococcus aureus*, *Escherichia coli*, and *Bacillus subtilis*. Unfortunately, all compounds were not active during the above test, with IC_50_ values higher than 50 μM or MIC values higher than 128 μg/mL. Additional immunosuppressive activity against ConA-induced T cell proliferation for **1**–**12** was also tested. However, only compounds **3**, **6**, **8**, and **10**–**11** displayed immunosuppressive activity, demonstrating inhibitory rates of 62.9%, 59.5%, 67.8%, 55.8%, and 68.7%, respectively, at a concentration of 5 μg/mL.

## 3. Materials and Methods

### 3.1. General Experimental Procedures

Specific rotations and IR (KBr) data were measured on a PerkinElmer model 341 polarimeter (Perkin-Elmer Inc., Waltham, MA, USA) and Jasco FTIR400 spectrometer (Jasco Inc., Tokyo, Japan), respectively. CD and UV spectra were obtained on a Jasco J-715 spectropolarimeter (Jasco Inc., Tokyo, Japan) and UV-8000 spectrophotometer (Shanghai Metash instruments Co., Shanghai, China) in MeOH, respectively. 1D and 2D NMR spectra were acquired using a Bruker AMX-500 instrument (500 MHz for ^1^H NMR, 125 MHz for ^13^C NMR) (Bruker Biospin Corp., Billerica, MA, USA) at room temperature. HRESIMS data were measured on an Agilent 6210 LC/MSD TOF mass spectrometer (Agilent Technologies Inc. Lake Forest, CA, USA). HPLC separation was performed using a YMC-Pack Pro C18 (5 μm) column (YMC Co. Ltd., Kyoto, Japan) using a Waters 1525 separation module equipped with a Waters 996 Photodiode Array (PDA) detector (Waters Corp., Milford, MA, USA). Column chromatographic purifications were performed on silica gel 60 (200–300 mesh, Qingdao Ocean Chemical Co., Qingdao, China), ODS (50 μm, YMC Co. Ltd., Kyoto, Japan), and Sephadex LH-20 (Pharmacia Co., Piscataway, NJ, USA).

### 3.2. Fungal Material

The fungus *Eutypella* sp. D-1 (GenBank accession number FJ430580) was separated from the sample collected near London Island of Kongsfjorden in the Ny-Ålesund District of the Arctic area and recognized based on 18S rDNA gene sequence analysis. The strain (No. D-1) was deposited in the Department of Marine Biomedicine and Polar Medicine, Naval Medical Center of PLA, Naval Medical University.

### 3.3. Fermentation, Extraction, and Isolation

The fungal strain *Eutypella* sp. D-1 was cultivated in seed medium (PDB 100 mL) in 250 mL Erlenmeyer flasks on a rotatory shaker (180 rpm) at 20 °C for 3 days. Subsequently, seed medium (10 mL) was transferred into 60 × 250 mL Erlenmeyer flasks (40 g of rice and 60 mL of water) and 60 plates of about 20 cm diameter (sucrose 51.4 g, NaNO_3_ 3.3 g, K_2_HPO_4_·3H_2_O 0.07 g, MgSO_4_·7H_2_O 0.4 g, KCl 0.625 g, yeast extract 0.7 g, CoCl_2_·6H_2_O 0.003125 g, FeSO_4_ 0.01875 g, CaCl_2_ 0.0065 g, and l-ornithine hydrochloride 15 g, and agar 20.0 g, dissolved in 1 L of water), respectively, and then cultured under static conditions at 20 °C for 45 days. 

The rice fermentation was combined and then extracted with CH_2_Cl_2_−MeOH (1:1, 1 L) three times. The organic solvent was concentrated under reduced pressure and partitioned with EtOAc and H_2_O to yield the EtOAc extract (24.5 g). The EtOAc extract was subjected to vacuum liquid chromatography (VLC) on silica gel via gradient elution using CH_2_Cl_2_/MeOH (80:1, 60:1, 40:1, 20:1, 15:1, 10:1, 0:1, *v*/*v*) as the solvent to give seven fractions (A–G). Fraction B (1.23 g) was chromatographed on a Sephadex LH-20 column using CH_2_Cl_2_−MeOH (1:1) as mobile phase to afford three subfractions (Fr. B1−B3), and subfraction B1 was further purified by reversed-phase HPLC eluting 43% MeCN/H_2_O at a flow rate of 2 mL/min to afford **1** (3.2 mg, t_R_ = 16.6 min). Compounds **2** (7.3 mg, t_R_ = 23.4 min) and **3** (1.4 mg, t_R_ = 46.6 min) were isolated using reversed-phase HPLC (63% MeOH/H_2_O) from subfraction B2. Fraction F (3.75 g) was separated using MPLC on an ODS (50 μm) column to give seven fractions (Fr. F1–F7). Fr. F3 was subjected to reversed-phase HPLC (65% MeOH/H_2_O, 2 mL/min) to afford **11** (15.3 mg, t_R_ = 15.3 min). Fr. F4 was separated with reversed-phase HPLC (40% CH_3_CN/H_2_O, 2 mL/min) to give **12** (40.1 mg, t_R_ = 11.3 min). 

The defined medium fermentation was combined and then extracted with CH_2_Cl_2_−MeOH (1:1, 1 L) three times. The organic solvent was concentrated under reduced pressure to yield the extract (6.86 g). The extract was subjected to silica gel VLC, eluting with a gradient of petroleum ether/EtOAc (100:1, 80:1, 50:1, 30:1, 20:1, 10:1, 5:1, 3:1, 2:1, 1:1, *v*/*v*) to obtain 20 fractions (Fr.A−Fr.T). Fraction O (0.4 g) was subjected to an ODS (50 μm) column via MPLC (MeOH/H_2_O, 50–100%) to give eight fractions, Fr. O1−Fr.O8. Fr. O6 (17.1 mg) was then purified with semipreparative HPLC (MeOH/H_2_O, 63:37, *v*/*v*; 2.0 mL/min) at 250 nm to afford **5** (6.2 mg, t_R_ = 32.1 min). Fr. P (0.42 g) was separated with MPLC (MeOH/H_2_O, 60–100%) to afford five fractions, Fr. P1−Fr. P5. Fr. P4 (27.2 mg) and Fr. P5 (19.5 mg) were purified with HPLC on an RP C18 column to give **4** (7.4 mg, MeCN/H_2_O 40:60, 2.0 mL/min, t_R_ = 24.9 min) and **6** (6.3 mg, MeCN/H_2_O 50:50, 2.0 mL/min, t_R_ = 30.1 min), respectively. Fr. Q (0.15 g) was separated with reversed-phase ODS (50 μm) MPLC eluting with a MeOH/H_2_O gradient (from 60% to 100%) to afford six subfractions, Fr.Q1−Fr.Q6. Fr. Q5 (17.2 mg) was purified on an RP C18 column with HPLC (80% MeOH/H_2_O, 2.0 mL/min), yielding **2** (2.4 mg, t_R_ = 28.8 min). Fr. R (1.04 g) was chromatographed over ODS via MPLC using a gradient elution of MeOH−H_2_O (from 50% to 100%) to get five fractions (Fr. R1−R5). Fr. R3 (475.1 mg) was then subjected to a silica gel CC (petroleum ether/EtOAc, 3:1, *v*:*v*) to give five fractions, Fr. R3a−Fr. R3e. Fr. R3c (168.0 mg) was then purified with semipreparative HPLC on an RP C18 ODS (CH_3_CN/H_2_O, 30:70, *v*/*v*; 2.0 mL/min) to afford **8** (3.5 mg, t_R_ = 52.2 min) and **11** (116.8 mg, t_R_ = 60.5 min). Fr. R3d (275.7 mg) was further purified with 37% CH_3_CN via HPLC (2.0 mL/min) to afford **7** (6.8 mg, t_R_ = 33.0 min) and **9** (174.0 mg, t_R_ = 39.8 min). Fr. S (0.55 g) was chromatographed over ODS using a gradient elution of MeOH/H_2_O (from 50% to 100%) to obtain three fractions (Fr. S1−S3). Fr. S3 (322.0 mg) was further purified with 35% CH_3_CN via HPLC to afford compound **10** (243.8 mg, t_R_ = 18.6 min).

Eutypelleudesmane A (**1**): light-brown oil; ${\left[\alpha \right]}_{D}^{25}$ –23.0 (*c* 0.10, MeOH); UV (MeOH) (log *ε*) λ_max_ 241 (4.07) nm; CD (MeOH) (Δ*ε*) 242 (+17.1); IR (KBr) *ν*_max_ 3357, 2956, 2929, 2873, 1741, 1650, 1455, 1438, 1376, 1232, 1153, 1116, 1068, 1024, 958, 883,850,719 cm^−1^; ^1^H and ^13^C NMR data, see Table 1; HRESIMS *m/z* 601.4074 [M + H]^+^ (calcd for C_36_H_57_O_7_, 601.4104).

Cytosporin Y (**2**): light brown oil; ${\left[\alpha \right]}_{D}^{25}$ +14.0 (*c* 0.10, MeOH); UV (MeOH) (log ε) λ_max_ 241 (3.89) nm; CD (MeOH) (Δε) 238 (+9.1); IR (KBr) *ν*_max_ 3359, 2956, 2927, 2857, 1454, 1376, 1261, 1014, 842, 725 cm^−1^; ^1^H and ^13^C NMR data, see Table 2; HRESIMS *m/z* 367.2125 [M + COOH]^−^ (calcd for C_20_H_31_O_6_, 367.2121).

Cytosporin Z (**3**): light-brown oil; ${\left[\alpha \right]}_{D}^{25}$ +12.3 (*c* 0.10, MeOH), ${\left[\alpha \right]}_{D}^{25}$ +1.9 (*c* 0.1, CDCl_3_); UV (MeOH) (log ε) λ_max_ 210 (5.37), 312 (3.13) nm; IR (KBr) *ν*_max_ 3378, 2954, 2927, 2856, 1708, 1614, 1513, 1434, 1380, 1369, 1255, 1218, 1184, 1143, 1064, 1029, 977, 852 cm^−1^; ^1^H and ^13^C NMR data, see Table 2; HRESIMS *m/z* 319.1912 [M − H]^−^ (calcd for C_19_H_27_O_4_, 319.1909).

Cytosporin Y_1_ (**4**): light-yellow oil; ${\left[\alpha \right]}_{D}^{25}$ –15.8 (*c* 0.1, MeOH); UV (MeOH) λ_max_ (log ε) 221 (3.71), 240 (3.77) nm; IR ν_max_ 3367, 2955, 2926, 2857, 1743, 1601, 1378, 1072, 1023 cm^−1^; CD (MeOH) (Δε) 208 (–6.4); ^1^H NMR and ^13^C NMR, see Table 3; HRESIMS *m/z* 363.2139 [M + Na]^+^ (calcd for C_19_H_32_O_5_Na, 363.2142).

Cytosporin Y_2_ (**5**): light-yellow oil; ${\left[\alpha \right]}_{D}^{25}$ –60.1 (*c* 0.1, MeOH); UV (MeOH) λ_max_ (log ε) 214 (3.96), 244 (4.28) nm; IR ν_max_ 3381, 2956, 2926, 2857, 1786, 1647, 1344, 1219, 1049, 1023, 1001, 822, 760 cm^−1^; CD (MeOH) (Δε) 222 (–13.2); ^1^H NMR and ^13^C NMR, see Table 3; HRESIMS *m/z* 389.1928 [M + Na]^+^ (calcd for C_20_H_30_O_6_Na, 389.1935).

Cytosporin Y_3_ (**6**): light-yellow oil, ${\left[\alpha \right]}_{D}^{25}$ +37.8 (*c* 0.1, MeOH); UV (MeOH) λ_max_ (log ε) 217 (3.64), 241 (3.81) nm; IR ν_max_ 3383, 2956, 2928, 2858, 1783, 1595, 1361, 1270, 1182, 1068, 907, 770, 737 cm^−1^; CD (MeOH) (Δε) 241 (+4.87); ^1^H NMR and ^13^C NMR, see Table 4; HRESIMS *m/z* 389.1928 [M + Na]^+^ (calcd for C_20_H_30_O_6_Na, 389.1935).

Cytosporin E_1_ (**7**): light-yellow oil, ${\left[\alpha \right]}_{D}^{25}$ +12.9 (*c* 0.1, MeOH), UV (MeOH) λ_max_ (log ε) 200 (4.10) nm; IR ν_max_ 3393, 2926, 2856, 1783, 1464, 1361, 1184, 1158, 1086, 1058, 1023, 917, 772, 629 cm^−1^; CD (MeOH) (Δε) 210 (+14.2) nm; ^1^H NMR and ^13^C NMR, see Table 4; HRESIMS *m/z* 363.2145 407.2034 [M + Na]^+^ (calcd for C_20_H_32_O_7_Na, 407.2040).

### 3.4. Biological Assay

The antimicrobial activities of compounds **1**–**12** against *Staphylococcus aureus*, *Escherichia coli*, and *Bacillus subtilis* were evaluated using a previous method [16], and levofloxacin was used as a positive control. The cytotoxicities of compounds **1**–**12** against DU145, SW1990, Huh7, and PANC-1 human cancer cell lines were determined using the CCK-8 method [17], with cisplatin used as a positive control. The immunosuppressive activities of compounds **1**–**12** against ConA-induced T cell proliferation were performed as previously described [18], with cyclosporin A as a positive control.

## 4. Conclusions

In summary, the utilization of the OSMAC (one strain many compounds) culture strategy effectively modified the chemical profile of the Arctic-derived fungus *Eutypella* sp. D-1 when cultivated in different media. This approach resulted in the production of five cytosporin polyketides (compounds **1**–**3** and **11**–**12**) from a rice medium and eight cytosporins (compounds **2** and **4**–**11**) from a solid defined medium. Remarkably, compound **1** contained a unique skeleton formed by the ester linkage of two moieties: cytosporin F (**12**) and the eudesmane-type sesquiterpene dihydroalanto glycol. Compounds **6** and **7**, characterized by a cyclic carbonate-fused cytosporin skeleton, were found to be rare in nature. However, these metabolites only exhibited weak immunosuppressive inhibitory activity against ConA-induced T cell proliferation in the antimicrobial, cytotoxic, and immunosuppressive evaluation. Collectively, this work showcased that changing the fermentation medium could be an effective strategy to trigger the production of secondary metabolites from fungi derived from the polar extreme environment.

## Figures and Tables

**Table 1:** ^1^H (500 MHz) and ^13^C NMR (125 MHz) spectroscopic data of **1** in CDCl_3_.

Position	δ_C_	δ_H_, mult. (*J* in Hz)	Position	δ_C_	δ_H_, mult. (*J* in Hz)
2	76.6, C		20	14.0, CH_3_	0.89, t (7.0)
3	73.8, CH	3.72, m	21	171.0, C	
4α	35.5, CH_2_	1.72, dd (12.5, 5.0)	22	20.9, CH_3_	2.08, s
4β		2.27, t (12.0)	23	37.5, CH_2_	1.35, m
5	55.7, C		24	22.7, CH_2_	1.99, m
6	59.7, CH	3.32, s	25	121.2, CH	5.31, brs
7	64.6, CH	4.73, s	26	134.1, C	
8	135.6, C		27	46.3, CH	1.97, m
9	125.1, C		28α	28.5, CH_2_	1.66, m
10	67.5, CH	4.38, s	28β		1.23, m
11	16.0, CH_3_	1.30, s	29	49.7, CH	1.50, m
12	27.7, CH_3_	1.29, s	30	66.7, CH	3.87, td (11.0, 5.0)
13a	61.5, CH_2_	4.67, d (12.5)	31α	49.4, CH_2_	1.15, m
13b		4.81, d (12.5)	31β		1.79, dd (12.5, 5.0)
14	124.7, CH	6.33, d (16.0)	32	33.7, C	
15	136.4, CH	6.16, m	33	39.0, CH	1.87, m
16	33.5, CH_2_	2.17, m	34	67.5, CH_2_	3.67, dd (7.5, 4.0)
17	28.8, CH_2_	1.42, m	35	11.9, CH_3_	1.01, d (7.0)
18	31.4, CH_2_	1.27, m	36	16.5, CH_3_	0.79, s
19	22.5, CH_2_	1.28, m	37	21.2, CH_3_	1.61, s

**Table 2:** ^1^H (500 MHz) and ^13^C NMR (125 MHz) spectroscopic data of **2** and **3** in CDCl_3_.

2			3		
Position	δ_C_	δ_H_, mult. (*J* in Hz)	Position	δ_C_	δ_H_, mult. (*J* in Hz)
1	59.3, C		2	77.2, C	
2	57.5, CH	3.29, s	3	69.7, CH	3.79, t (5.0)
3	64.3, CH_2_	4.72, s	4α	31.5, CH_2_	3.03, dd (17.0, 5.0)
4	131.6, C		4β		2.72, dd (17.0, 5.0)
5	131.4, C		5	118.8, C	
6	69.5, CH	4.45, s	6	115.1, CH	6.61, s
7	124.6, CH	6.28, d (16.0)	7	146.7, C	
8	135.4, CH	6.05, m	8	123.6, C	
9	33.5, CH_2_	2.15, m	9	126.8, C	
10	28.9, CH_2_	1.41, m	10	144.8, C	
11	31.5, CH_2_	1.28, m	11	22.4, CH_3_	1.36, s
12	22.5, CH_2_	1.29, m	12	24.9, CH_3_	1.32, s
13	14.0, CH_3_	0.88, t (6.0)	13	58.8, CH_2_	4.66, s
14a	62.2, CH_2_	4.57, d (12.0)	14	122.7, CH	6.35, d (16.5)
14b		4.06, d (12.0)	15	140.0, CH	5.95, m
15α	29.7, CH_2_	2.82, dd (15.0, 8.0)	16	33.4, CH_2_	2.27, m
15β		2.30, dd (15.0, 8.0)	17	29.0, CH_2_	1.50, m
16	117.3, CH	5.20, t (7.0)	18	31.5, CH_2_	1.35, m
17	135.9, C		19	22.6, CH_2_	1.35, m
18	18.0, CH_3_	1.66, s	20	14.1, CH_3_	0.91, t (7.0)
19	25.9, CH_3_	1.73, s			

**Table 3:** ^1^H (500 MHz) and ^13^C NMR (125 MHz) spectroscopic data of **4** and **5** in MeOD-*d*_4_.

4			5	
Position	δ_C_	δ_H_, mult. (*J* in Hz)	δ_C_	δ_H_, mult. (*J* in Hz)
1	74.3, C		84.4, C	
2	75.0, CH	3.76, d (4.5)	71.3, CH	3.94, d (4.5)
3	69.6, CH	4.49, d (4.5)	68.6, CH	4.41, d (4.5)
4	135.8, C		138.3, C	
5	134.5, C		127.3, C	
6	73.6, CH	3.88, s	76.9, CH	5.15, s
7	126.6, CH	6.24, d (16.0)	124.8, CH	6.41, d (16.0)
8	137.2, CH	6.01, m	136.7, CH	6.12, dt (16.0, 7.0)
9	35.0, CH_2_	2.17, m	33.1, CH_2_	2.21, m
10	30.5, CH_2_	1.46, m	28.6, CH_2_	1.47, m
11	32.9, CH_2_	1.28, m	31.2, CH_2_	1.33, m
12	23.9, CH_2_	1.34, m	22.2, CH_2_	1.33, m
13	14.7, CH_3_	0.91, t (7.0)	13.0, CH_3_	0.91, t (7.0)
14a	61.1, CH_2_	4.25, d (13.0)	58.1, CH_2_	4.13, d (13.0)
14b		4.38, d (13.0)		4.51, d (13.0)
15a	35.7, CH_2_	2.60, m	31.8, CH_2_	2.53, dd (15.0, 8.5)
15b				2.66, dd (15.0, 7.0)
16	119.8, CH	5.40, m	115.3, CH	5.25, m
17	136.1, C		137.7, C	
18	26.7, CH_3_	1.76, s	24.9, CH_3_	1.77, s
19	18.6, CH_3_	1.70, s	16.9, CH_3_	1.68, s
20			154.8, C	

**Table 4:** ^1^H (500 MHz) and ^13^C NMR (125 MHz) spectroscopic data of **6** and **7** in CDCl_3_.

6			7		
Position	δ_C_	δ_H_, mult. (*J* in Hz)	Position	δ_C_	δ_H_, mult. (*J* in Hz)
1	72.3, C		2	77.7, C	
2	79.2, CH	4.71, dd (8.0, 2.0)	3	71.6, CH	3.96, dd (12.0, 5.0)
3	75.2, CH	5.55, d (8.0)	4α	42.7, CH_2_	1.89, dd (12.0, 5.0)
4	129.1, C		4β		2.25, d (12.0)
5	139.4, C		5	68.0, C	
6	71.5, CH	4.14, d (2.0)	6	81.3, CH	4.66, dd (8.0, 2.0)
7	126.1, CH	6.52, d (16.0)	7	78.5, CH	5.23, d (8.0)
8	136.6, CH	6.03, dt (16.0, 7.0)	8	133.3, C	
9	34.9, CH_2_	2.21, dd (14.0, 7.0)	9	135.6, C	
10	30.4, CH_2_	1.45, m	10	69.7, CH	4.17, d (2.0)
11	32.9, CH_2_	1.34, m	11	16.9, CH3	1.26, s
12	23.9, CH_2_	1.34, m	12	28.5, CH_3_	1.21, s
13	14.7, CH_3_	0.91, t (7.0)	13a	60.2, CH_2_	4.09, d (12.0)
14a	60.8, CH_2_	4.29, d (12.5)	13b		4.29, d, (12.0)
14b		4.43, d (12.5)	14	31.0, CH_2_	2.26, m
15a	35.0, CH_2_	2.51, dd (15.0, 6.5)	15	30.0, CH_2_	1.49, m
15b		2.71, dd (15.0, 8.5)	16	31.1, CH_2_	2.25, m
16	118.8, CH	5.38, m	17	30.5, CH_2_	1.35, m
17	136.9, C		18	33.3, CH_2_	1.31, m
18	26.7, CH_3_	1.77, s	19	24.0, CH_2_	1.32, m
19	18.6, CH_3_	1.71, s	20	14.7, CH_3_	0.9, t (7.0)
20	156.8, C		21	156.3, C	

## Data Availability

The data presented in this study are available on request from the corresponding author.

## Supplementary Materials

The following are available online at https://www.mdpi.com/article/10.3390/md21070382/s1, 1D and 2D NMR, UV, IR, and HRESMS data of **1**–**7**.
