# Supplementary material for: Cytosporin Derivatives from Arctic-Derived Fungus Eutypella sp. D-1 via the OSMAC Approach
Source: Mar Drugs. 2023 Jun 28;21(7):382. doi: 10.3390/md21070382 (PMC10381684; doi:10.3390/md21070382)

Supplementary data

Cytosporin Derivatives from Arctic-derived Fungus *Eutypella* sp. D-1 by OSMAC Approach

Hao-Bing Yu [1,†], Zhe Ning [1,†], Bo Hu [1],Yu-Ping Zhu [2], Xiao-Ling Lu [3], Ying He [1], Bing-Hua Jiao [3], and Xiao-Yu Liu [1,*]

1 Department of Marine Biomedicine and Polar Medicine, Naval Medical Center of PLA, Naval Medical Uni-versity, Shanghai 200433, China; yuhaobing1986@126.com (H.-B.Y.); Ningzhe95@163.com (Z.N.); hb8601@163.com (B.H.); yinghe_hys@163.com (Y.H.)

2 Basic Medical Experimental Teaching Center, College of Basic Medical Sciences, Naval Medical University, Shanghai 200433, China; zhuyuping72@hotmail.com (Y.-P.Z.);

3 Department of Biochemistry and Molecular Biology, College of Basic Medical Sciences, Naval Medical University, Shanghai 200433, China; luxiaoling80@126.com (X.-L.L.); jiaobh@live.cn (B.-H. J.)

* Correspondence: biolxy@163.com; Tel.: +86-21-81870970

† These authors contributed equally to this work.

S1. Quantum chemical CD calculation of compound **4**.
S2. Quantum chemical CD calculation of compound **5**.
S3. Quantum chemical CD calculation of compound **6**.
S4. Quantum chemical CD calculation of compound **7**.
S5. ^{1}H NMR spectrum of eutypelleudesmane A (**1**) in $CDCl_3$.
S6. ^{13}C NMR spectrum of eutypelleudesmane A (**1**) in $CDCl_3$.
S7. DEPT135 spectrum of eutypelleudesmane A (**1**) in $CDCl_3$.
S8. HSQC spectrum of eutypelleudesmane A (**1**) in $CDCl_3$.
S9. COSY spectrum of eutypelleudesmane A (**1**) in $CDCl_3$.
S10. HMBC spectrum of eutypelleudesmane A (**1**) in $CDCl_3$.
S11. NOESY spectrum of eutypelleudesmane A (**1**) in $CDCl_3$.
S12. HRESIMS of eutypelleudesmane A (**1**).
S13. UV spectrum of eutypelleudesmane A (**1**) in MeOH.
S14. IR spectrum of eutypelleudesmane A (**1**) (KBr).
S15. ^{1}H NMR spectrum of cytosporin X (**2**) in $CDCl_3$.
S16. ^{1}H NMR spectrum of cytosporin X (**2**) in DMSO-d_6.
S17. ^{13}C NMR spectrum of cytosporin X (**2**) in $CDCl_3$.
S18. DEPT135 spectrum of cytosporin X (**2**) in $CDCl_3$.
S19. HSQC spectrum of cytosporin X (**2**) in $CDCl_3$.
S20. COSY spectrum of cytosporin X (**2**) in $CDCl_3$.
S21. HMBC spectrum of cytosporin X (**2**) in $CDCl_3$.
S22. NOESY spectrum of cytosporin X (**2**) in $CDCl_3$.
S23. NOESY spectrum of cytosporin X (**2**) in DMSO-d_6.
S24. HRESIMS of cytosporin X (**2**).
S25. UV spectrum of cytosporin X (**2**) in MeOH.
S26 IR spectrum of cytosporin X (**2**) (KBr).
S27. ^{1}H NMR spectrum of cytosporin Y (**3**) in $CDCl_3$.
S28. ^{13}C NMR spectrum of cytosporin Y (**3**) in $CDCl_3$.
S29. DEPT135 spectrum of cytosporin Y (**3**) in $CDCl_3$.
S30. HSQC spectrum of cytosporin Y (**3**) in $CDCl_3$.
S31. COSY spectrum of cytosporin Y (**3**) in $CDCl_3$.
S32. HMBC spectrum of cytosporin Y (**3**) in $CDCl_3$.
S33. NOESY spectrum of cytosporin Y (**3**) in $CDCl_3$.
S34. HRESIMS of cytosporin Y (**3**).
S35. UV spectrum of cytosporin Y (**3**) in MeOH.
S36. IR spectrum of cytosporin Y (**3**) (KBr).
S37. ^{1}H NMR spectrum of cytosporin Y_1 (**4**) in MeOD-d_4.
S38. ^{13}C NMR spectrum of cytosporin Y_1 (**4**) in MeOD-d_4.
S39. DEPT135 spectrum of cytosporin Y_1 (**4**) in MeOD-d_4.
S40. HSQC spectrum of cytosporin Y_1 (**4**) in MeOD-d_4.
S41. COSY spectrum of cytosporin Y_1 (**4**) in MeOD-d_4.
S42. HMBC spectrum of cytosporin Y_1 (**4**) in MeOD-d_4.
S43. NOESY spectrum of cytosporin Y_1 (**4**) in MeOD-d_4.
S44. HRESIMS of cytosporin Y_1 (**4**).

S45. UV spectrum of cytosporin Y_1 (**4**) in MeOH.
S46. IR spectrum of cytosporin Y_1 (**4**) (KBr).
S47. ^{1}H NMR spectrum of cytosporin Y_2 (**5**) in MeOD-d_4.
S48. ^{13}C NMR spectrum of cytosporin Y_2 (**5**) in MeOD-d_4.
S49. DEPT135 spectrum of cytosporin Y_2 (**5**) in MeOD-d_4.
S50. HSQC spectrum of cytosporin Y_2 (**5**) in MeOD-d_4.
S51. COSY spectrum of cytosporin Y_2 (**5**) in MeOD-d_4.
S52. HMBC spectrum of cytosporin Y_2 (**5**) in MeOD-d_4.
S53. NOESY spectrum of cytosporin Y_2 (**5**) in MeOD-d_4.
S54. HRESIMS of cytosporin Y_2 (**5**).
S55. UV spectrum of cytosporin Y_2 (**5**) in in MeOH.
S56. IR spectrum of cytosporin Y_2 (**5**) (KBr).
S57. ^{1}H NMR spectrum of cytosporin Y_3 (**6**) in $CDCl_3$.
S58. ^{13}C NMR spectrum of cytosporin Y_3 (**6**) in $CDCl_3$.
S59. DEPT135 spectrum of cytosporin Y_3 (**6**) in $CDCl_3$.
S60. HSQC spectrum of cytosporin Y_3 (**6**) in $CDCl_3$.
S61. COSY spectrum of cytosporin Y_3 (**6**) in $CDCl_3$.
S62. HMBC spectrum of cytosporin Y_3 (**6**) in $CDCl_3$.
S63. NOESY spectrum of cytosporin Y_3 (**6**) in $CDCl_3$.
S64. HRESIMS of cytosporin Y_3 (**6**).
S65. UV spectrum of cytosporin Y_3 (**6**) in MeOH.
S66. IR spectrum of cytosporin Y_3 (**6**) (KBr).
S67. ^{1}H NMR spectrum of cytosporin E_1 (**7**) in $CDCl_3$.
S68. ^{1}H NMR spectrum of cytosporin E_1 (**7**) in DMSO-d_6.
S69. ^{13}C NMR spectrum of cytosporin E_1 (**7**) in $CDCl_3$.
S70. DEPT135 spectrum of cytosporin E_1 (**7**) in $CDCl_3$.
S71. HSQC spectrum of cytosporin E_1 (**7**) in $CDCl_3$.
S72. COSY spectrum of cytosporin E_1 (**7**) in $CDCl_3$.
S73. HMBC spectrum of cytosporin E_1 (**7**) in $CDCl_3$.
S74. NOESY spectrum of cytosporin E_1 (**7**) in $CDCl_3$.
S75. NOESY spectrum of cytosporin E_1 (**7**) in DMSO-d_6.

S1. Quantum chemical CD calculation of compound **4**.

Conformational analysis was initially performed using Spartan'10 software (Wavefunction, Inc., Irvine, CA, USA) at MMFF94 force field. The conformers with Boltzmann-population of over 5% were chosen for ECD calculations, and then the conformers were initially optimized at B3LYP/DGDZVP level in MeOH using the continuum polarizable continuum model (CPCM). Harmonic vibration frequencies were calculated to confirm the stability of these conformers. As revealed by the frequency analysis, no imaginary frequencies were observed in ground states. The theoretical calculation of ECD was conducted in MeOH using Time-dependent Density functional theory (TD-DFT) at the CAM-B3LYP/DGDZVP level for all conformers of compound **4**. The CD spectra were generated by the program GaussView 6.0 (University of Würzburg, Würzburg, Germany) using a Gaussian band shape with 0.3 eV exponential half-width from dipole-length dipolar and rotational strengths.

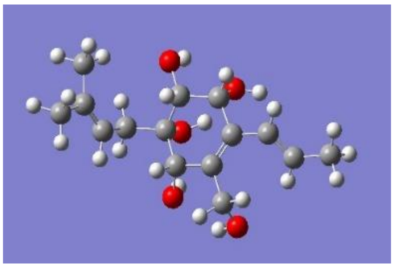

4a

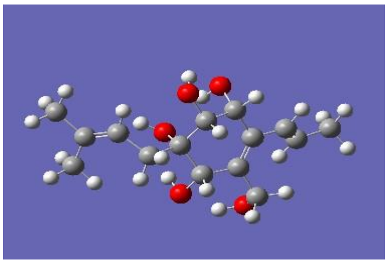

4b

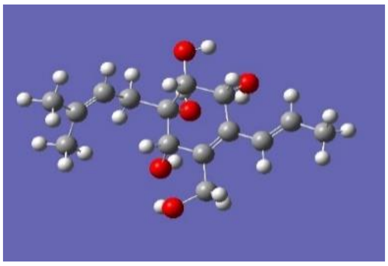

4c

	Conformer	ΔE(kcal/mol)	Population(%)
compound Model-**4**	**4a**	0	73.6
	**4b**	0.91	15.8
	**4c**	1.15	10.6

S2. Quantum chemical CD calculation of compound **5**.

Conformational analysis was initially performed using Spartan'10 software (Wavefunction, Inc., Irvine, CA, USA) at MMFF94 force field. The conformers with Boltzmann-population of over 5% were chosen for ECD calculations, and then the conformers were initially optimized at B3LYP/DGDZVP level in MeOH using the continuum polarizable continuum model (CPCM). Harmonic vibration frequencies were calculated to confirm the stability of these conformers. As revealed by the frequency analysis, no imaginary frequencies were observed in ground states. The theoretical calculation of ECD was conducted in MeOH using Time-dependent Density functional theory (TD-DFT) at the CAM-B3LYP/DGDZVP level for all conformers of compound **5**. The CD spectra were generated by the program GaussView 6.0 (University of Würzburg, Würzburg, Germany) using a Gaussian band shape with 0.3 eV exponential half-width from dipole-length dipolar and rotational strengths.

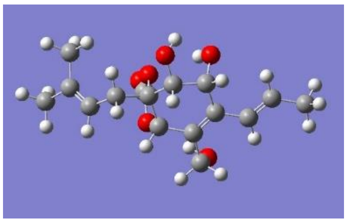

5a

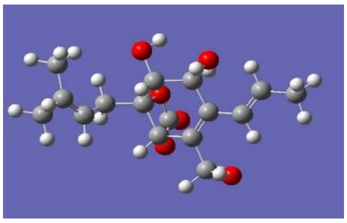

5b

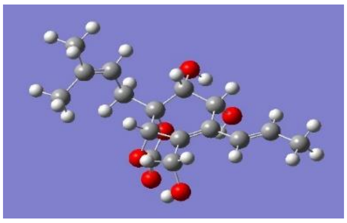

5c

	Conformer	ΔE(kcal/mol)	Population(%)
compound Model-**5**	**5a**	0	65.0
	**5b**	0.58	24.4
	**5c**	1.07	10.6

S3. Quantum chemical CD calculation of compound **6**.

Conformational analysis was initially performed using Spartan'10 software (Wavefunction, Inc., Irvine, CA, USA) at MMFF94 force field. The conformers with Boltzmann-population of over 5% were chosen for ECD calculations, and then the conformers were initially optimized at B3LYP/DGDZVP level in MeOH using the continuum polarizable continuum model (CPCM). Harmonic vibration frequencies were calculated to confirm the stability of these conformers. As revealed by the frequency analysis, no imaginary frequencies were observed in ground states. The theoretical calculation of ECD was conducted in MeOH using Time-dependent Density functional theory (TD-DFT) at the CAM-B3LYP/DGDZVP level for all conformers of compound **6**. The CD spectra were generated by the program GaussView 6.0 (University of Würzburg, Würzburg, Germany) using a Gaussian band shape with 0.3 eV exponential half-width from dipole-length dipolar and rotational strengths.

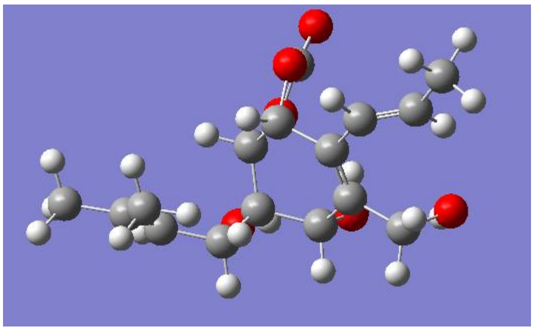

6a

compound Model-**6a**	Conformer	ΔE(kcal/mol)	Population(%)
	1	0	99.9%

S4. Quantum chemical CD calculation of compound **7**.

Quantum chemical CD calculation of compound **7** Conformational analysis was initially performed using Spartan'10 software (Wavefunction, Inc., Irvine, CA, USA) at MMFF94 force field. The conformers with Boltzmann-population of over 5% were chosen for ECD calculations, and then the conformers were initially optimized at B3LYP/DGDZVP level in MeOH using the continuum polarizable continuum model (CPCM). Harmonic vibration frequencies were calculated to confirm the stability of these conformers. As revealed by the frequency analysis, no imaginary frequencies were observed in ground states. The theoretical calculation of ECD was conducted in MeOH using Time-dependent Density functional theory (TD-DFT) at the CAM-B3LYP/DGDZVP level for all conformers of compound **7**. The CD spectra were generated by the program GaussView 6.0 (University of Würzburg, Würzburg, Germany) using a Gaussian band shape with 0.3 eV exponential half-width from dipole-length dipolar and rotational strengths.

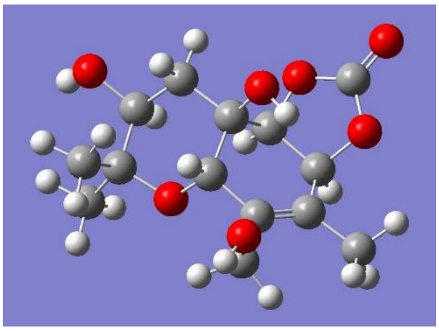

7a

compound Model-**7**	Conformer	ΔE(kcal/mol)	Population(%)
	**7a**	0	99.9%

S5. ^{1}H NMR spectrum of eutypelleudesmane A (**1**) in $CDCl_3$.

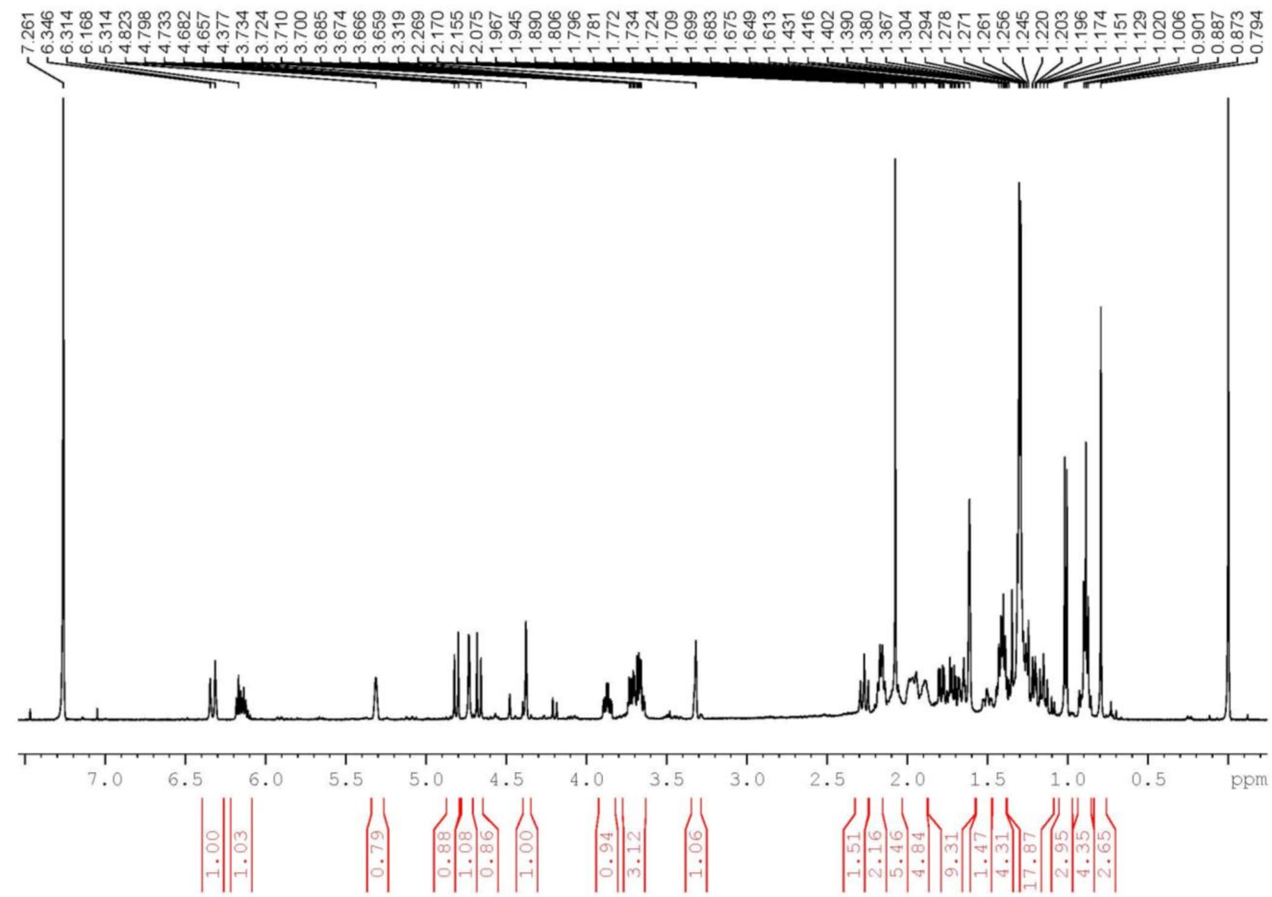

S6. ^{13}C NMR spectrum of eutypelleudesmane A (**1**) in $CDCl_3$.

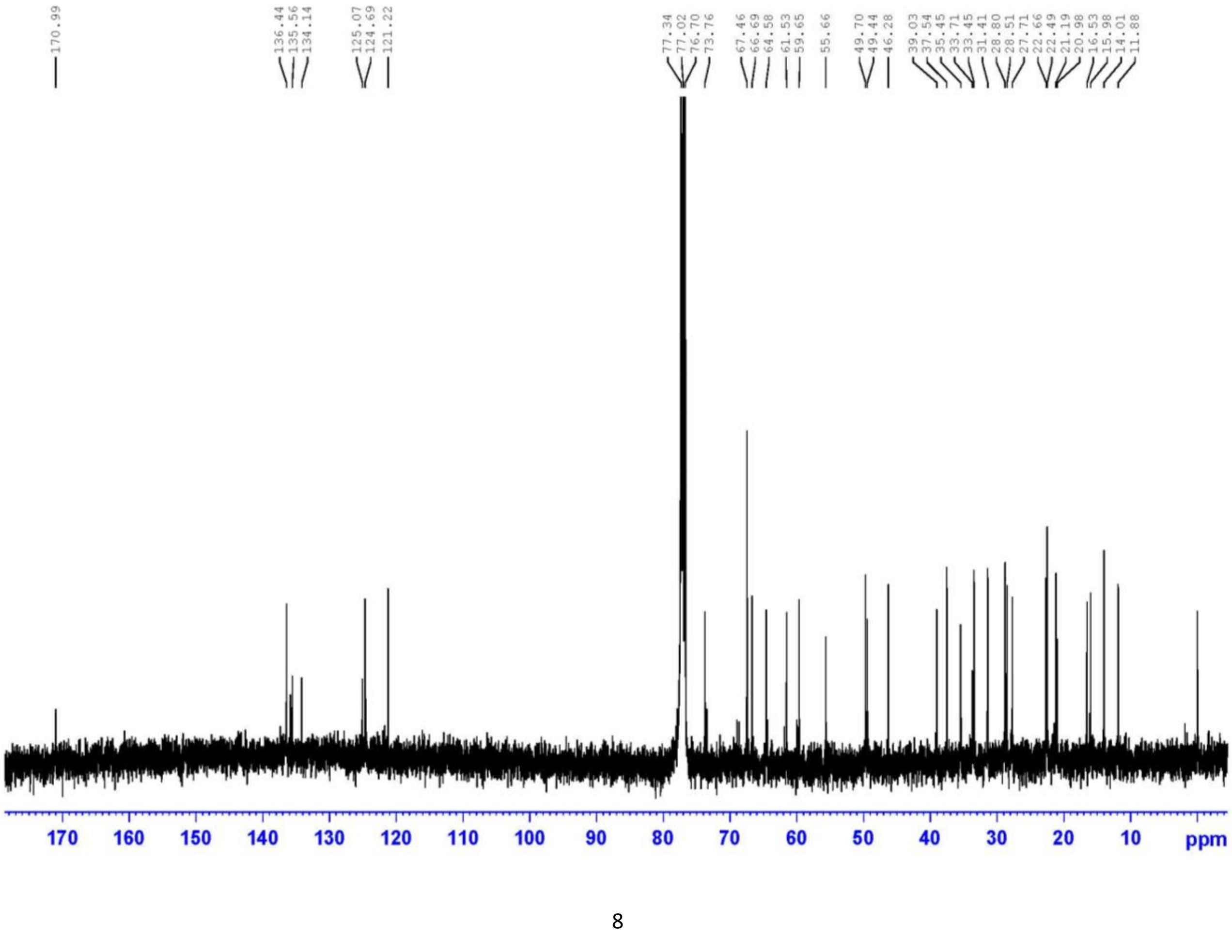

S7. DEPT135 spectrum of eutypelleudesmane A (**1**) in $CDCl_3$.

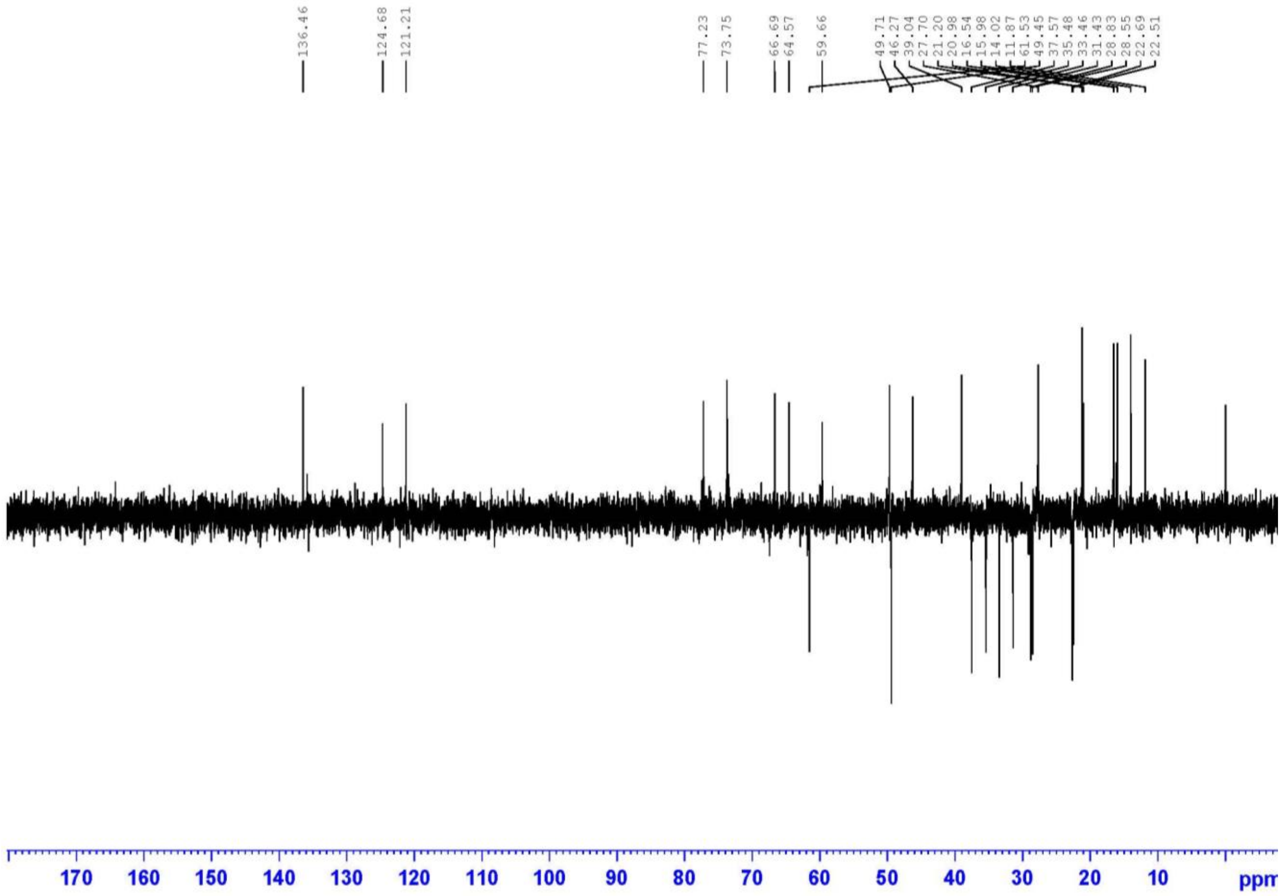

S8. HSQC spectrum of eutypelleudesmane A (**1**) in $CDCl_3$.

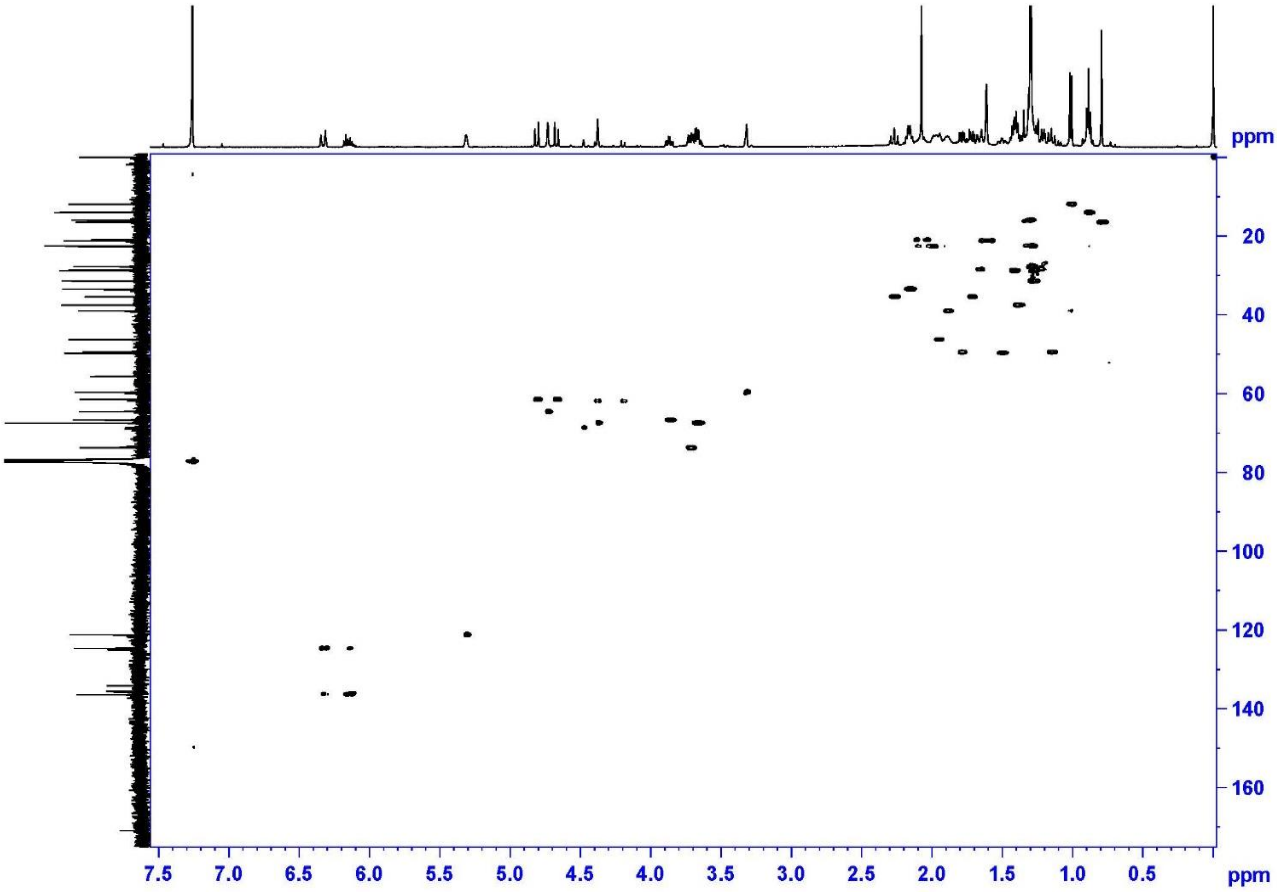

S9. COSY spectrum of eutypelleudesmane A (**1**) in $CDCl_3$.

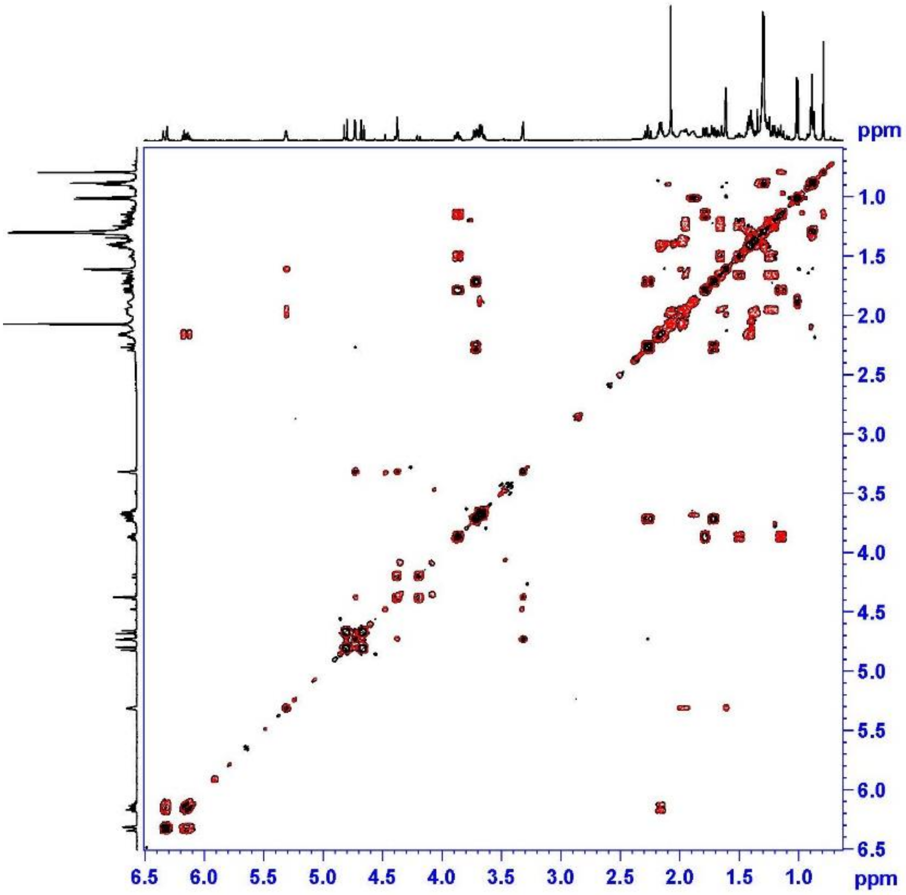

S10. HMBC spectrum of eutypelleudesmane A (**1**) in $CDCl_3$.

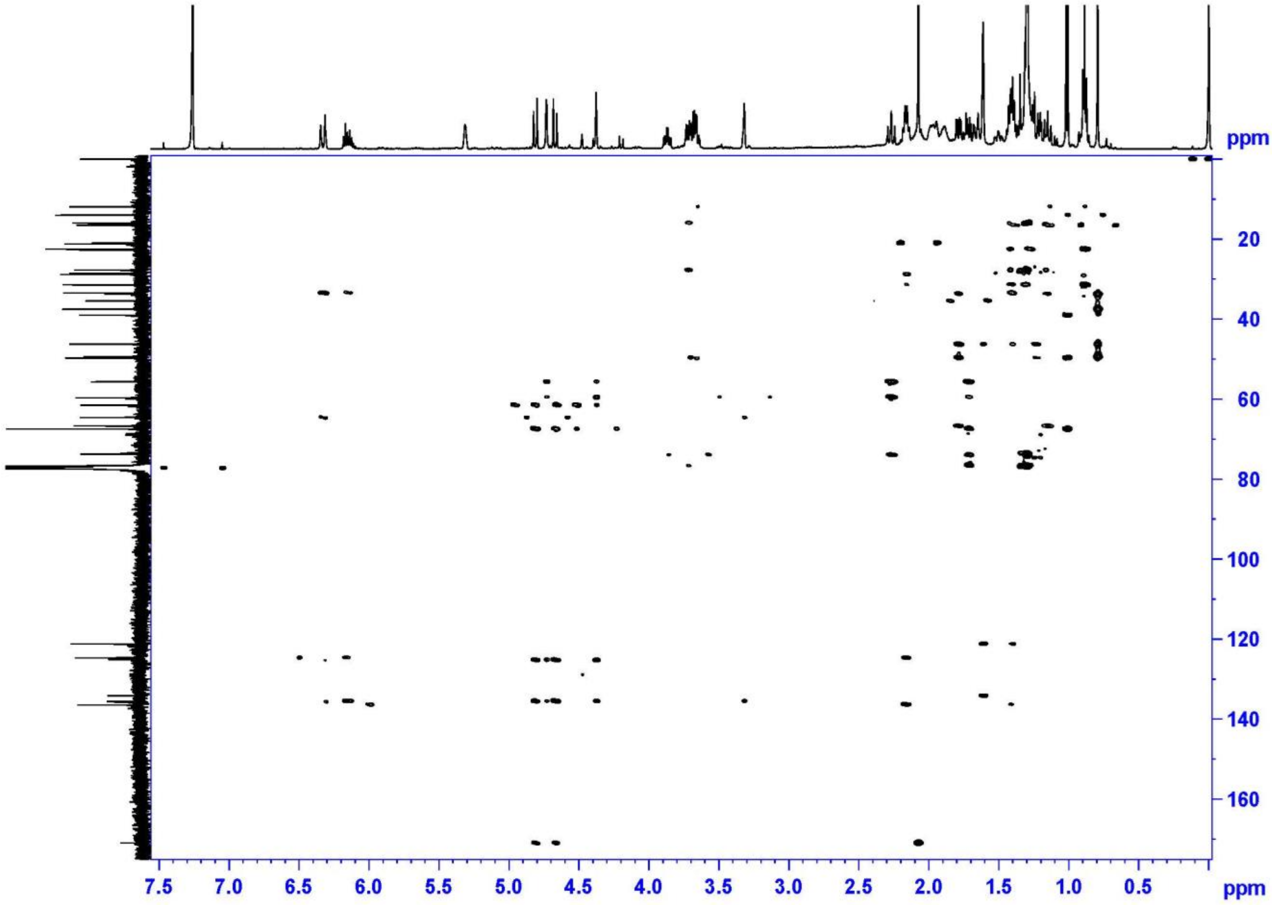

S11. NOESY spectrum of eutypelleudesmane A (**1**) in $CDCl_3$.

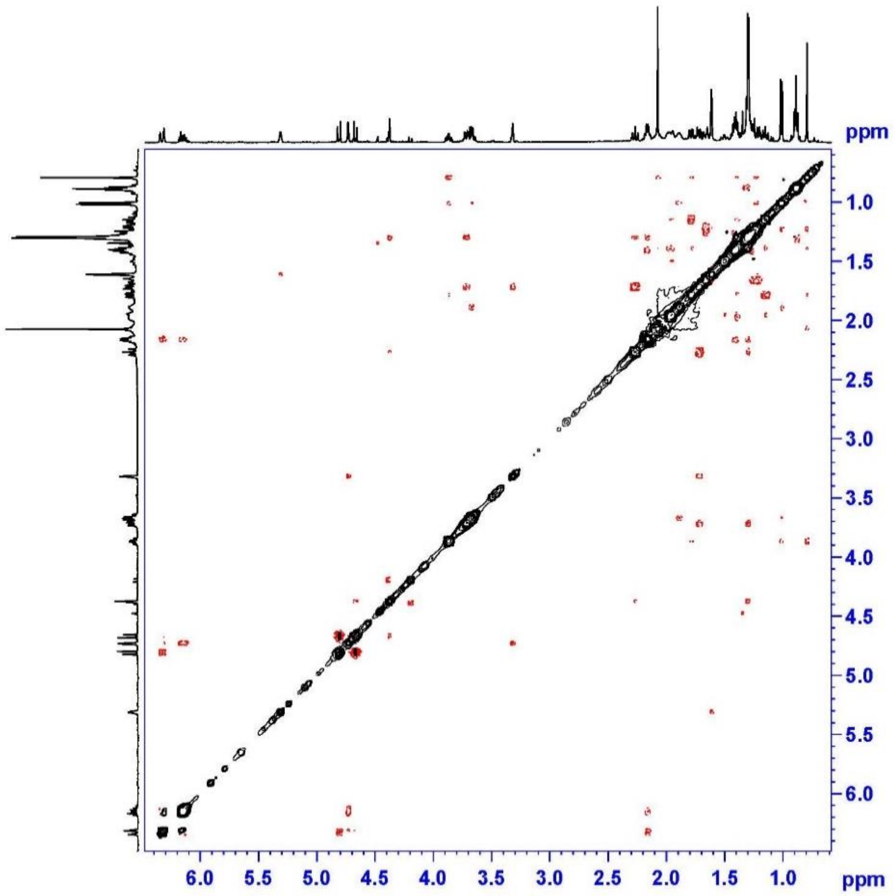

S12. HRESIMS of eutypelleudesmane A (**1**).

Qualitative Analysis Report

**Data Filename**	W-44-POS.d	**Sample Name**	
**Sample Type**	Sample	**Position**	P1-F4
**Instrument Name**	Instrument 1	**User Name**	
**Acq Method**	SERUM-POS-15MIN.m	**Acquired Time**	
**IRM Calibration Status**	Success	**DA Method**	E.m

User Spectra

Fragmentor Voltage	Collision Energy	Ionization Mode
120	0	ESI

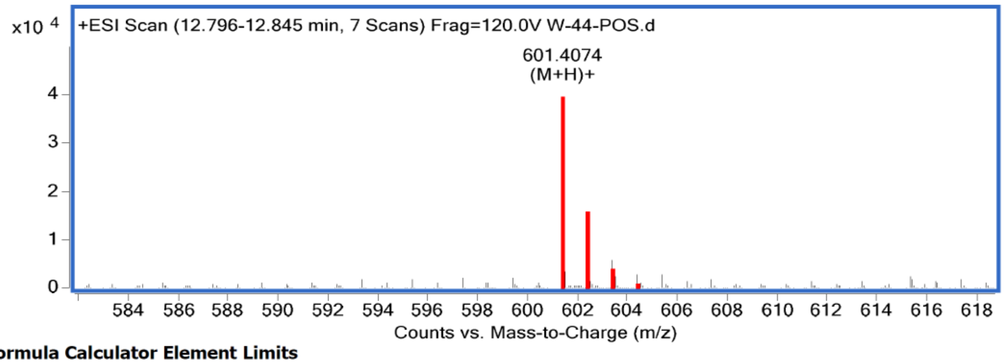

Formula Calculator Element Limits

Element	Min	Max
C	0	50
H	0	400
O	0	20

Formula Calculator Results

Formula	Best	Mass	Tgt Mass	Diff (ppm)	Ion Species	Score
C36 H57 O7	TRUE	601.4074	601.4104	4.9	C36 H57 O7	83.16

--- End Of Report ---

S13. UV spectrum of eutypelleudesmane A (**1**) in MeOH.

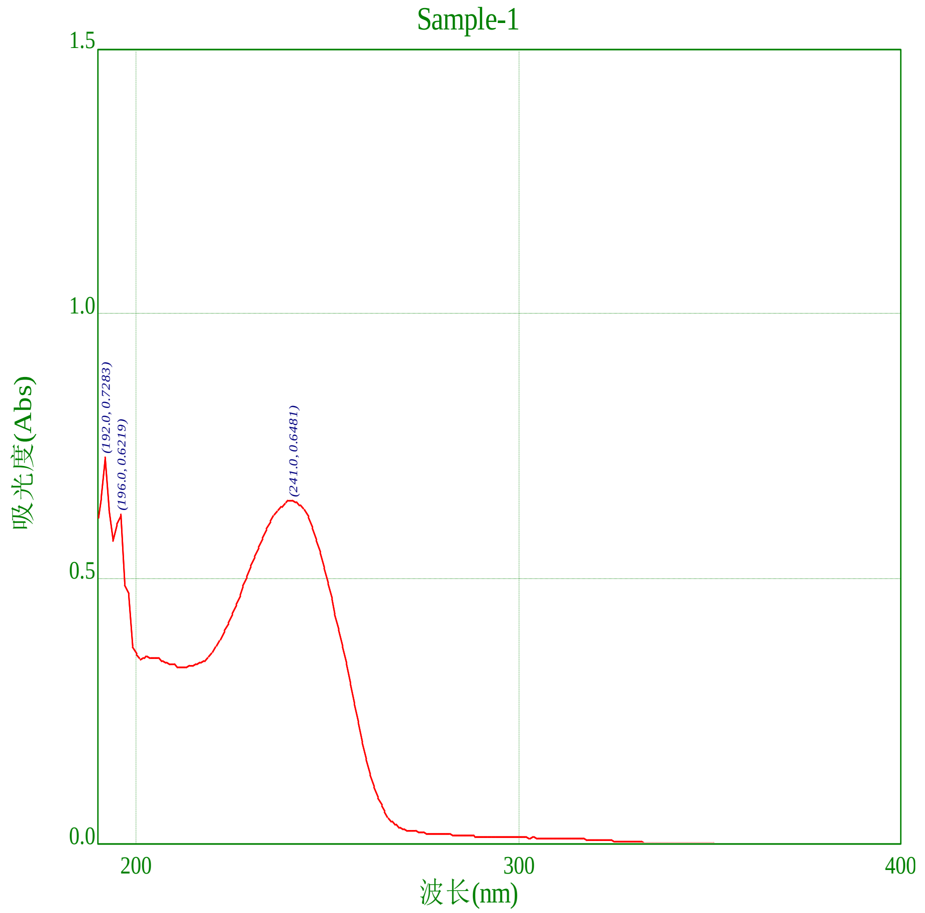

S14. IR spectrum of eutypelleudesmane A (**1**) (KBr).

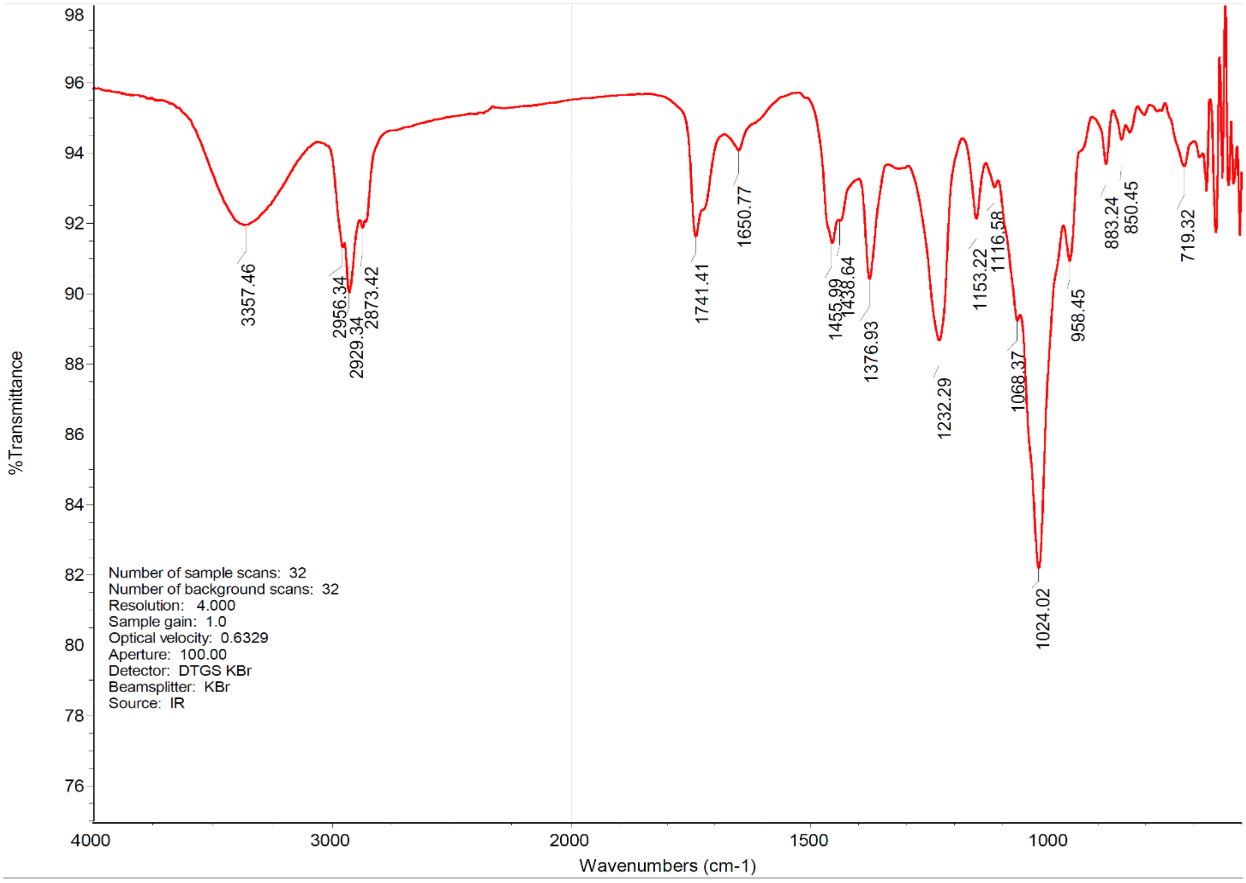

S25. ^{1}H NMR spectrum of cytosporin X (**2**) in $CDCl_3$.

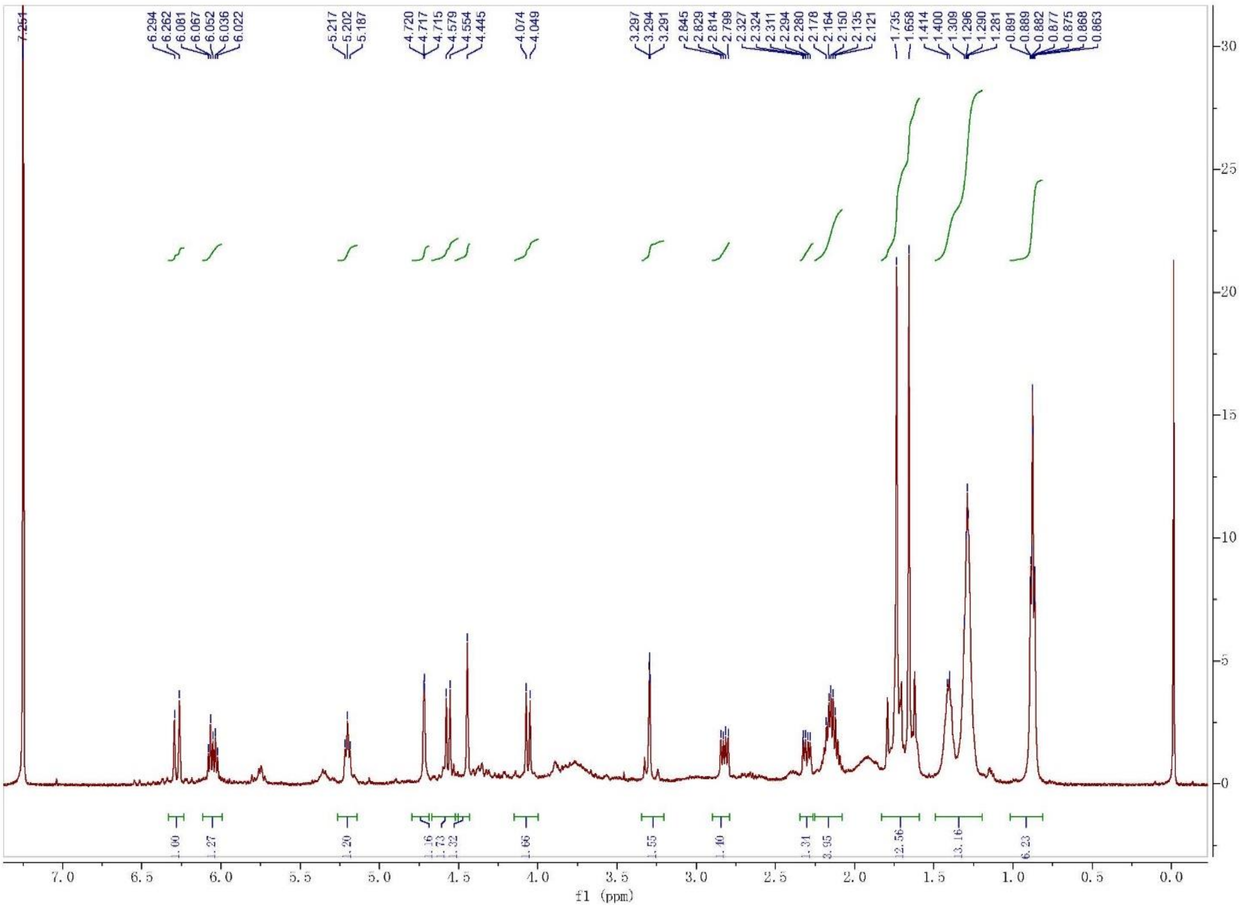

S36. ^{1}H NMR spectrum of cytosporin X (**2**) in DMSO-d_6.

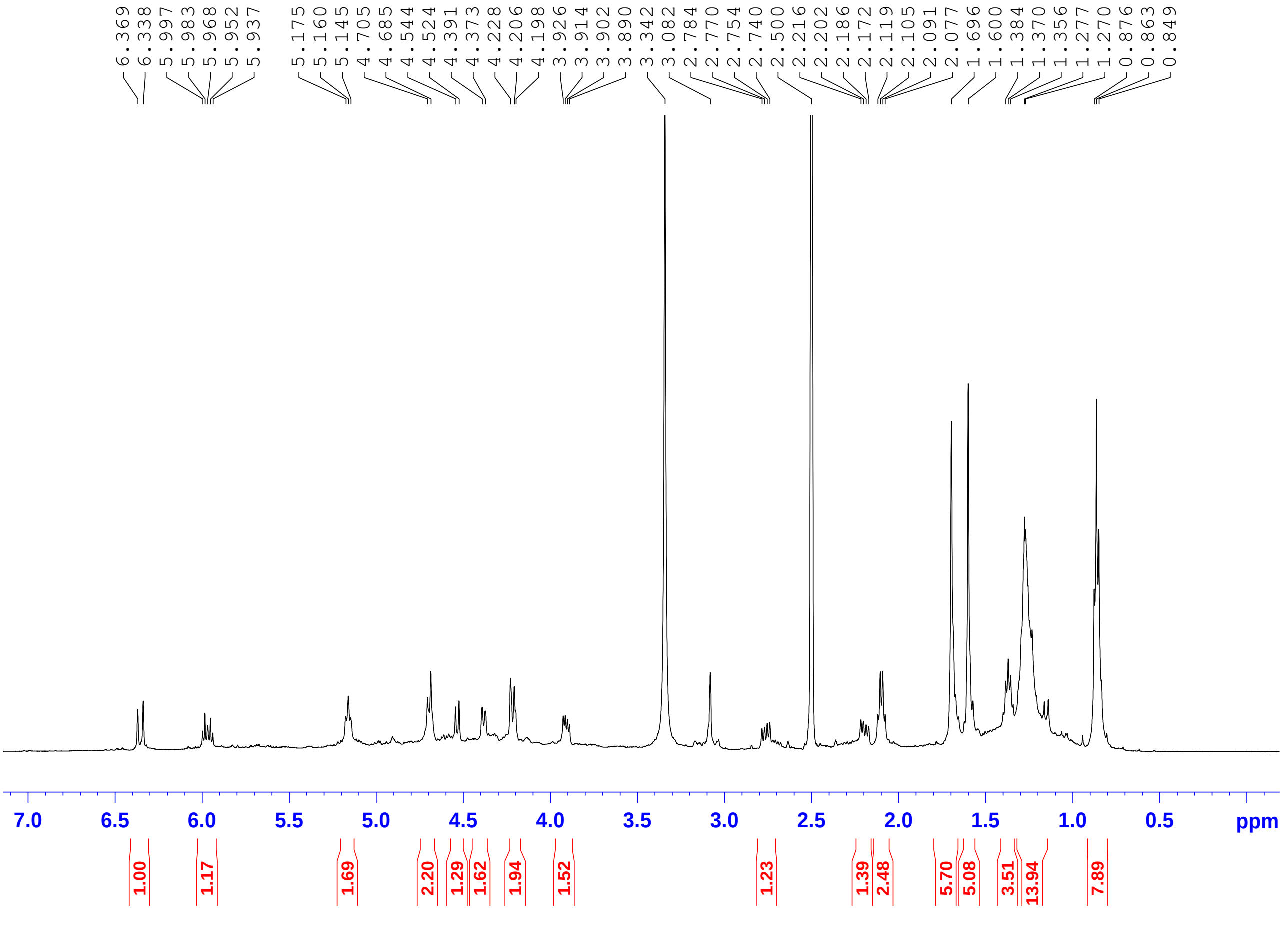

S47. ^{13}C NMR spectrum of cytosporin X (**2**) in $CDCl_3$.

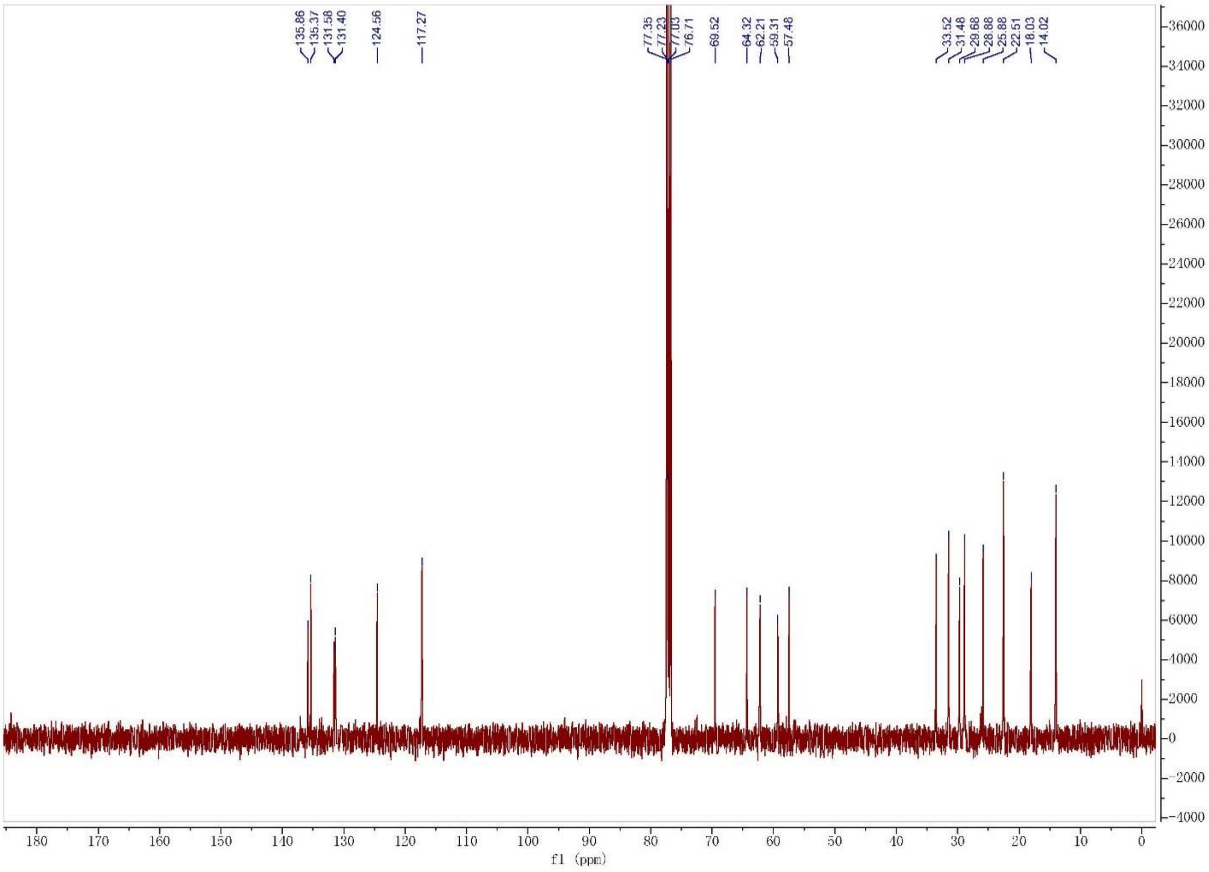

S58. DEPT135 spectrum of cytosporin X (**2**) in $CDCl_3$.

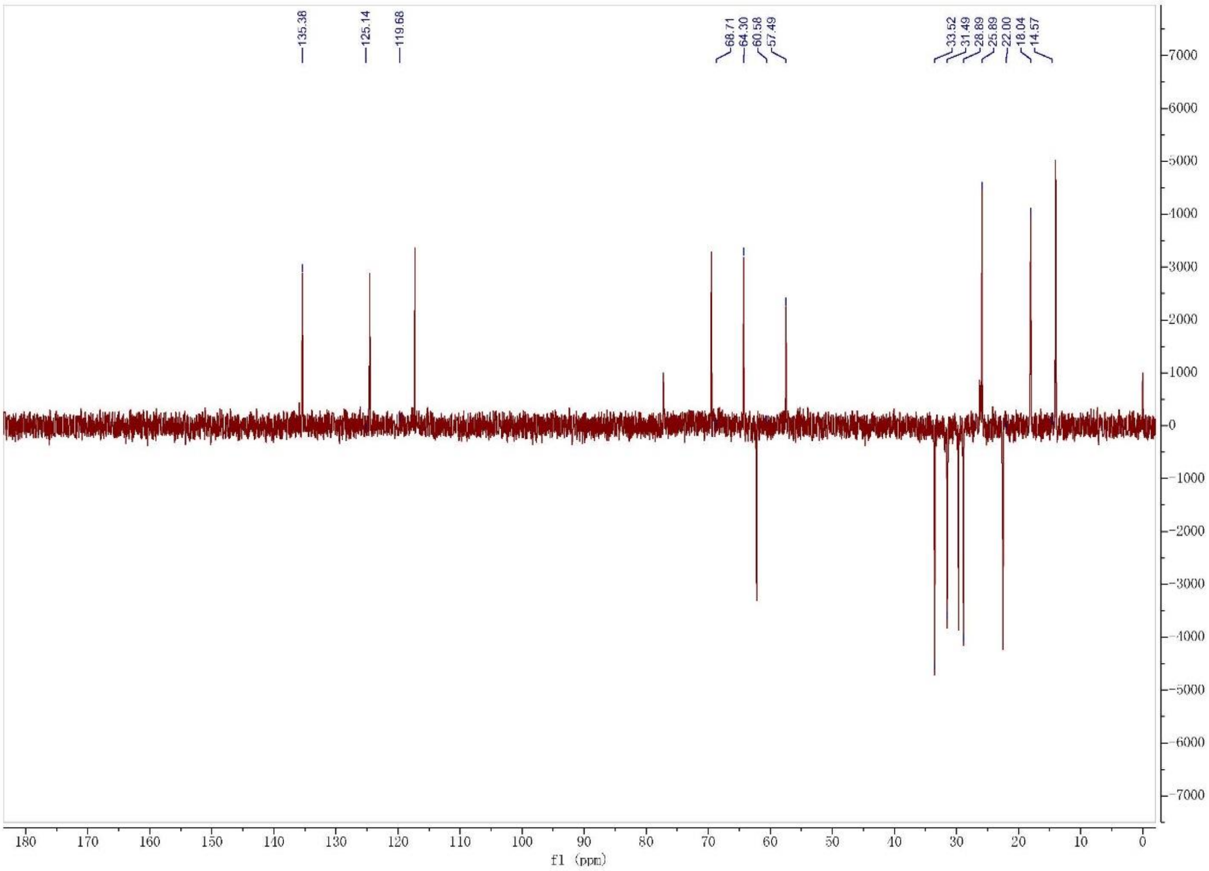

S69. HSQC spectrum of cytosporin X (**2**) in $CDCl_3$.

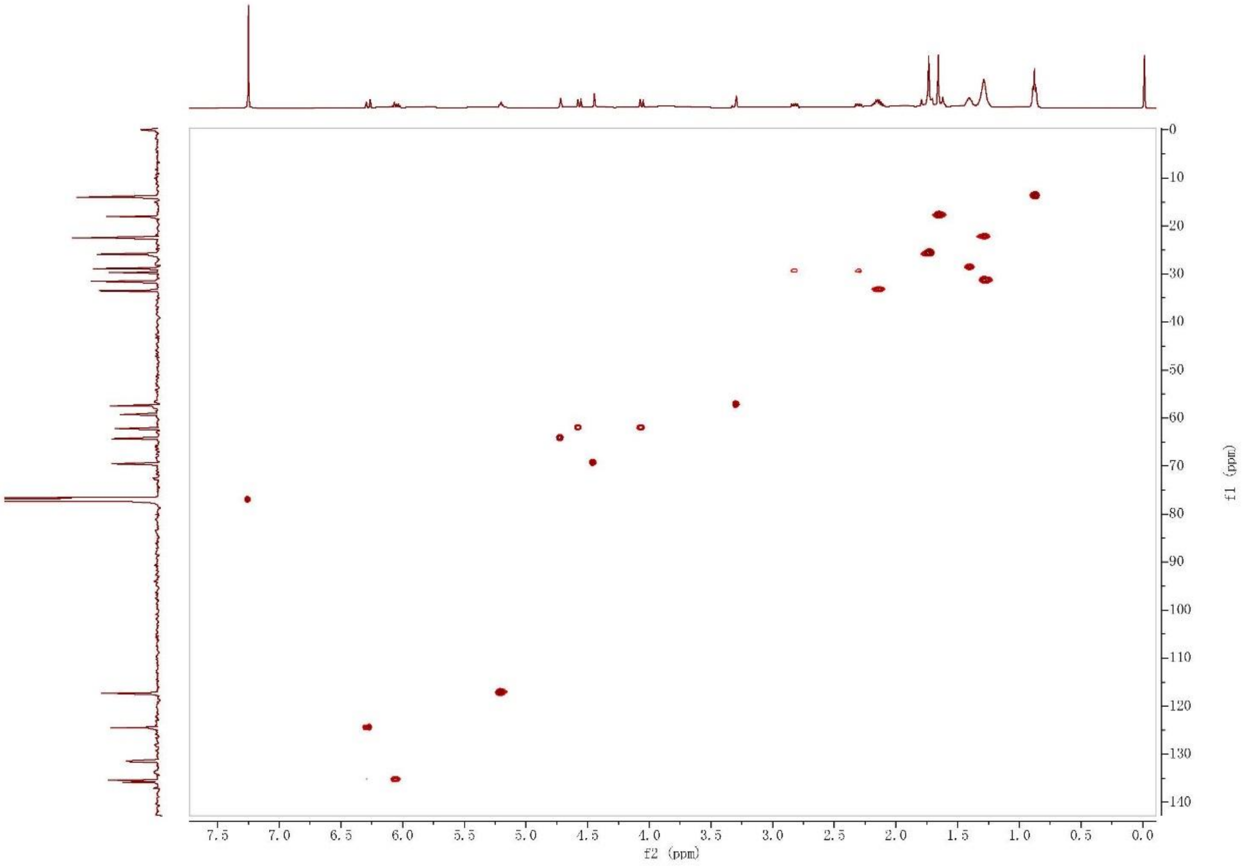

S20. COSY spectrum of cytosporin X (**2**) in $CDCl_3$.

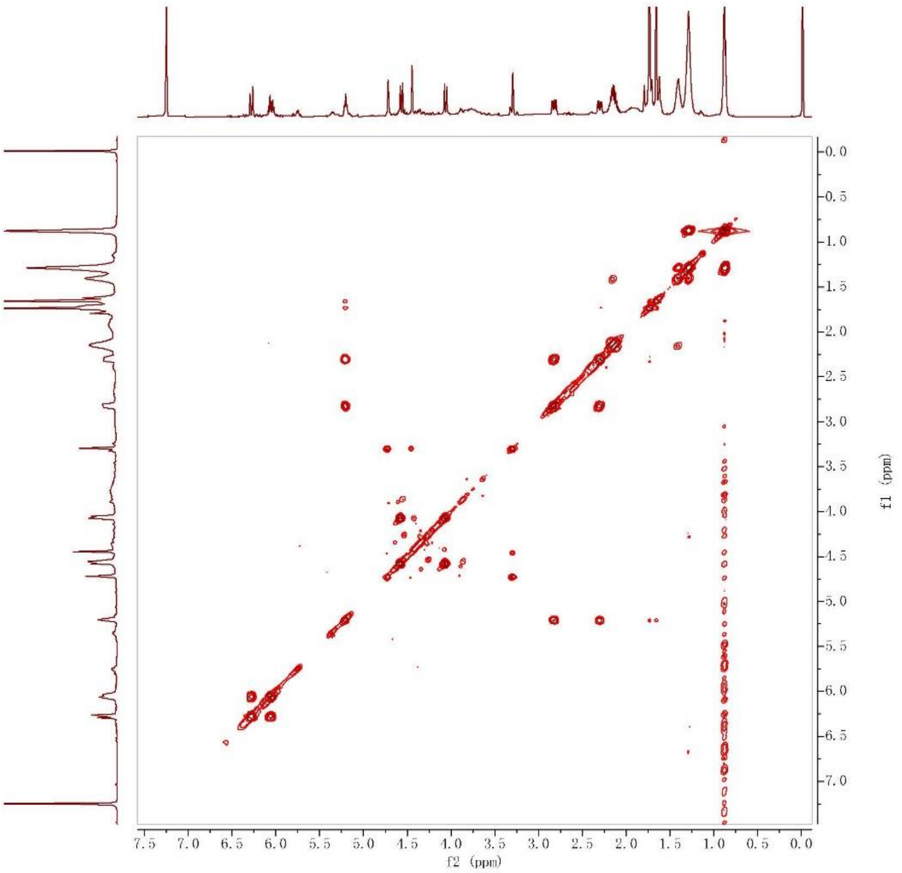

S21. HMBC spectrum of cytosporin X (**2**) in $CDCl_3$.

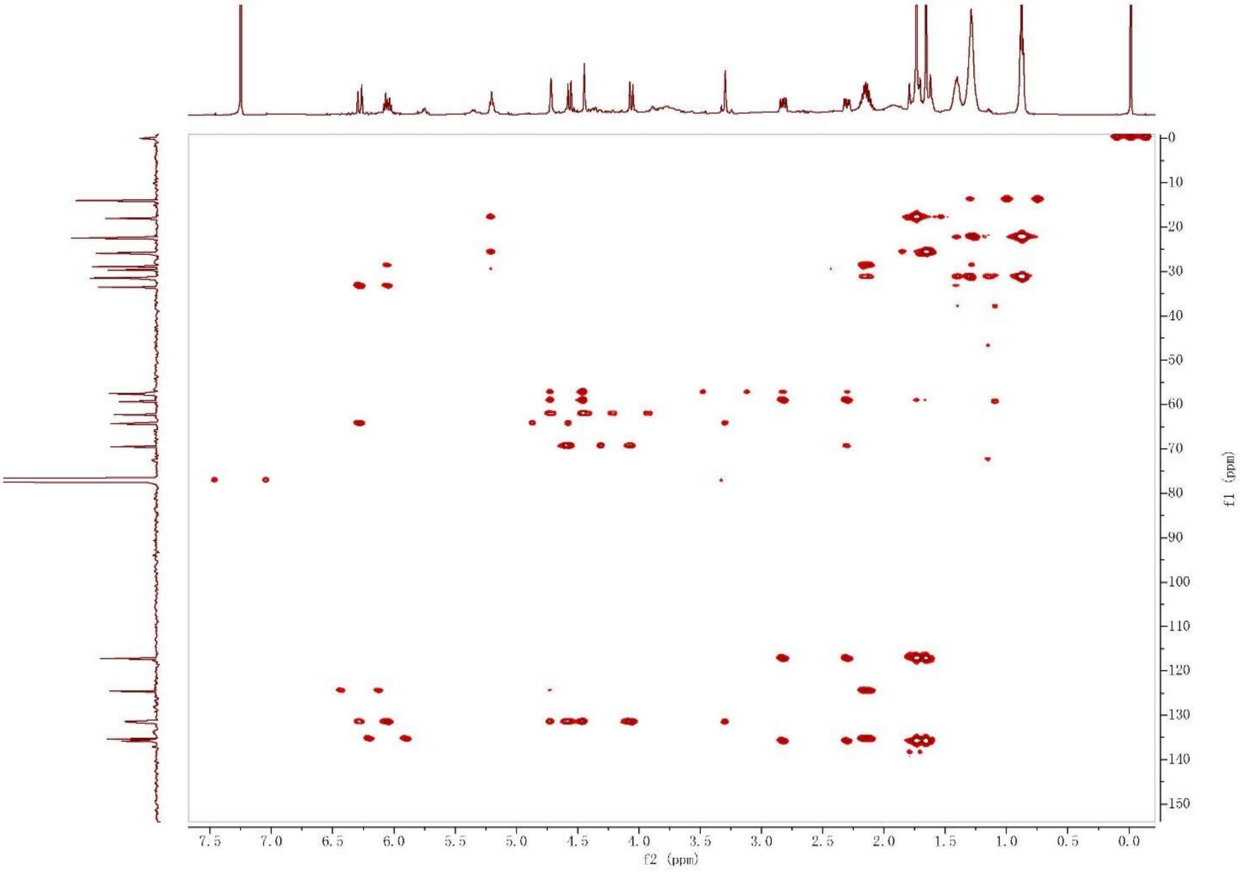

S22. NOESY spectrum of cytosporin X (**2**) in $CDCl_3$.

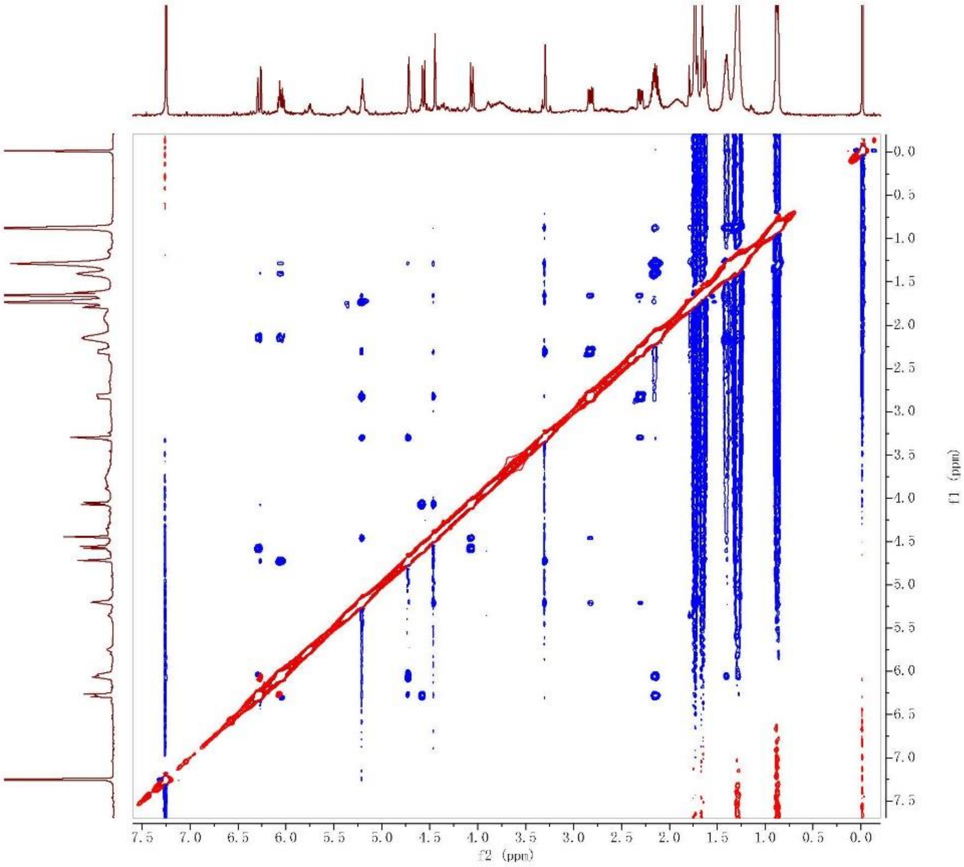

S23. NOESY spectrum of cytosporin X (**2**) in DMSO-d_6.

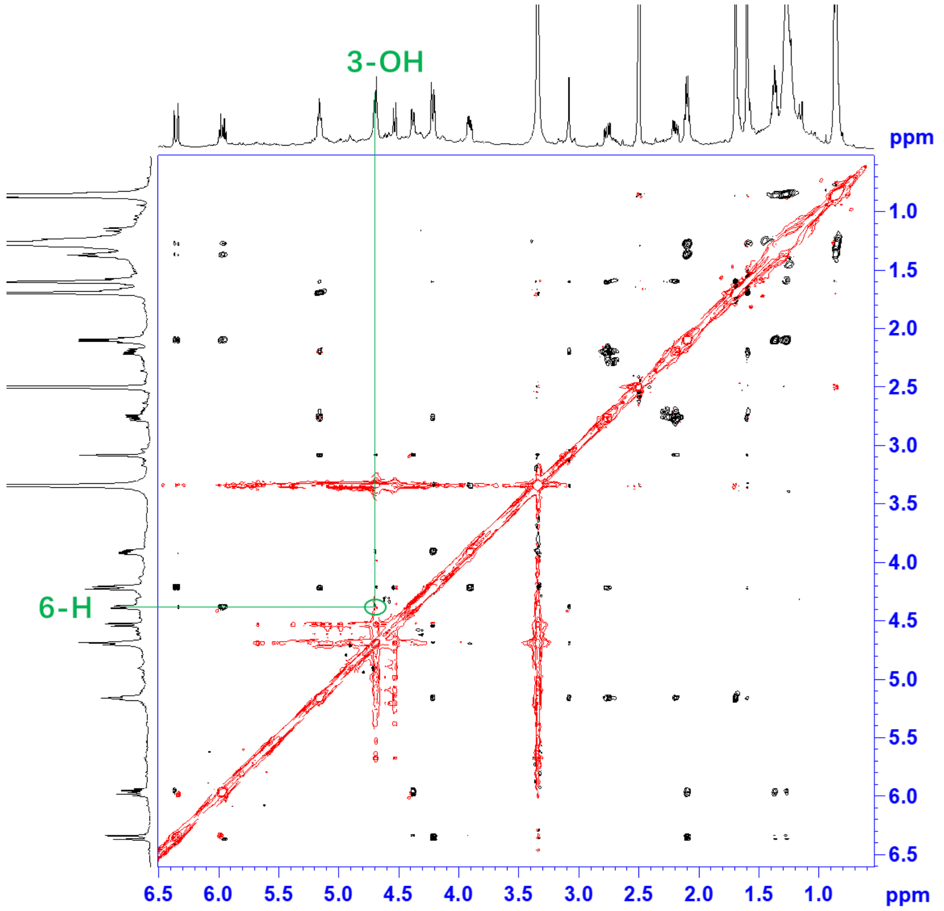

S24. HRESIMS of cytosporin X (**2**).

Qualitative Analysis Report

**Data Filename**	W-45-NEG.d	**Sample Name**	
**Sample Type**	Sample	**Position**	P1-E4
**Instrument Name**	Instrument 1	**User Name**	
**Acq Method**	SERUM-NEG-15MIN.m	**Acquired Time**	
**IRM Calibration Status**	Success	**DA Method**	E.m
**Comment**			
**Sample Group**	**Info.**		

User Spectra

Fragmentor Voltage	Collision Energy	Ionization Mode
120	0	ESI

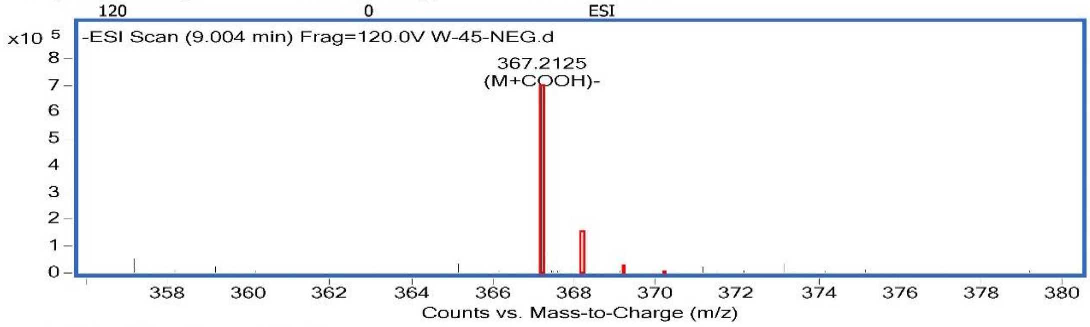

Formula Calculator Element Limits

Element	Min	Max
C	0	50
H	0	400
O	0	20

Formula Calculator Results

Formula	Best	Mass	Tgt Mass	Diff (ppm)	Ion Species	Score
C20 H31 O6	TRUE	367.2125	367.2121	1.1	C20 H31 O6	97.64

--- End Of Report ---

S25. UV spectrum of cytosporin X (**2**) in MeOH.

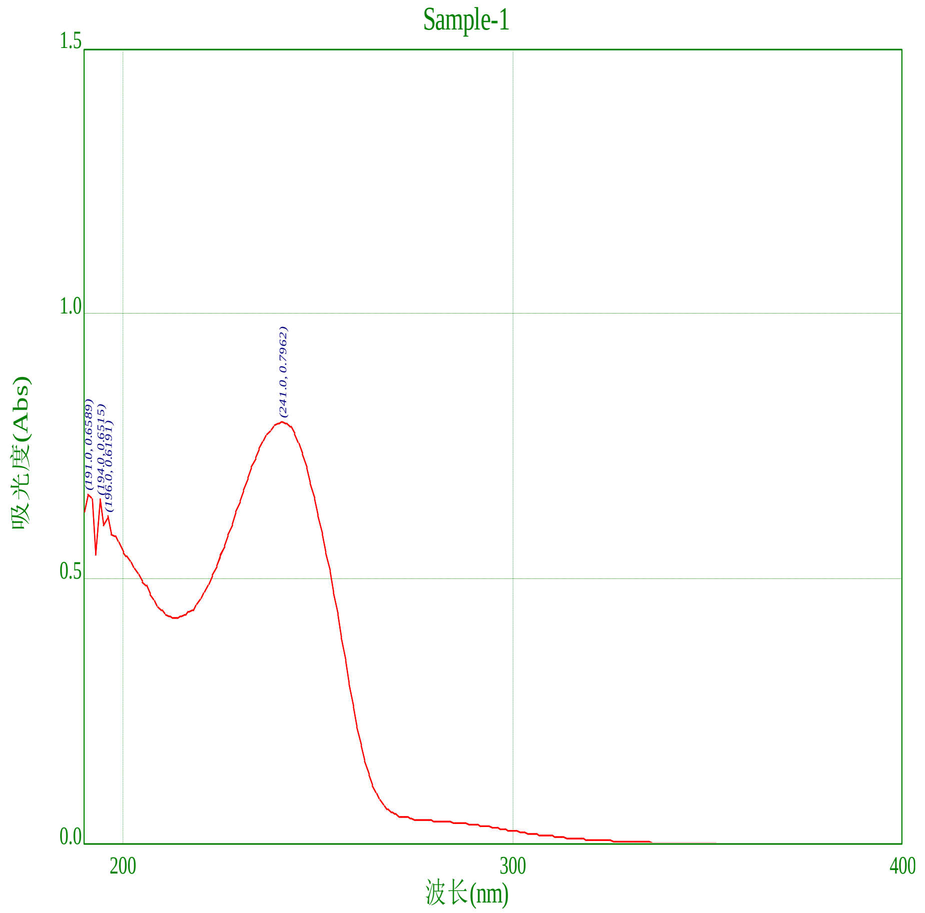

S26 IR spectrum of cytosporin X (**2**) (KBr).

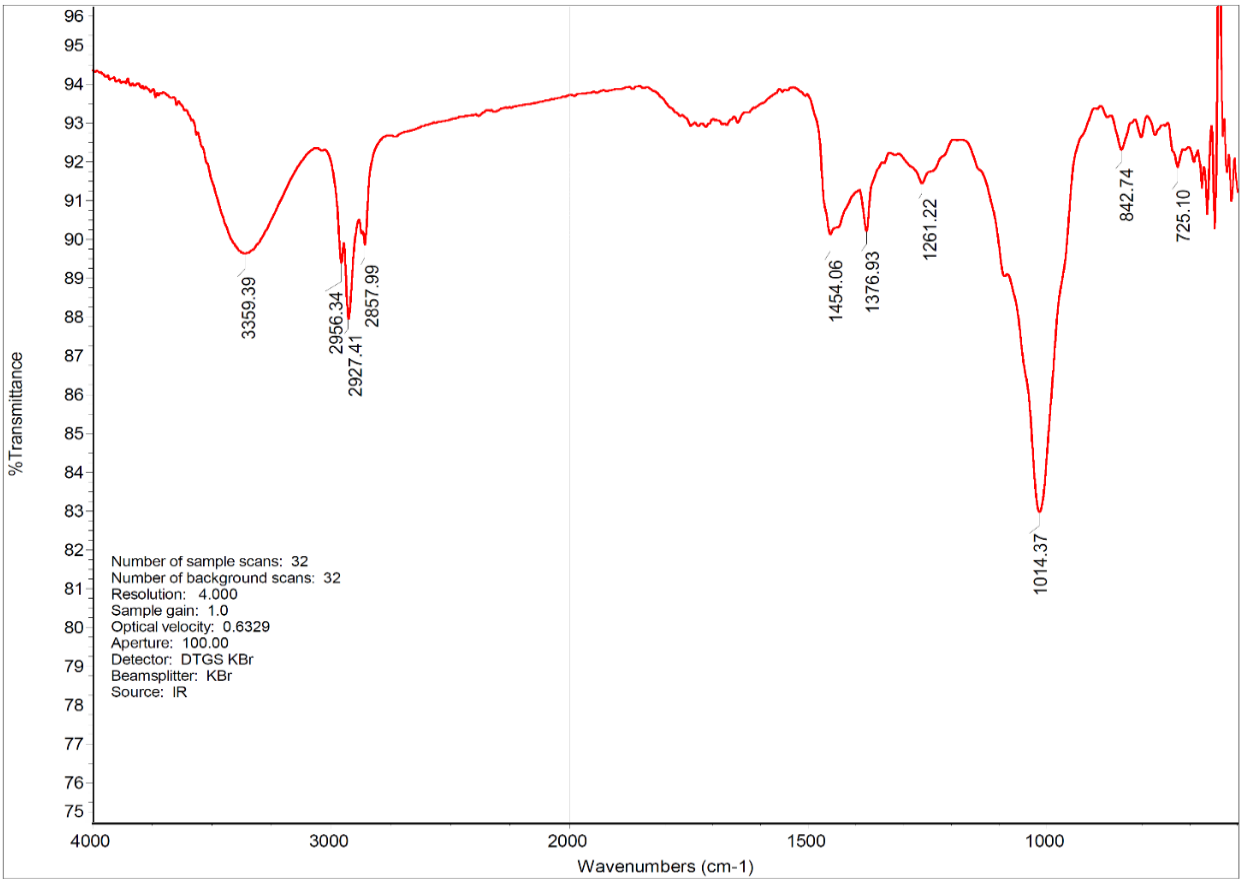

S27. ^{1}H NMR spectrum of cytosporin Y (**3**) in $CDCl_3$.

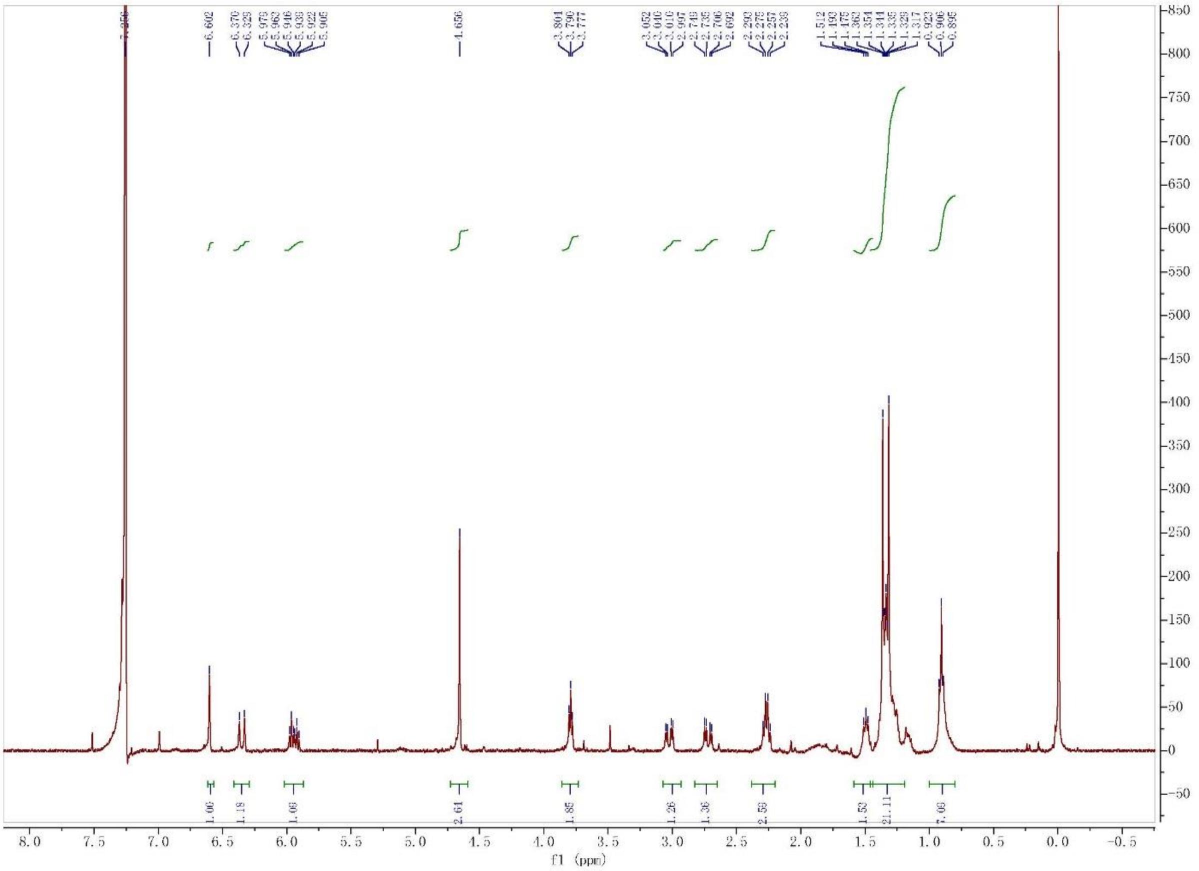

S28. ^{13}C NMR spectrum of cytosporin Y (**3**) in $CDCl_3$.

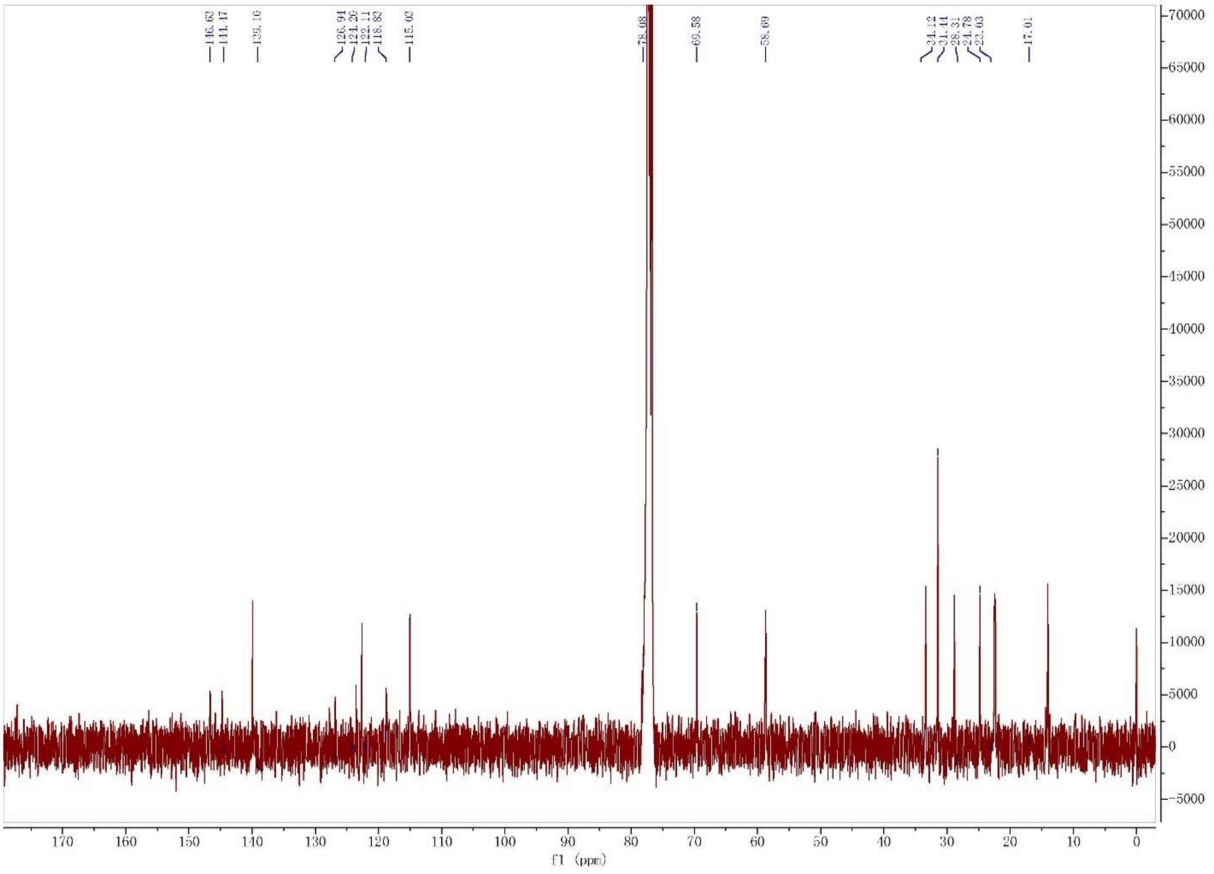

S29. DEPT135 spectrum of cytosporin Y (**3**) in $CDCl_3$.

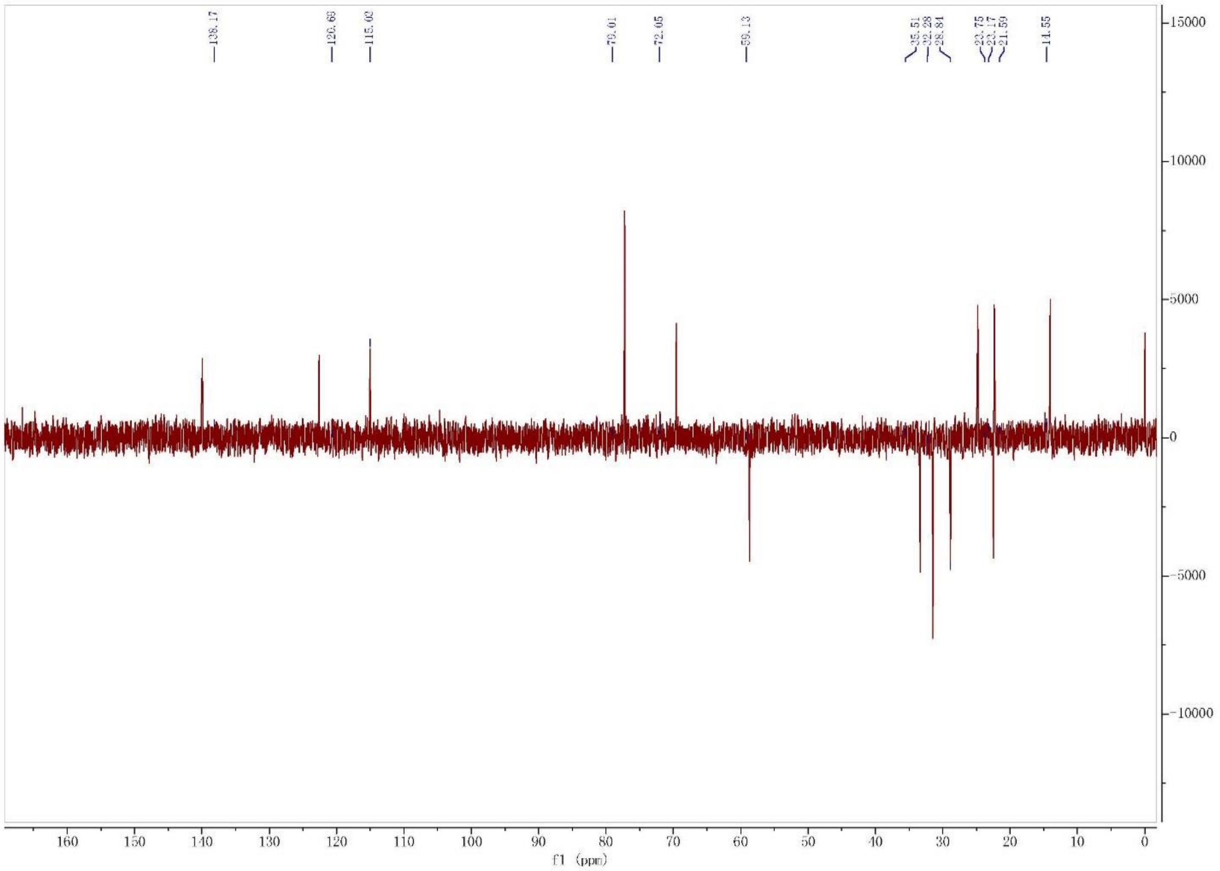

S30. HSQC spectrum of cytosporin Y (**3**) in $CDCl_3$.

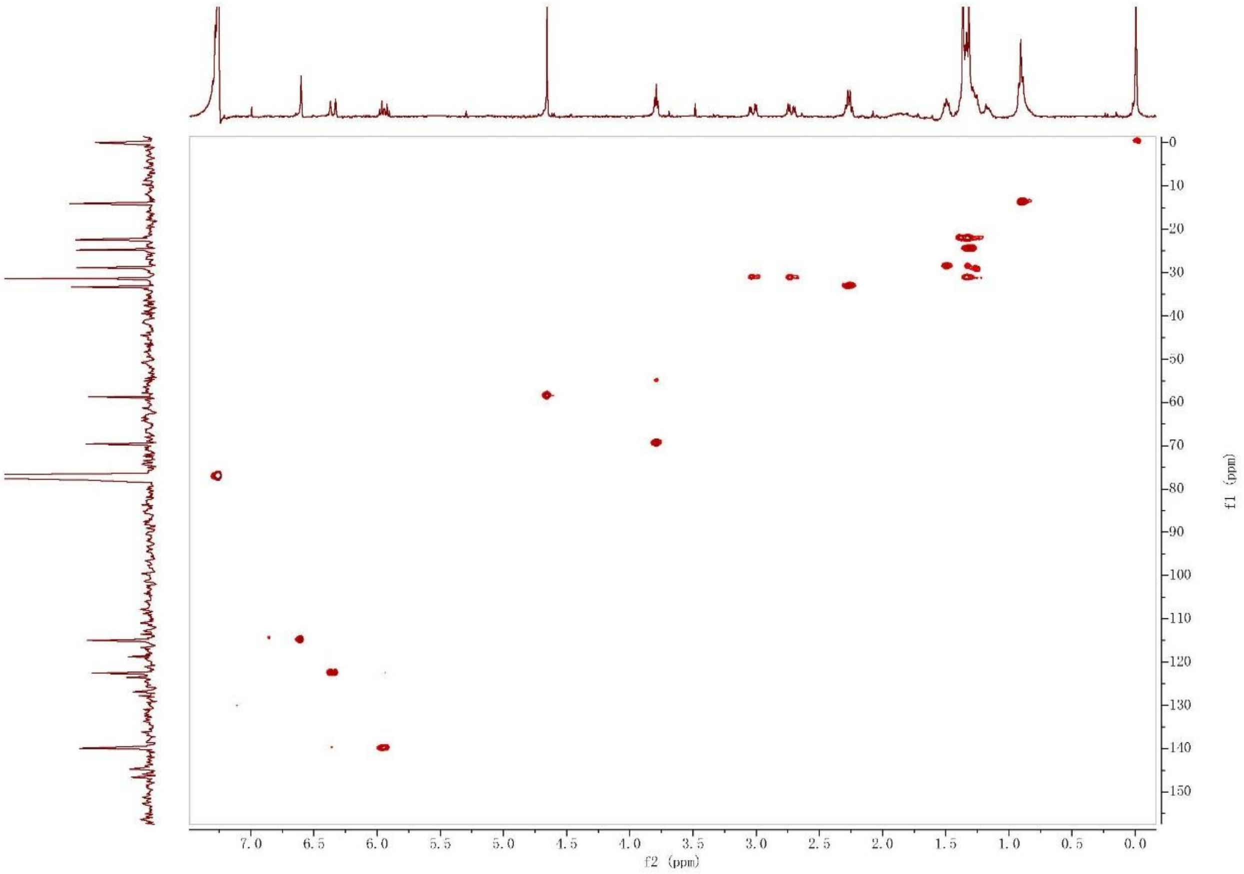

S31. COSY spectrum of cytosporin Y (**3**) in $CDCl_3$.

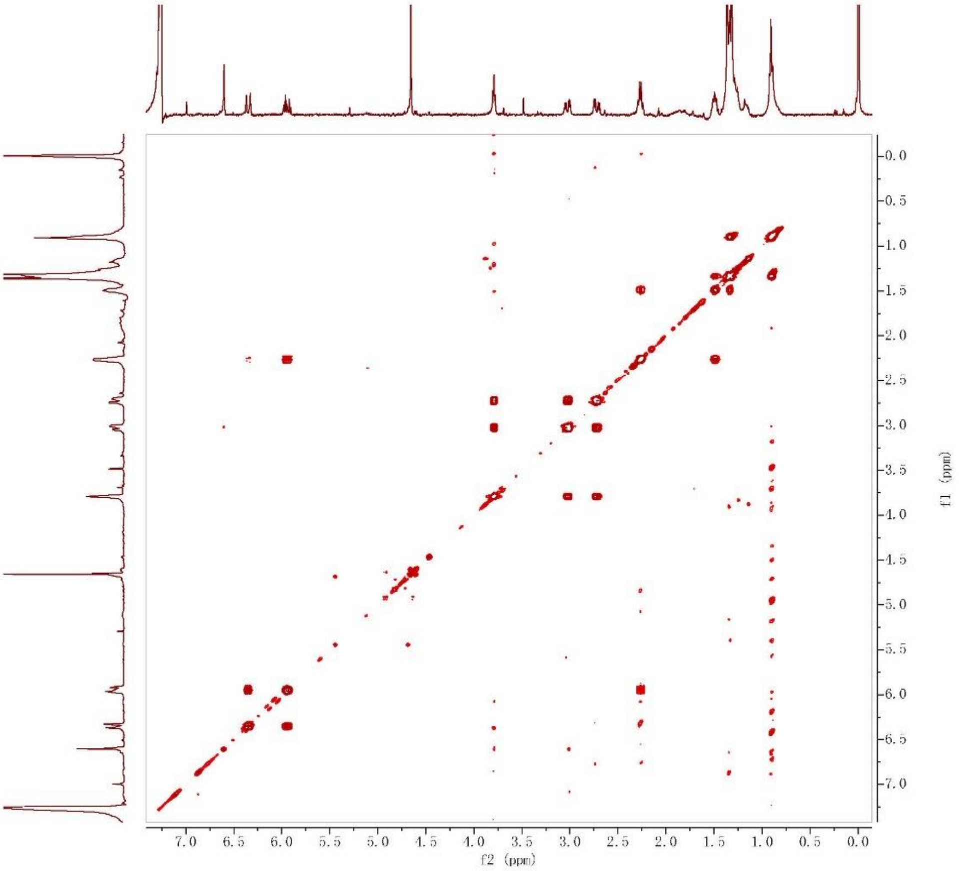

S32. HMBC spectrum of cytosporin Y (**3**) in $CDCl_3$.

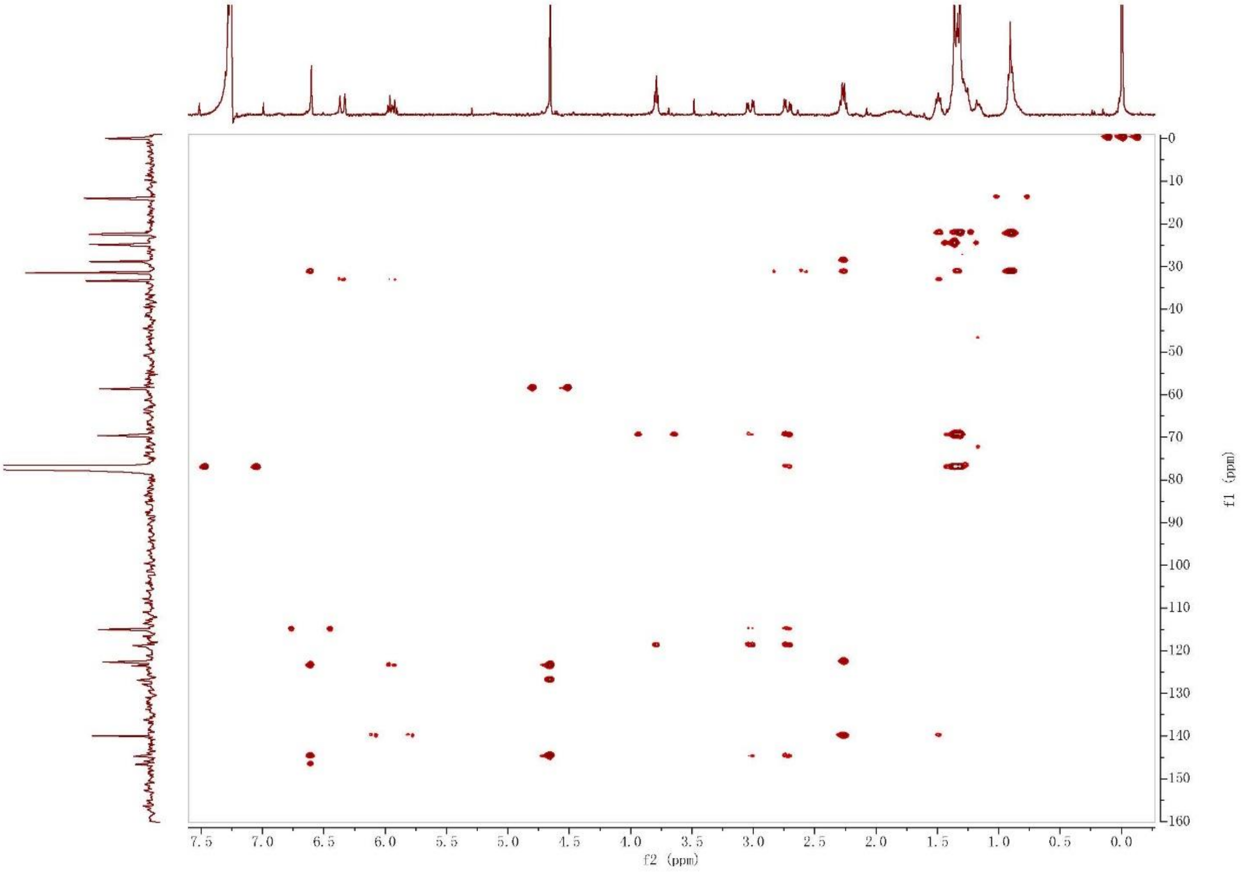

S33. NOESY spectrum of cytosporin Y (**3**) in $CDCl_3$.

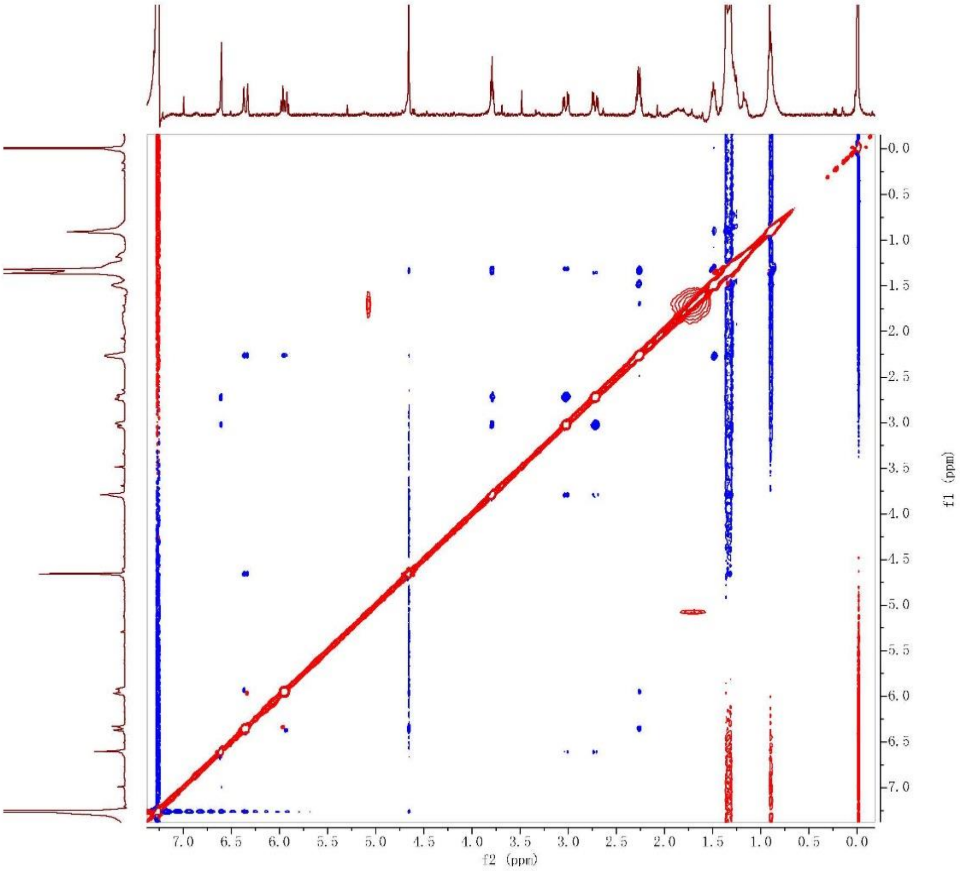

S34. HRESIMS of cytosporin Y (**3**).

Qualitative Analysis Report

**Data Filename**	W-55-NEG.d	**Sample Name**	
**Sample Type**	Sample	**Position**	Vial 9
**Instrument Name**	Instrument 1	**User Name**	
**Acq Method**	SERUM-NEG-15MIN.m	**Acquired Time**	
**IRM Calibration Status**		**DA Method**	E.m
**Sample Group** **Info.**			

User Spectra

**Fragmentor Voltage**	**Collision Energy**	**Ionization Mode**
120	0	ESI

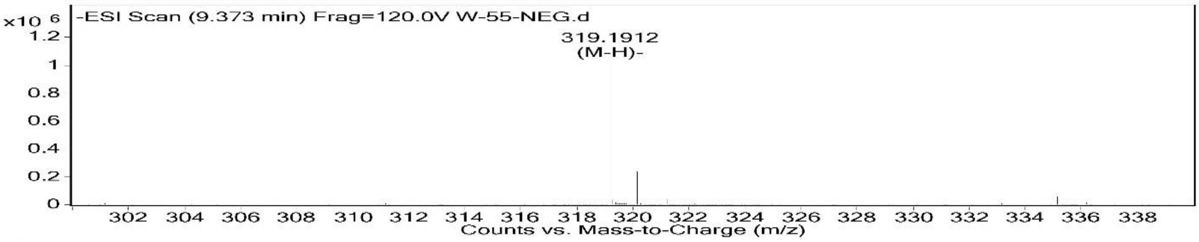

Peak List

m/z	z	Abund	Formula	Ion
319.1912	1	1248103	C19 H27 O4	(M-H)-
320.1954	1	235904.6	C19 H27 O4	(M-H)-
469.208	1	624315.1		
470.2118	1	152830.9		
743.4017		143111.5		

Formula Calculator Element Limits

Element	Min	Max
C	0	200
H	0	400
O	0	17

Formula Calculator Results

Formula	Best	Mass	Tgt Mass	Diff (ppm)	Ion Species	Score
C19 H27O4	TRUE	319.1912	319.1909	0.94	C19 H27 O4	98.02

--- End Of Report ---

S35. UV spectrum of cytosporin Y (**3**) in MeOH.

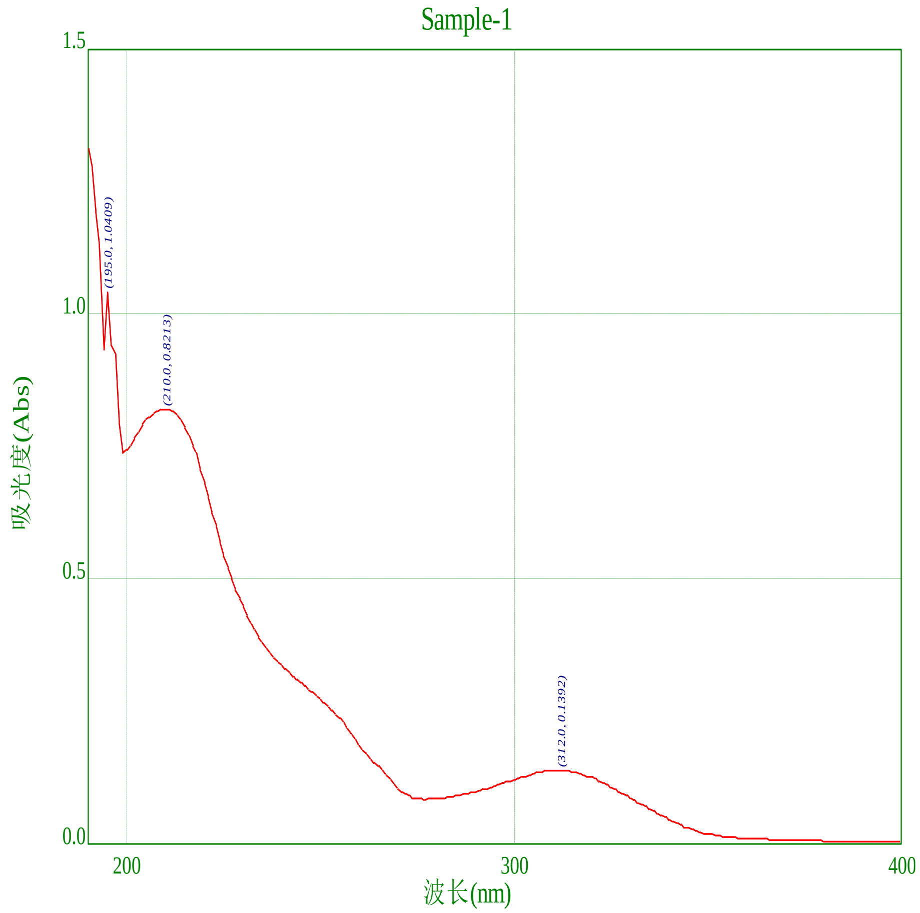

S36. IR spectrum of cytosporin Y (**3**) (KBr).

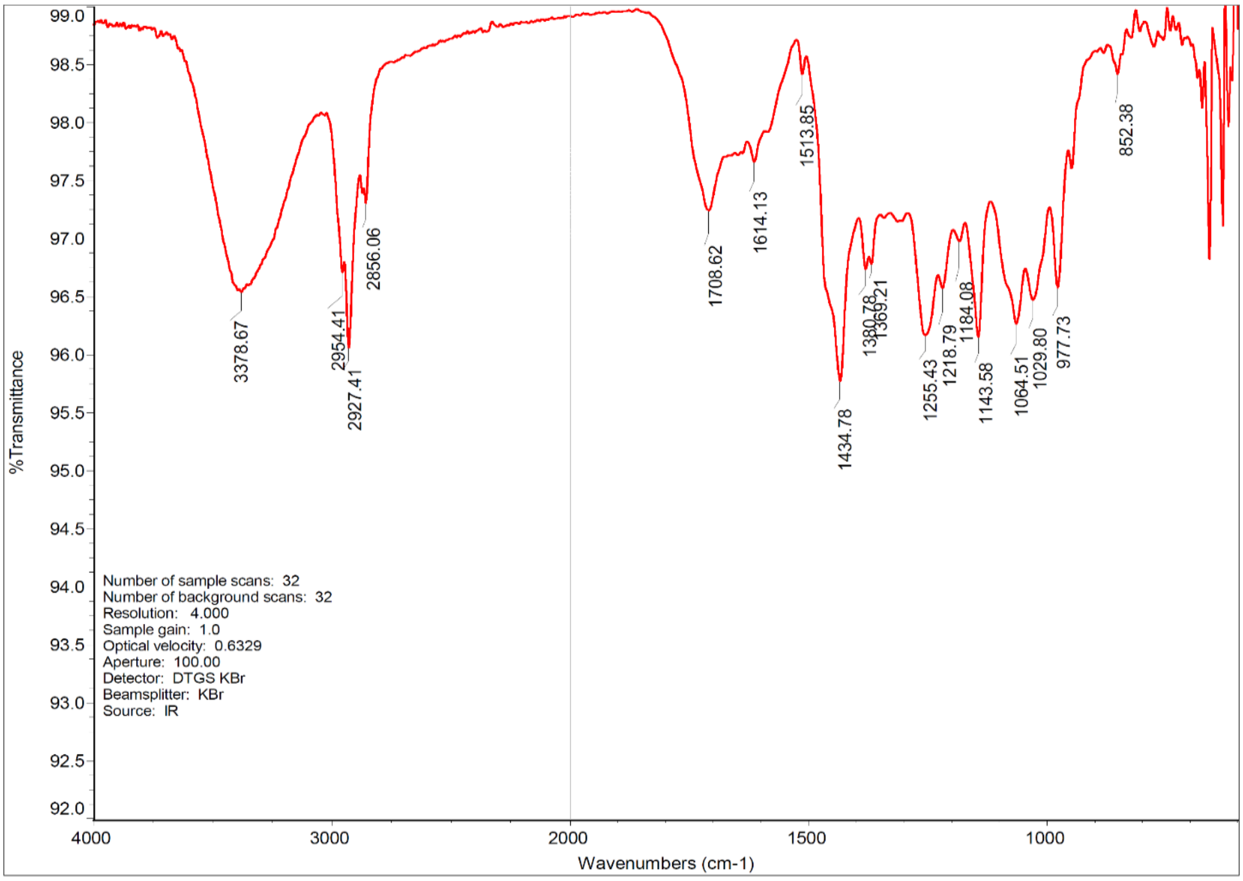

S37. ^{1}H NMR spectrum of cytosporin Y_1 (**4**) in MeOD-d_4.

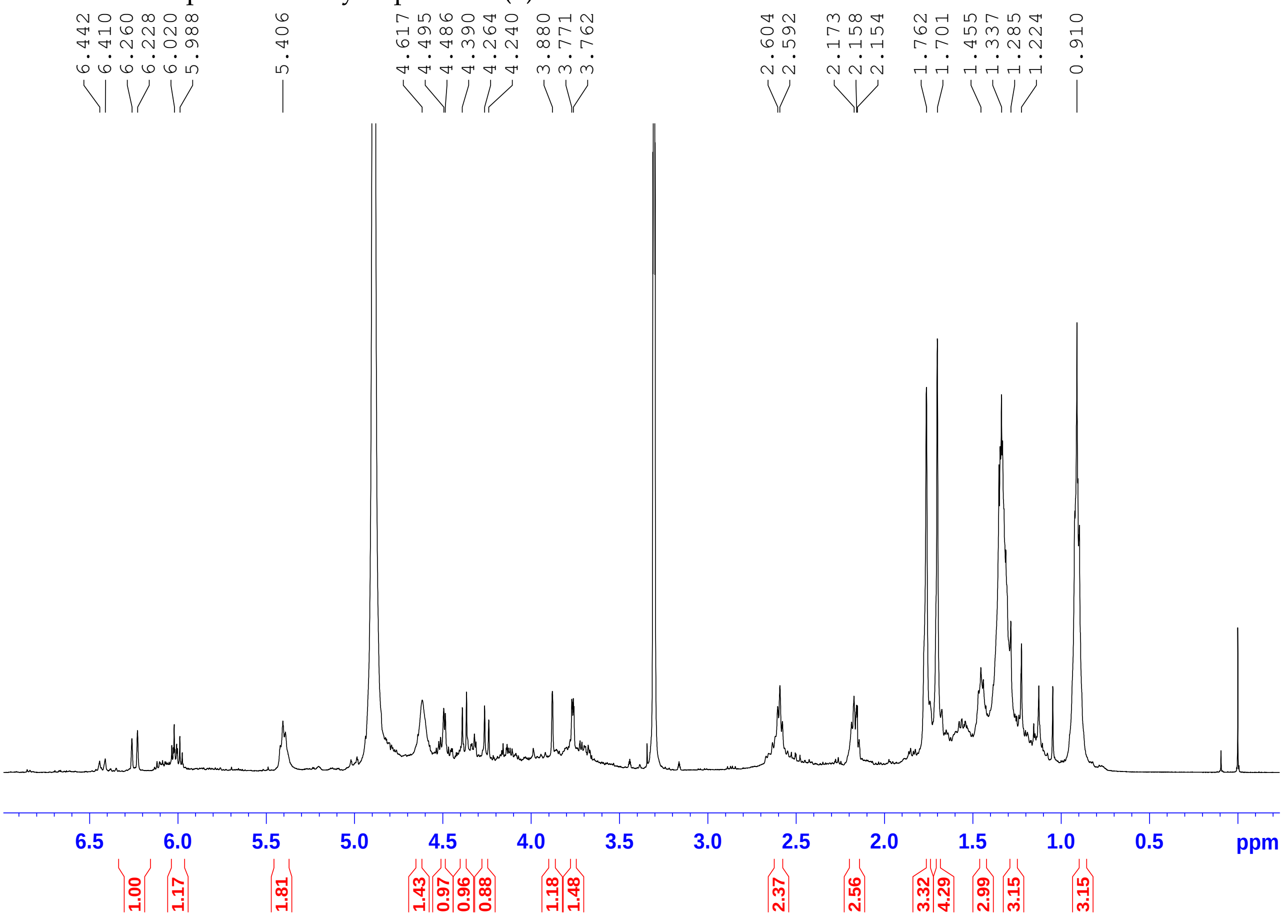

S38. ^{13}C NMR spectrum of cytosporin Y_1 (**4**) in MeOD-d_4.

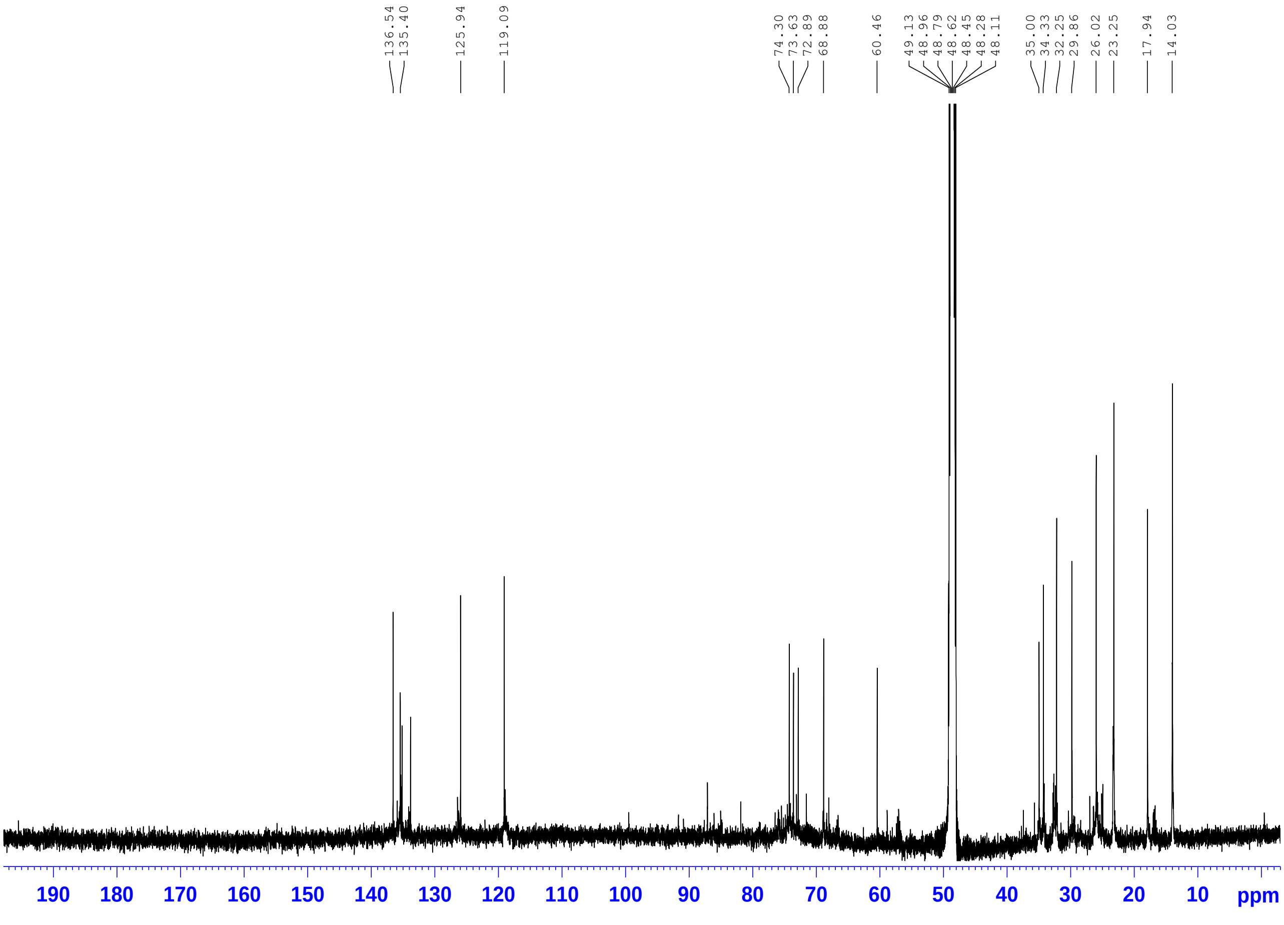

S39. DEPT135 spectrum of cytosporin Y_1 (**4**) in MeOD-d_4.

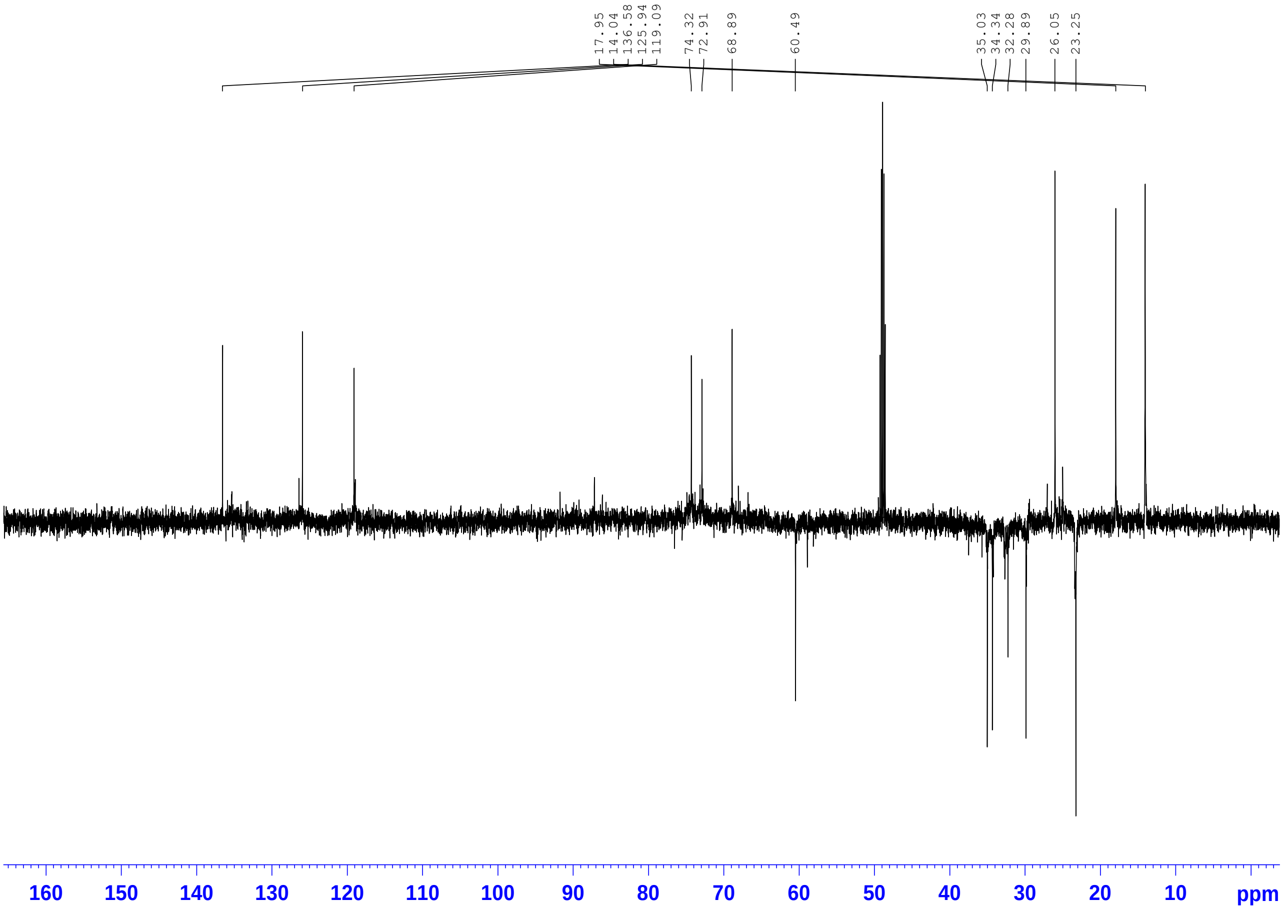

S40. HSQC spectrum of cytosporin Y_1 (**4**) in MeOD-d_4.

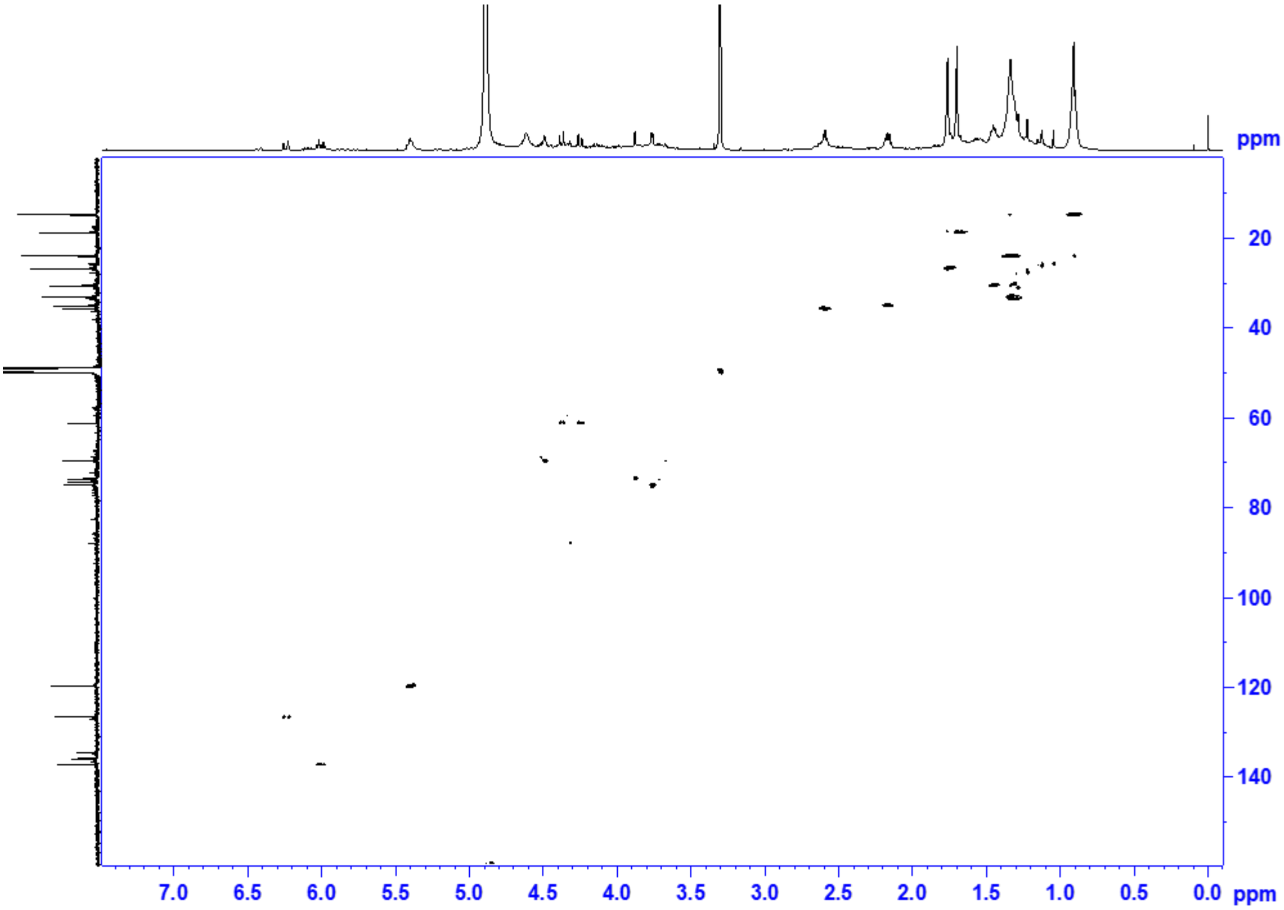

S41. COSY spectrum of cytosporin Y1 (**4**) in MeOD-*d*4.

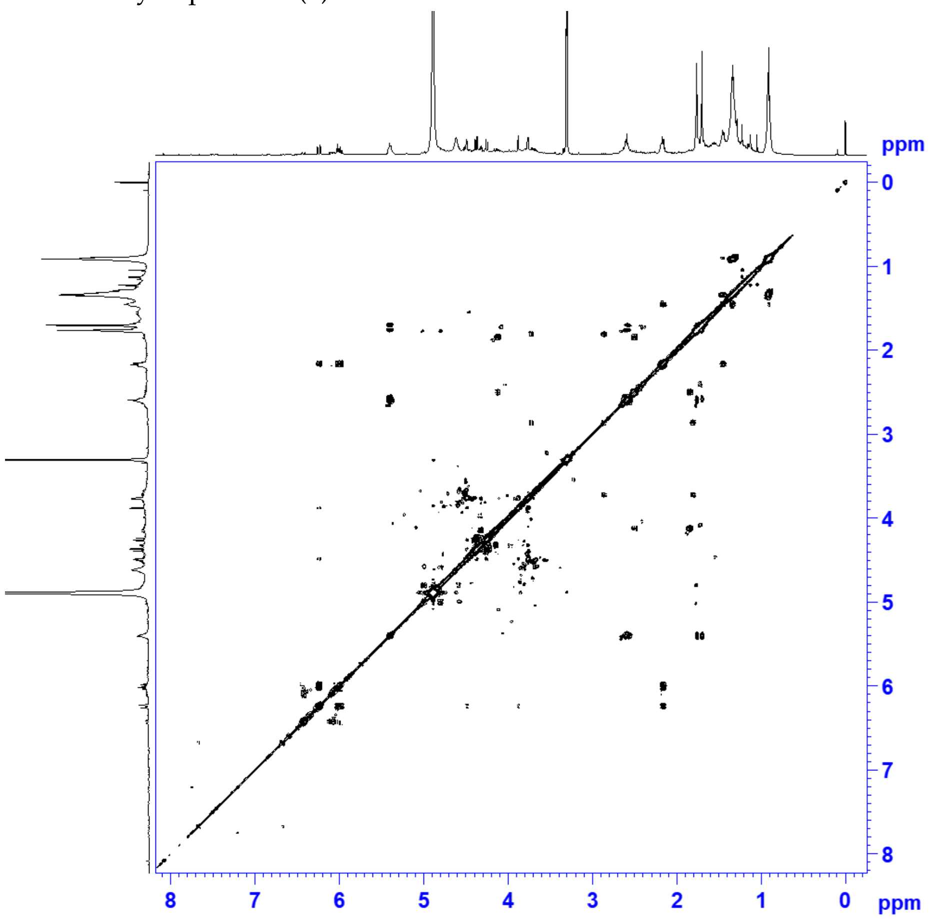

S42. HMBC spectrum of cytosporin Y1 (**4**) in MeOD-*d*4.

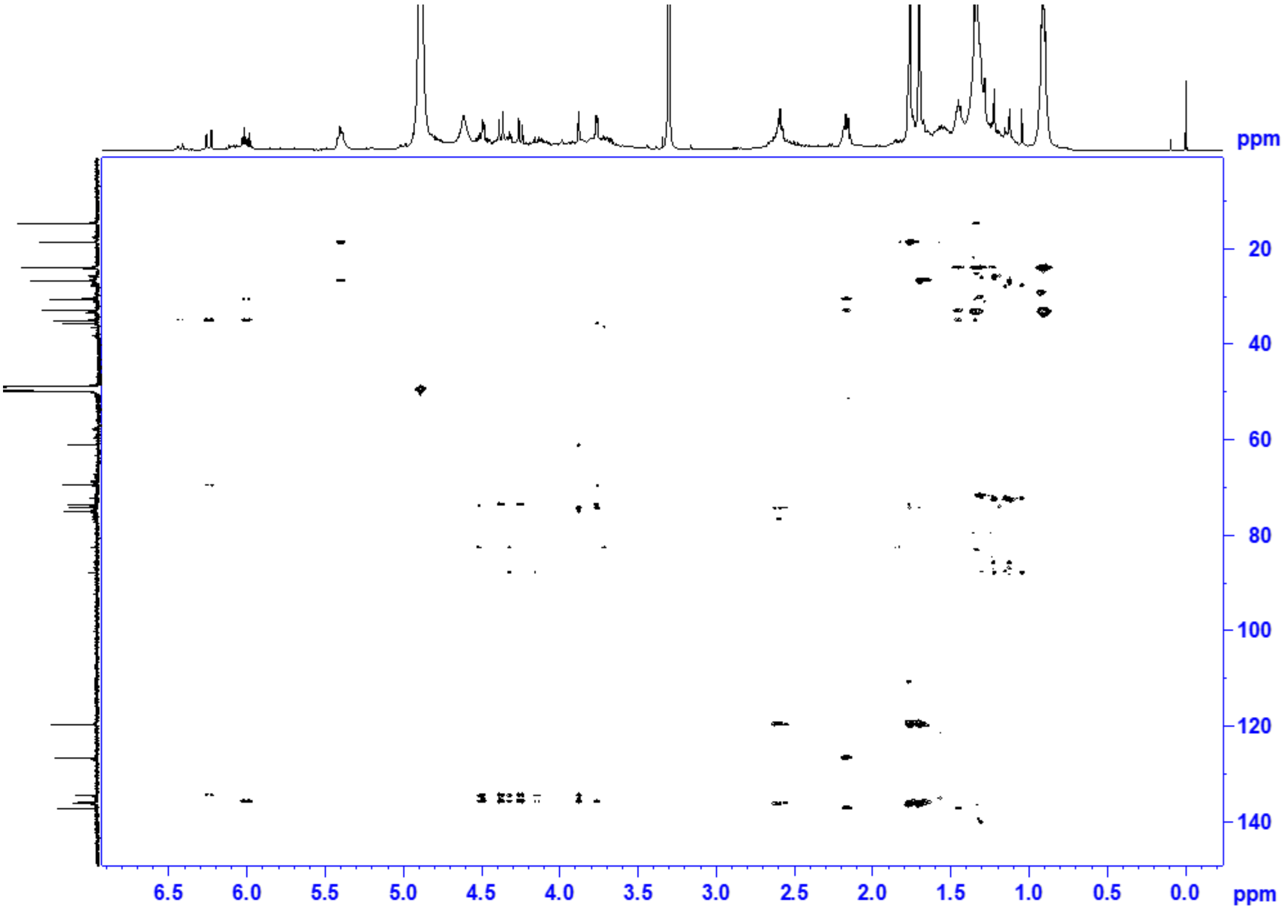

S43. NOESY spectrum of cytosporin Y$_1$ (**4**) in MeOD-d_4.

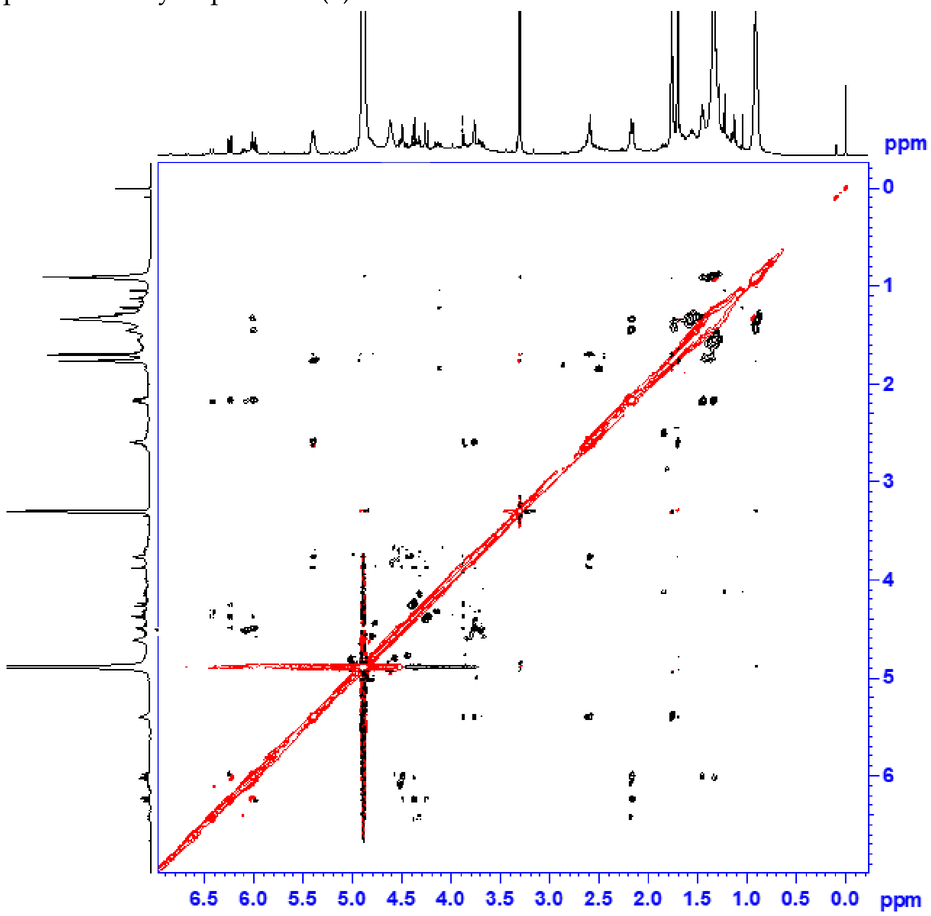

S44. HRESIMS of cytosporin Y$_1$ (**4**).

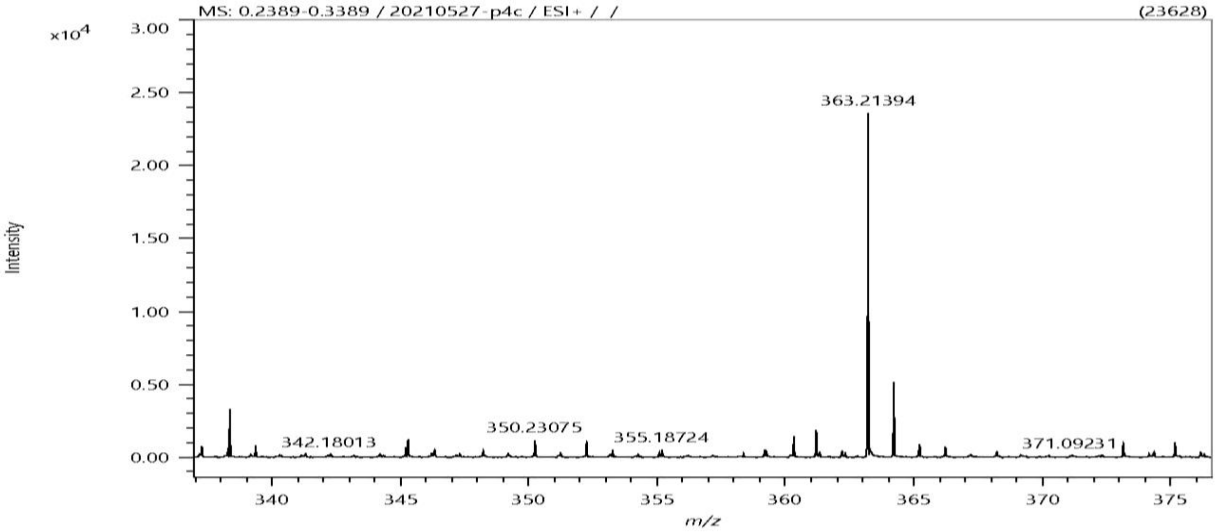

Elemental Composition

Parameters	
Tolerance:	±5.00 ppm
Electron:	Odd/Even
Charge:	+1
DBE:	-1.5 - 200.0

Elements Set 1:

Symbol	C	H	N	O	Na	S	Cl	Br
Min	0	0	0	0	1	0	0	0
Max	200	200	0	8	1	0	0	0

Symbol	F	Si
Min	0	0
Max	0	0

Results

Mass	Intensity	Intensity [%]	Formula	Calculated Mass	Mass Difference [mDa]	Mass Difference [ppm]	DBE
363.21394	23628.36	30.36	C19 H32 O5 Na	363.21420	-0.25	-0.70	3.5

S45. UV spectrum of cytosporin Y_1 (**4**) in MeOH.

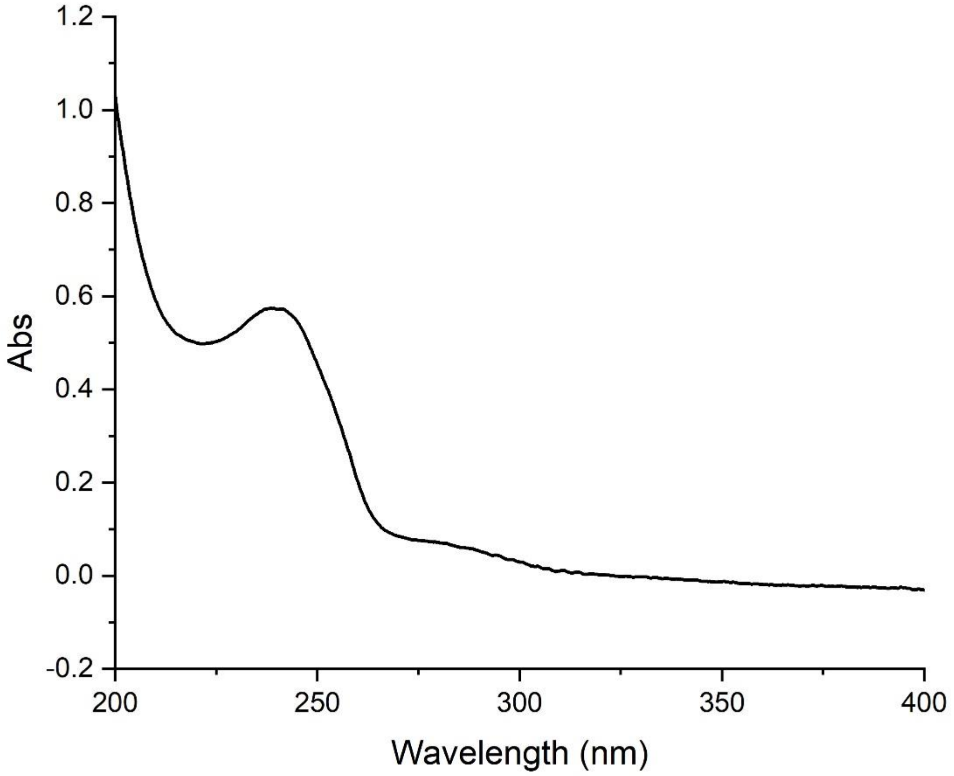

S46. IR spectrum of cytosporin Y_1 (**4**) (KBr).

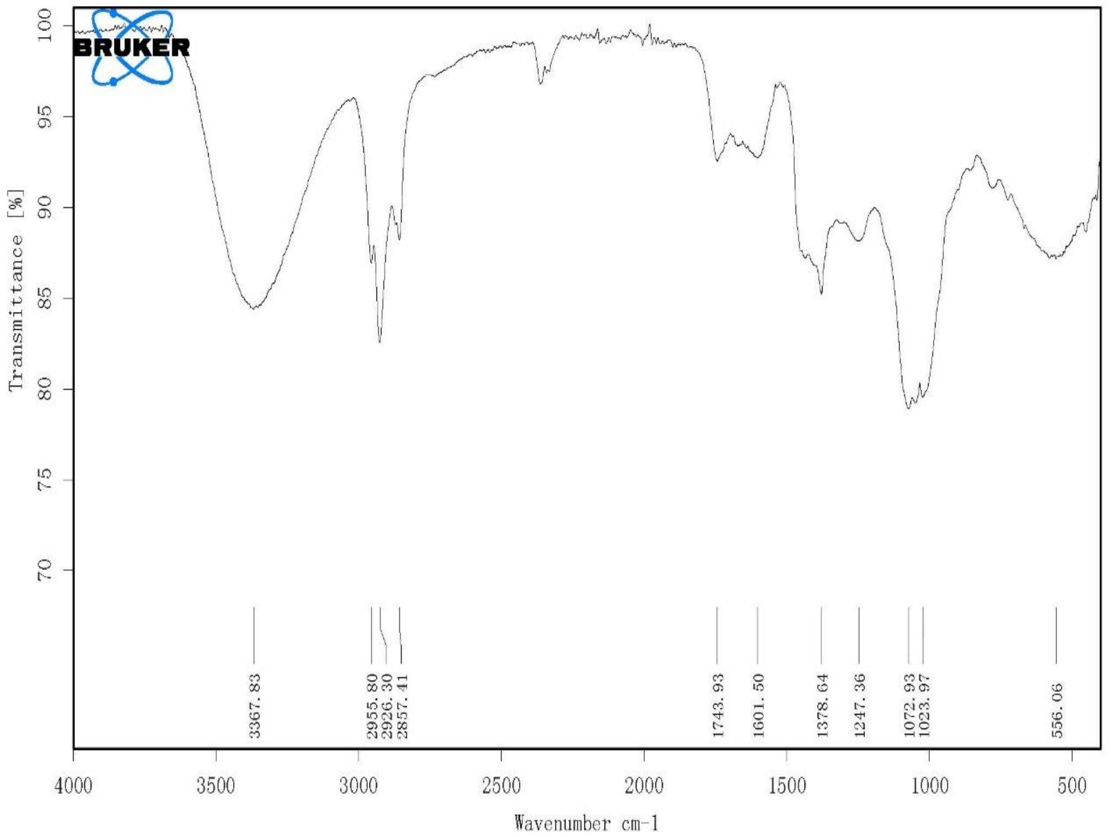

S47. ^{1}H NMR spectrum of cytosporin Y_2 (**5**) in MeOD-d_4.

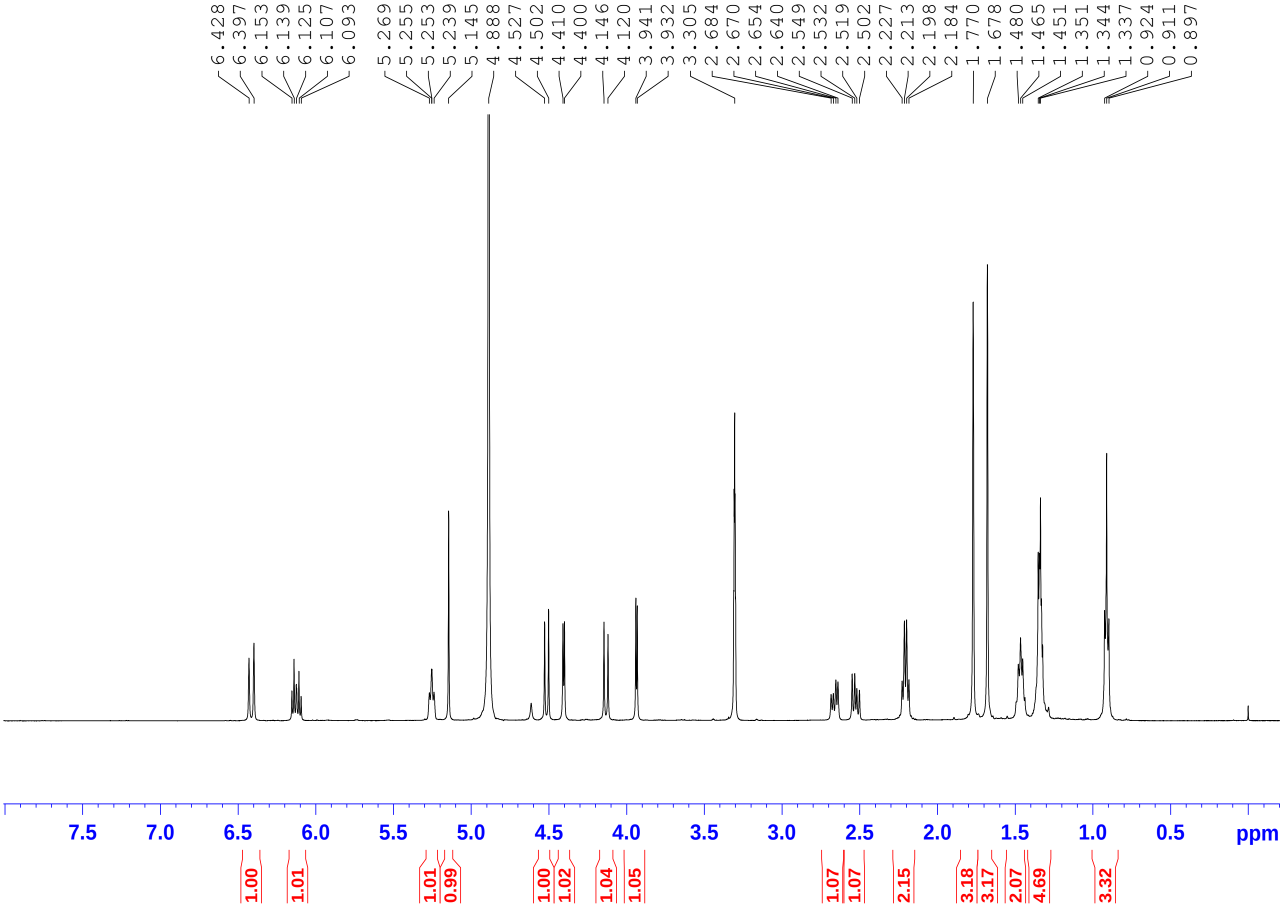

S48. ^{13}C NMR spectrum of cytosporin Y_2 (**5**) in MeOD-d_4.

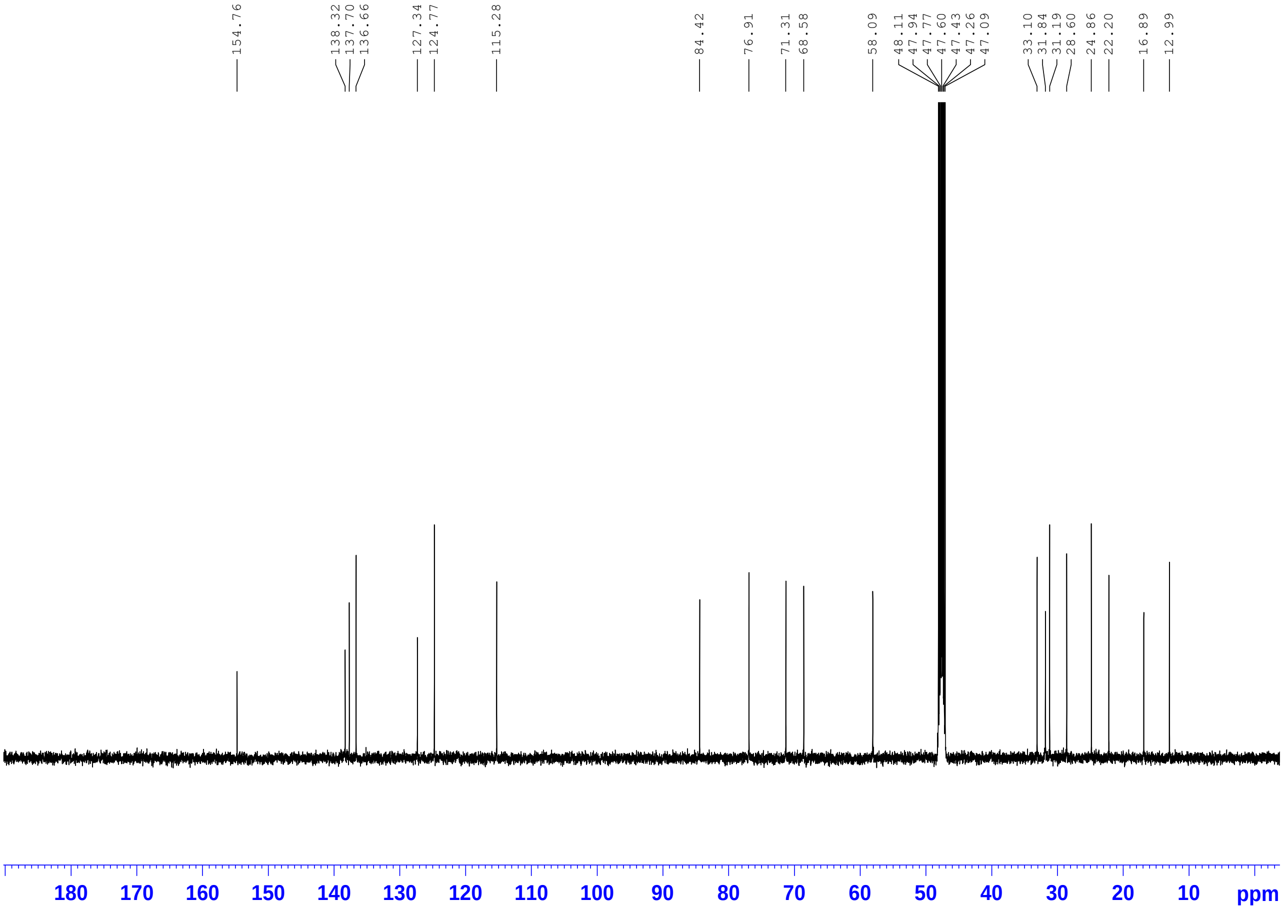

S49. DEPT135 spectrum of cytosporin Y_2 (**5**) in MeOD-d_4.

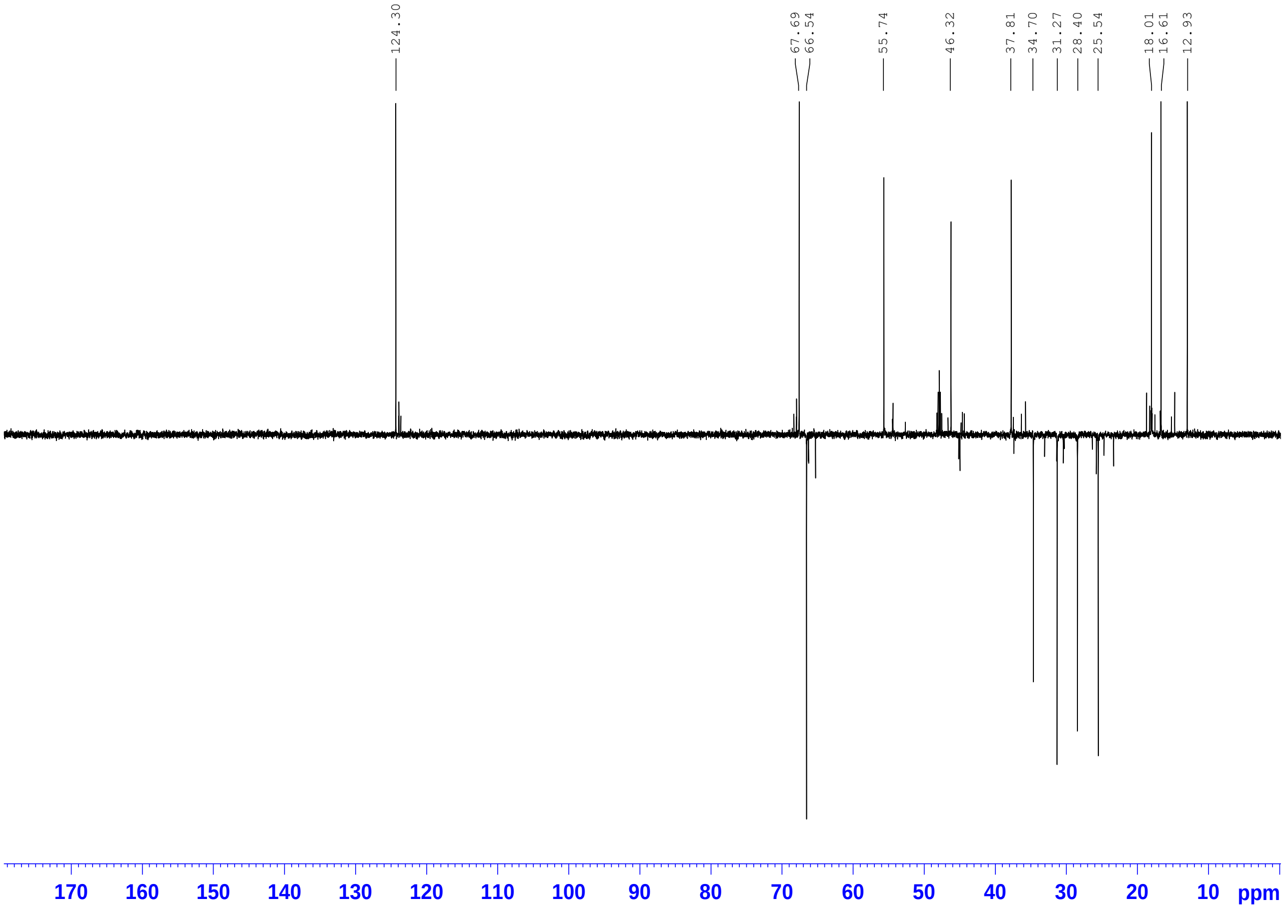

S50. HSQC spectrum of cytosporin Y_2 (**5**) in MeOD-d_4.

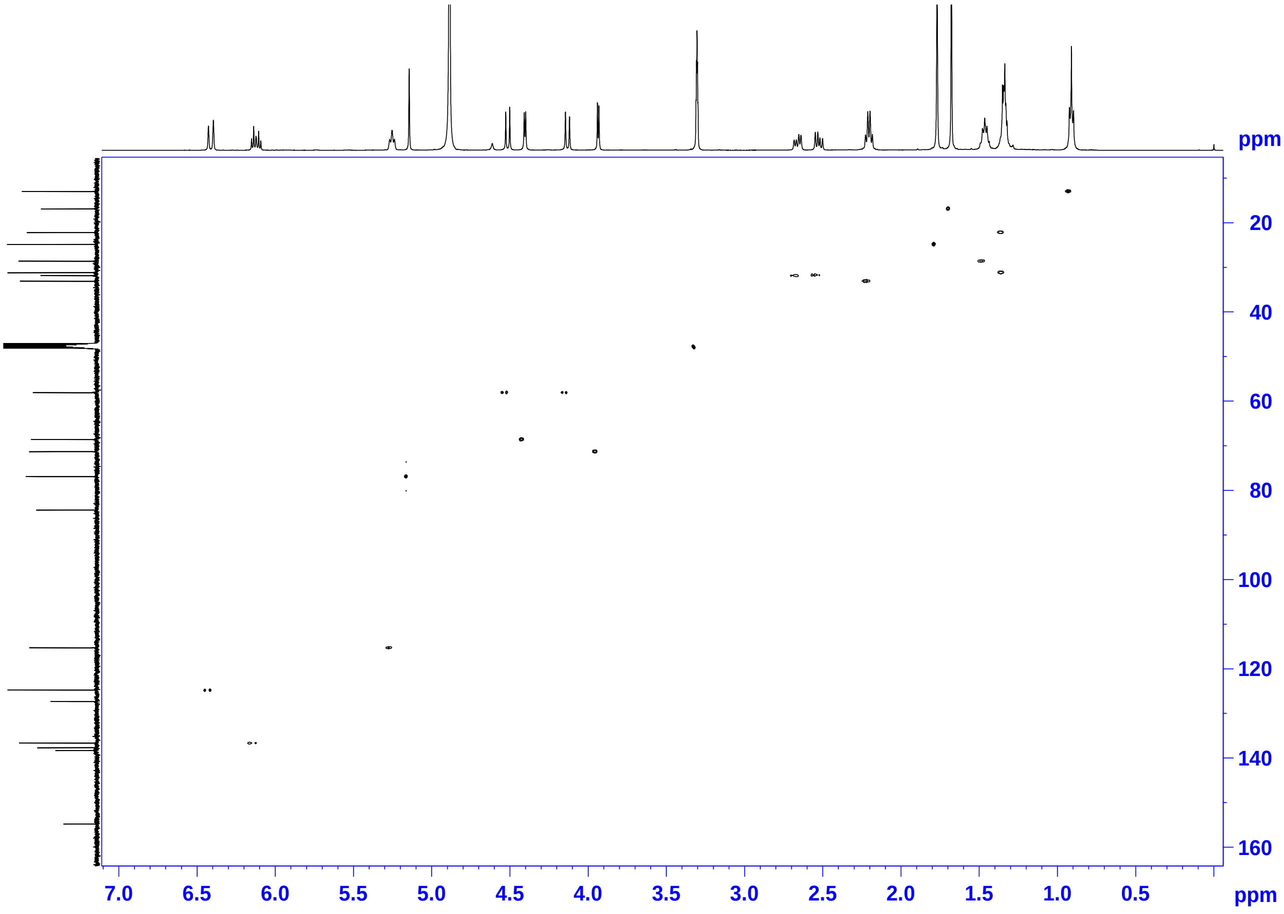

S51. COSY spectrum of cytosporin Y_2 (**5**) in MeOD-d_4.

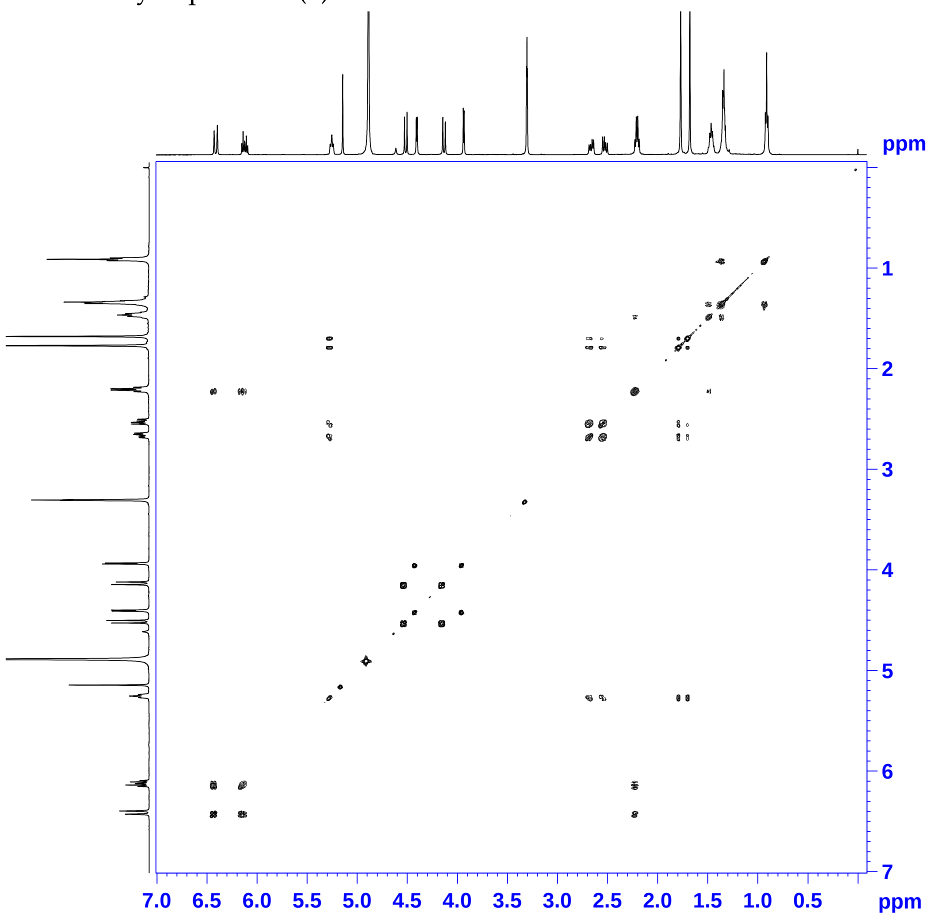

S52. HMBC spectrum of cytosporin Y_2 (**5**) in MeOD-d_4.

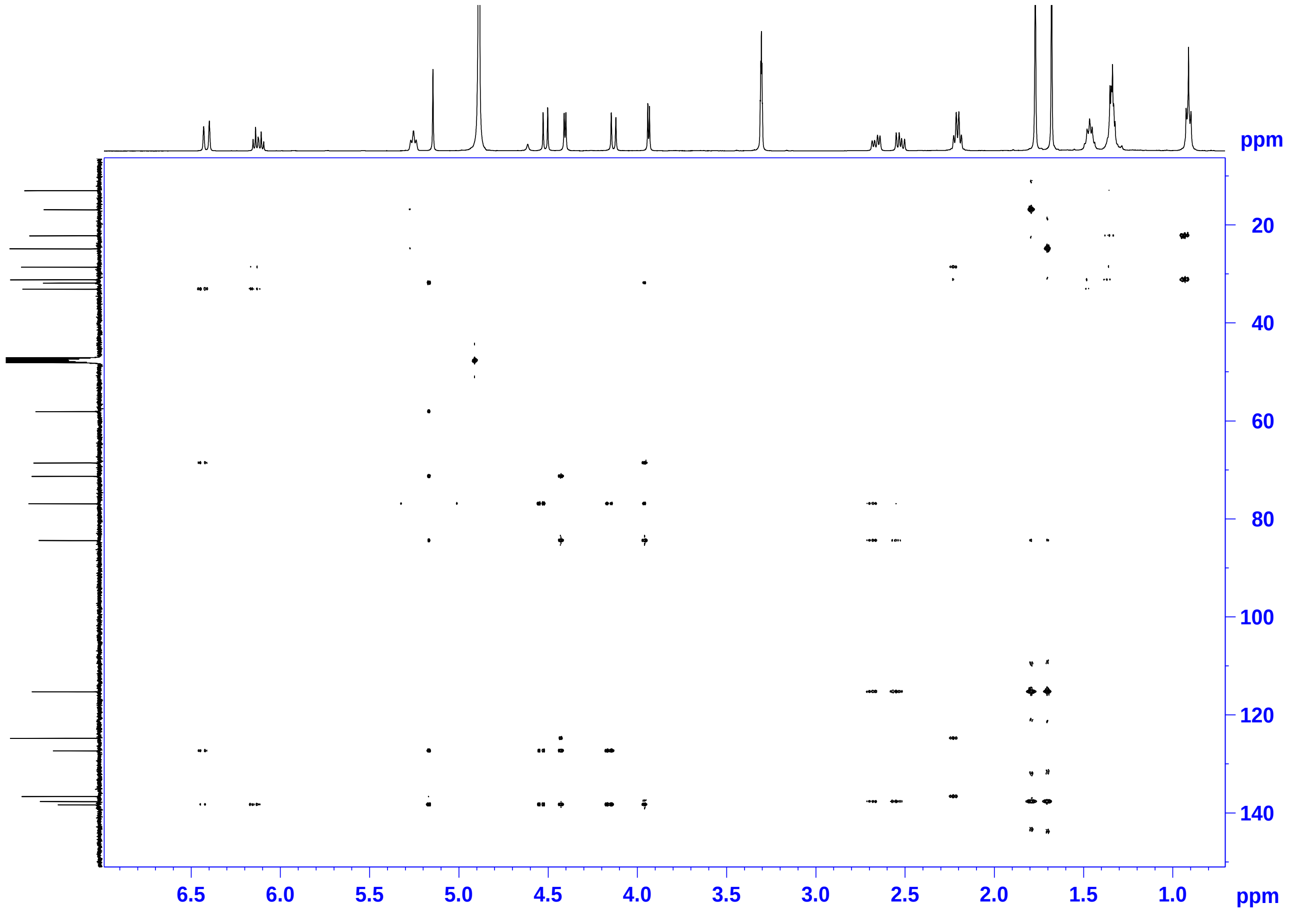

S53. NOESY spectrum of cytosporin Y_2 (**5**) in MeOD-d_4.

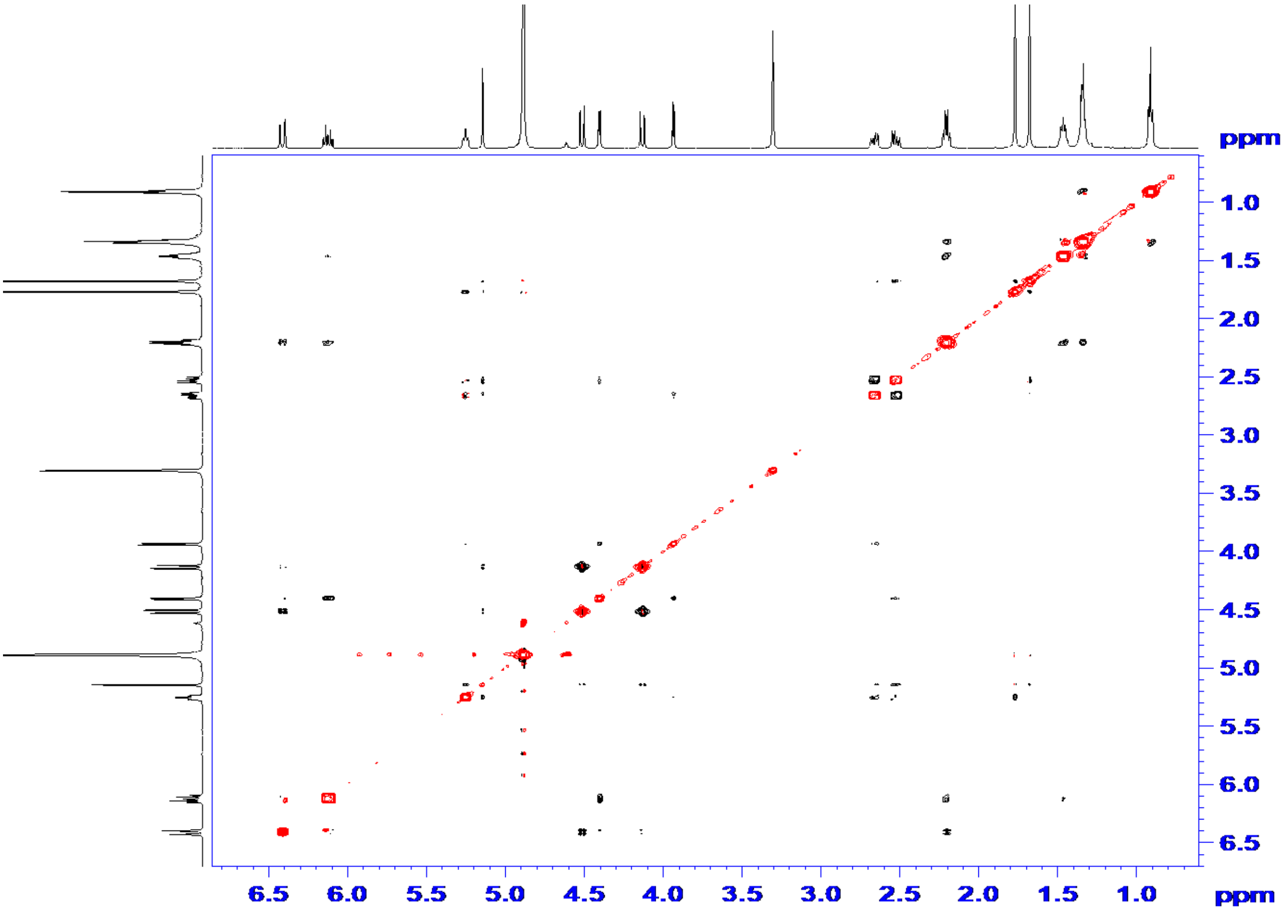

S54. HRESIMS of cytosporin Y_2 (**5**).

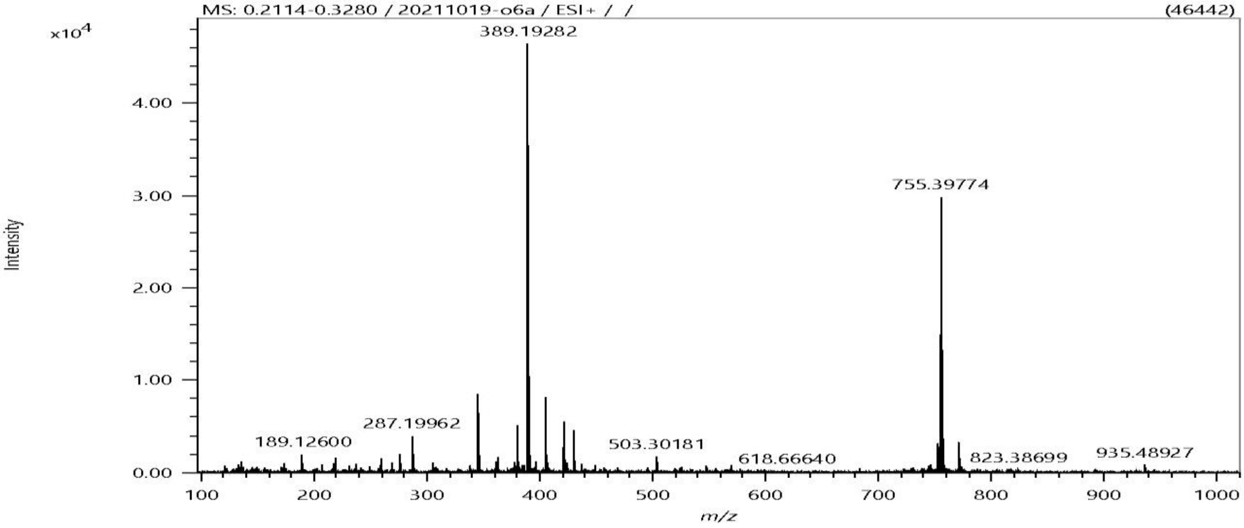

Elemental Composition

Parameters	
Tolerance:	±5.00 ppm
Electron:	Odd/Even
Charge:	+1
DBE:	-1.5 - 200.0

Elements Set 1:

Symbol	C	H	N	O	Na	S	Cl	Br
Min	0	0	0	0	1	0	0	0
Max	200	200	4	8	1	0	0	0

Symbol	P	Si	F
Min	0	0	0
Max	0	0	0

Results

Mass	Intensity	Intensity [%]	Formula	Calculated Mass	Mass Difference [mDa]	Mass Difference [ppm]	DBE
389.19282	46442.49	100.00	C20 H30 O6 Na	389.19346	-0.64	-1.64	5.5
			C18 H28 N3 O5 Na	389.19212	0.71	1.81	6.0

S55. UV spectrum of cytosporin Y2 (**5**) in in MeOH.

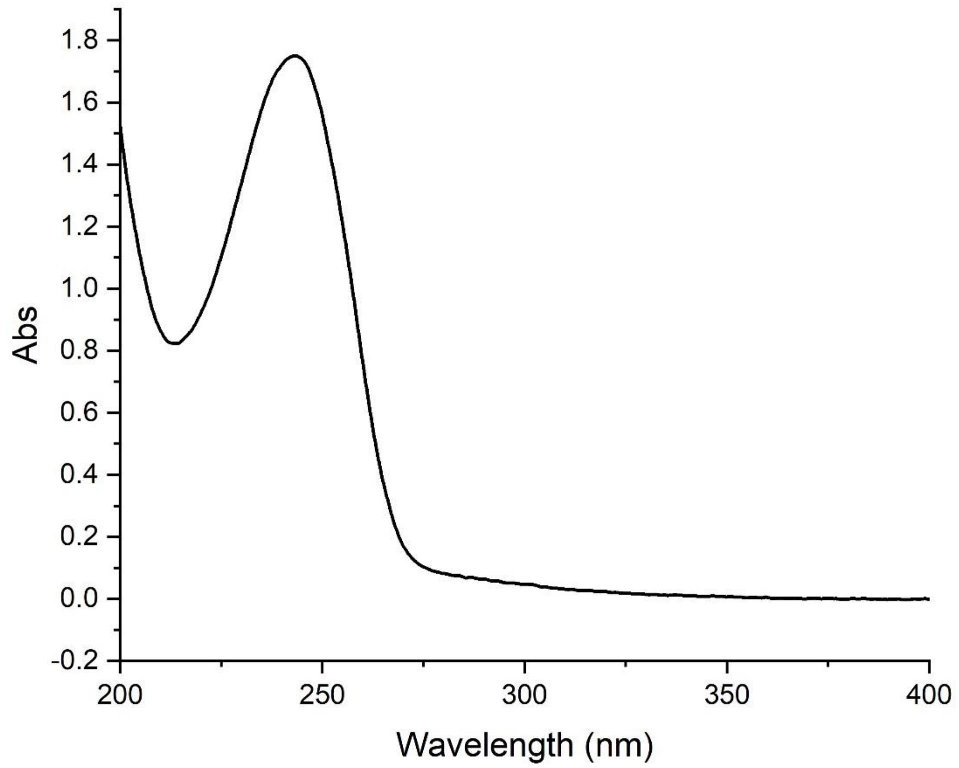

S56. IR spectrum of cytosporin Y2 (**5**) (KBr).

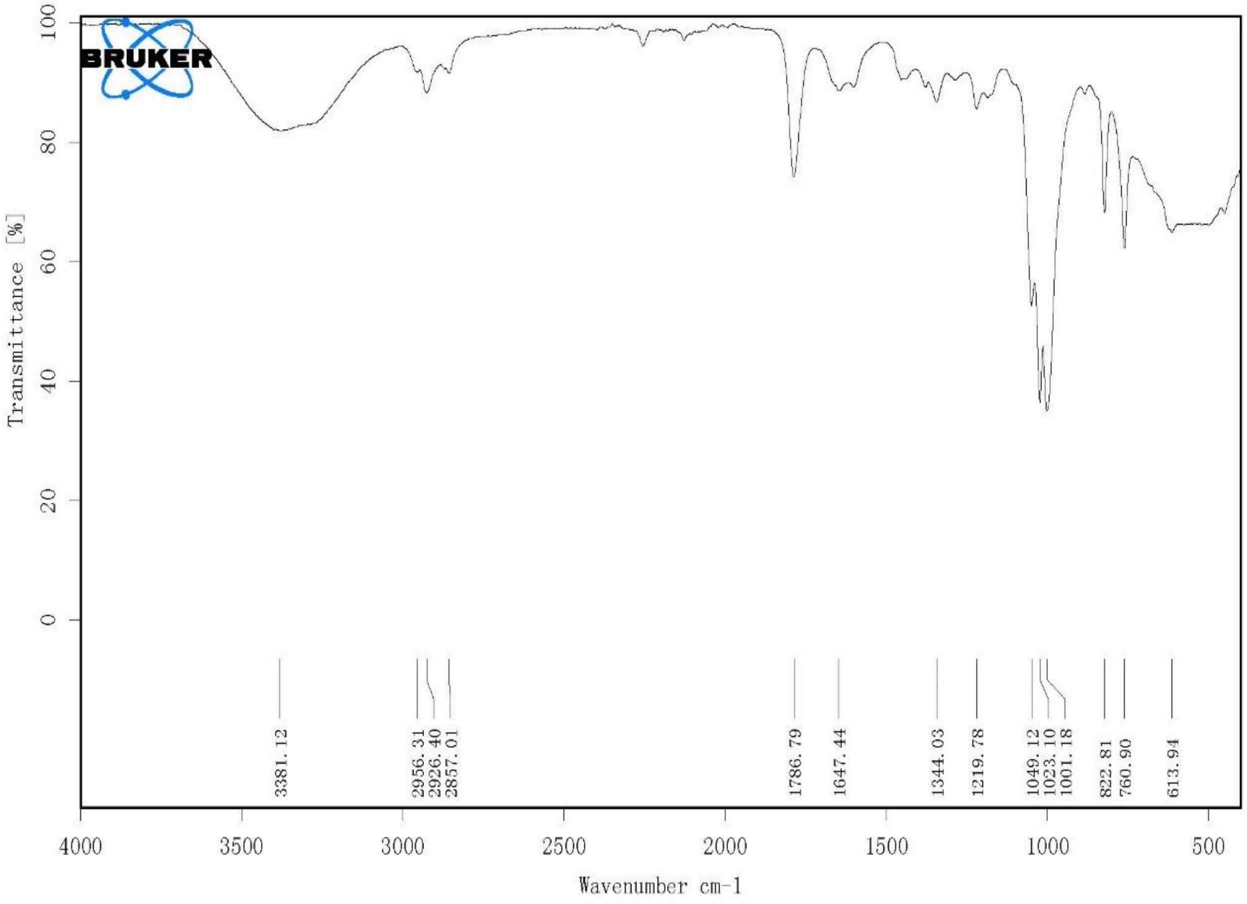

S57. ^{1}H NMR spectrum of cytosporin Y3 (**6**) in $CDCl_3$.

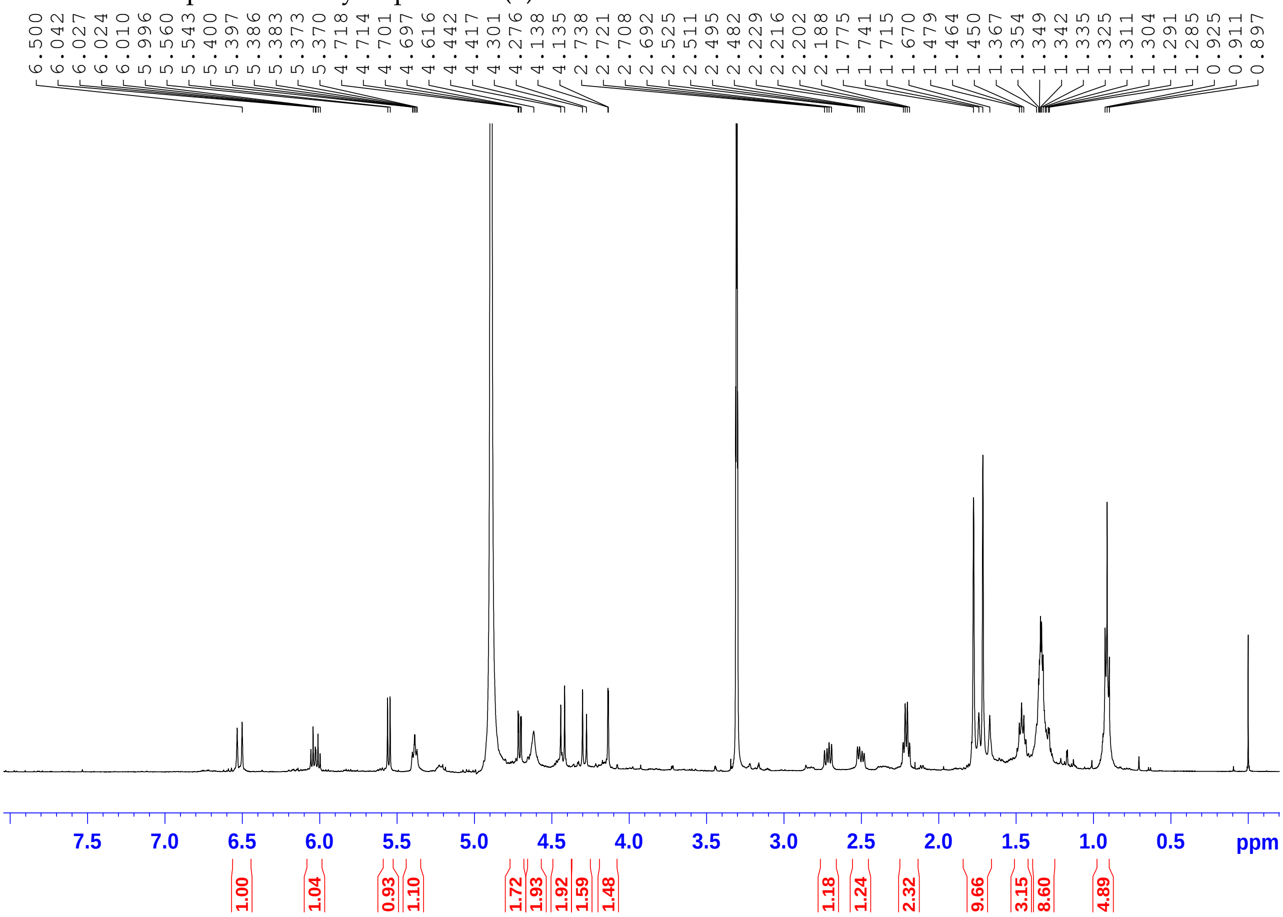

S58. ^{13}C NMR spectrum of cytosporin Y3 (**6**) in $CDCl_3$.

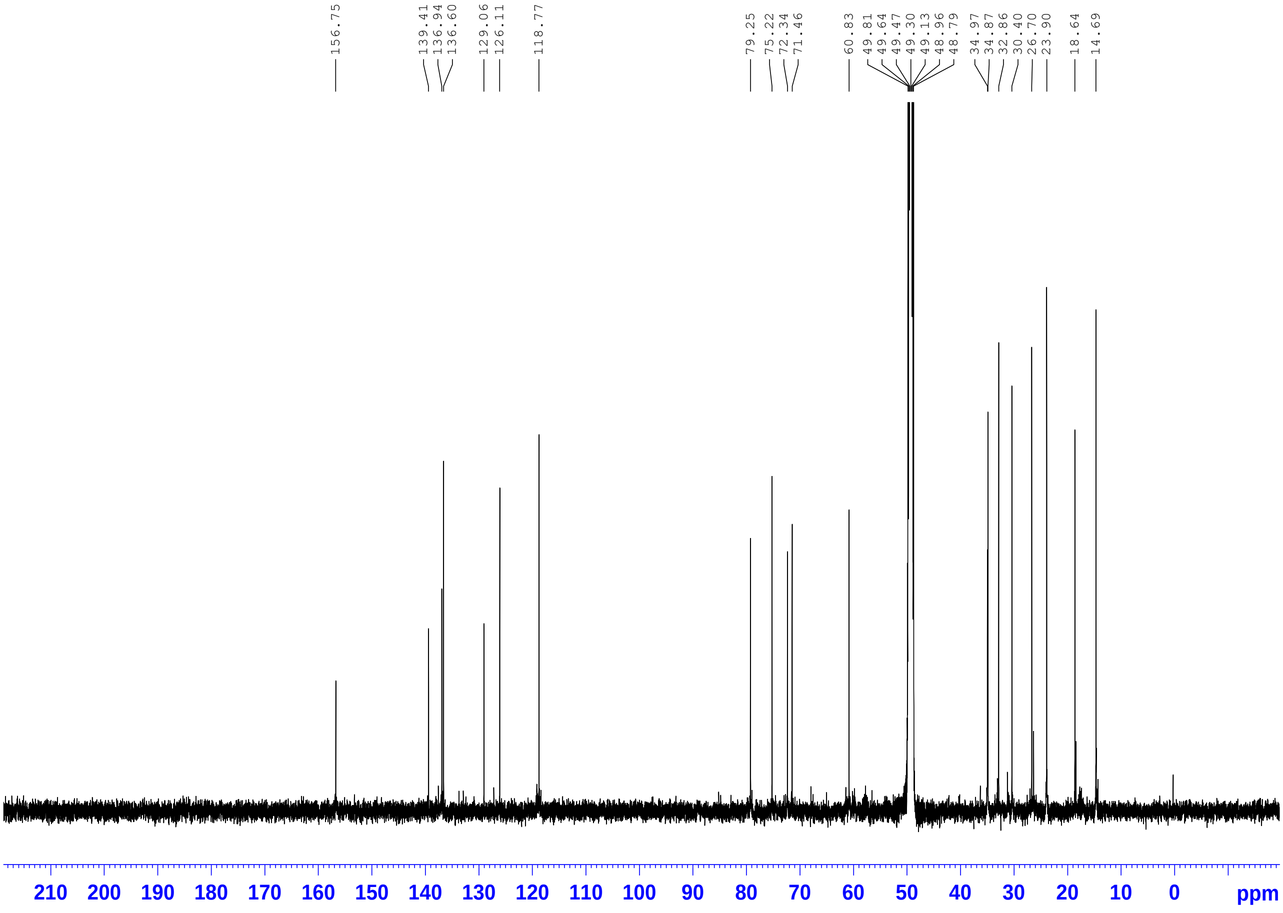

S59. DEPT135 spectrum of cytosporin Y3 (**6**) in CDCl3.

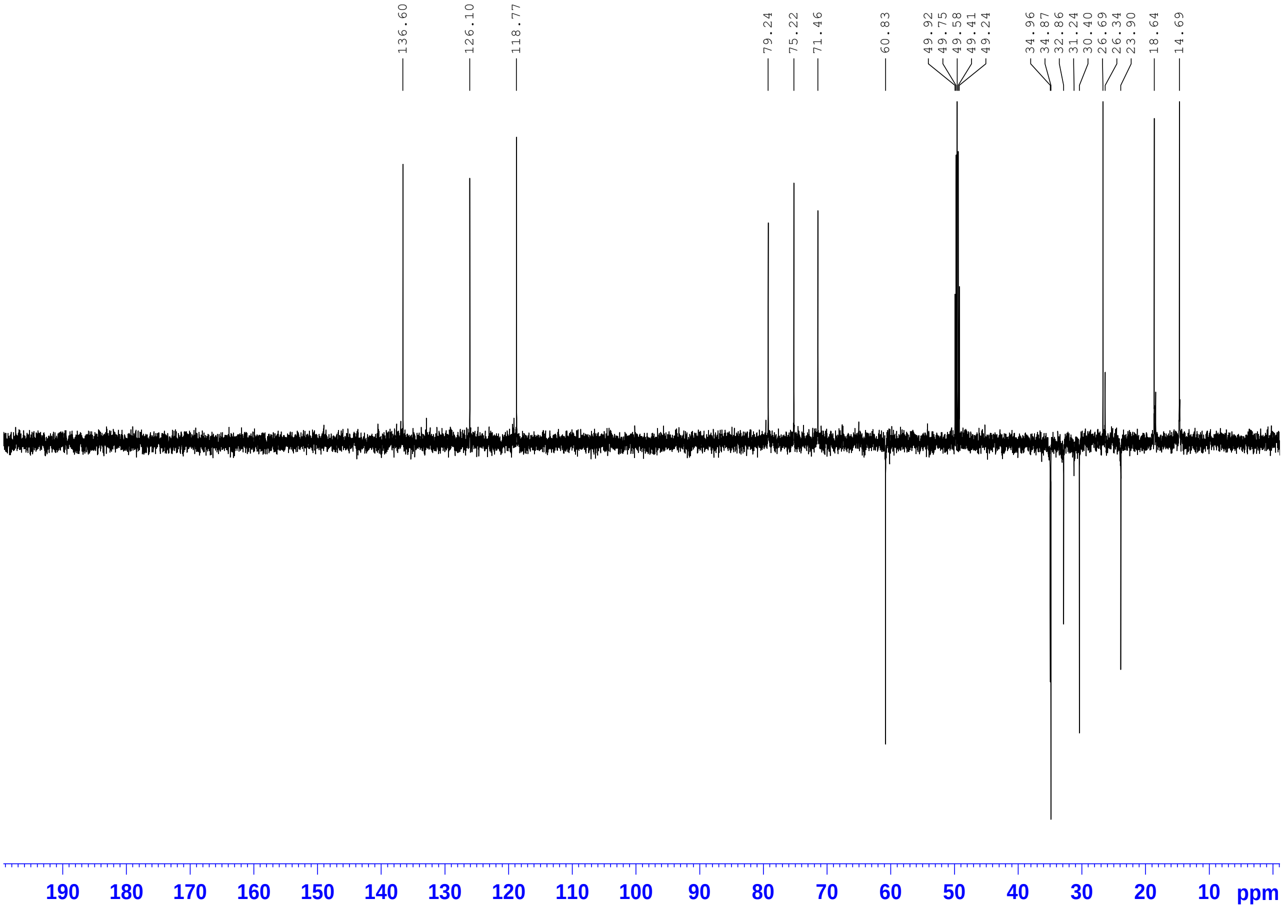

S60. HSQC spectrum of cytosporin Y3 (**6**) in CDCl3.

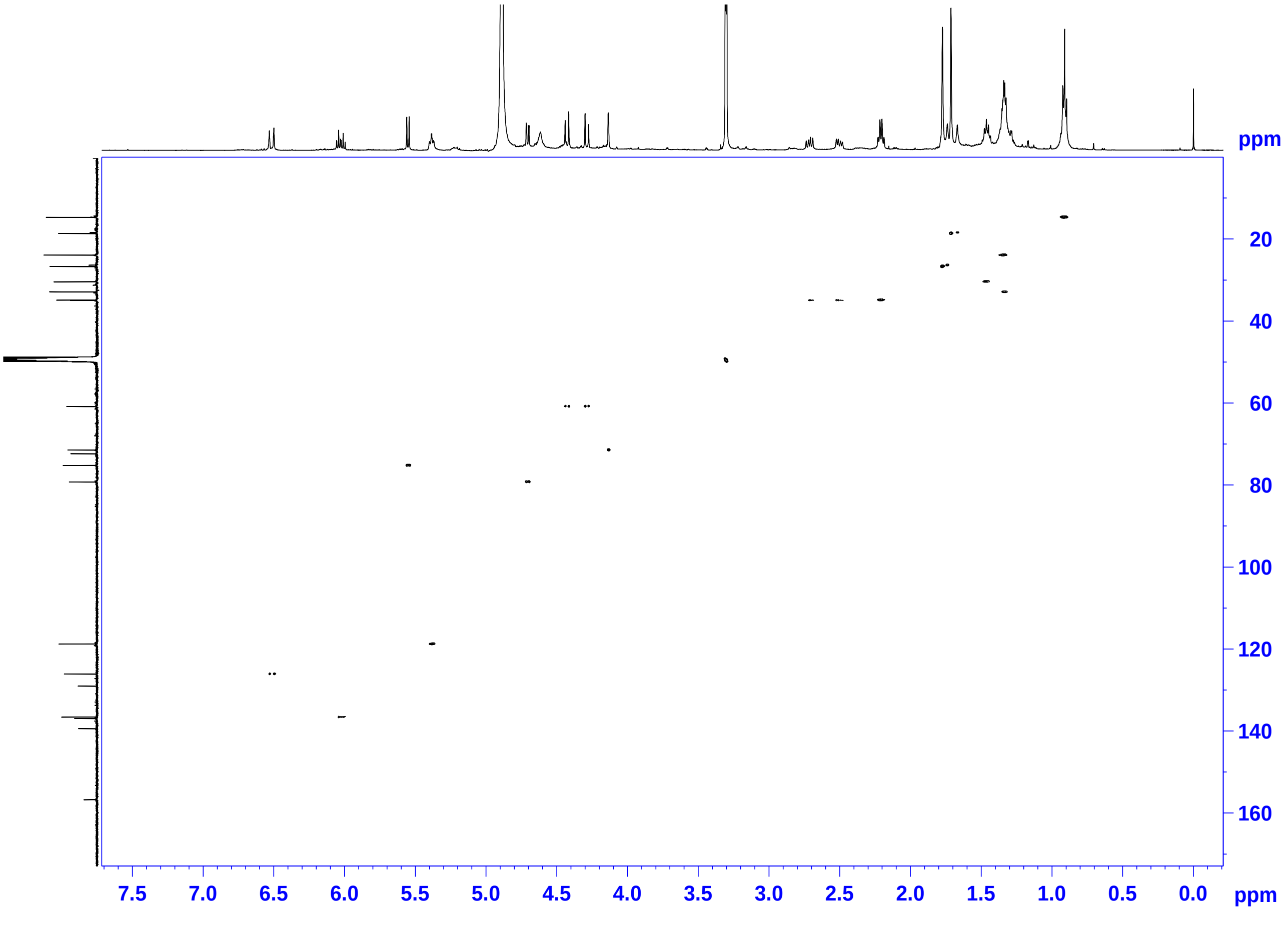

S61. COSY spectrum of cytosporin Y3 (**6**) in $CDCl_3$.

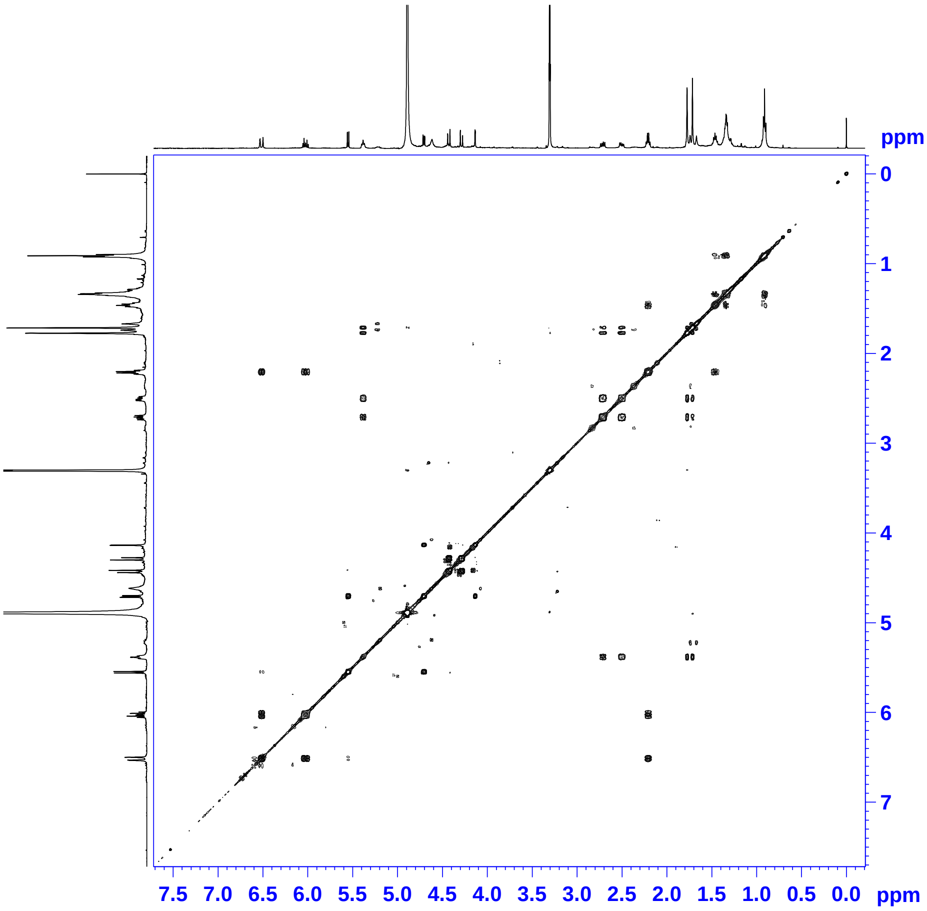

S62. HMBC spectrum of cytosporin Y3 (**6**) in $CDCl_3$.

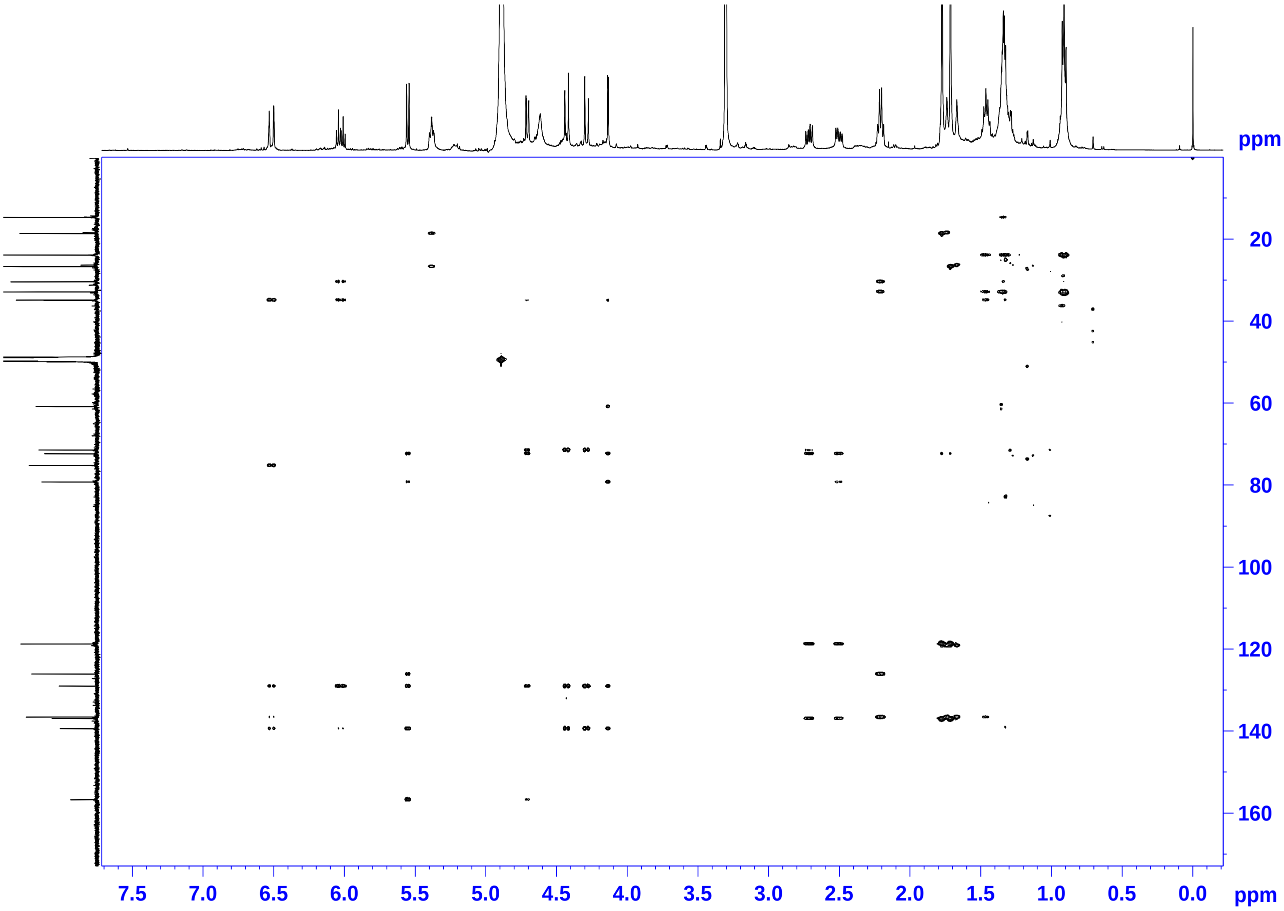

S63. NOESY spectrum of cytosporin Y_3 (**6**) in $CDCl_3$.

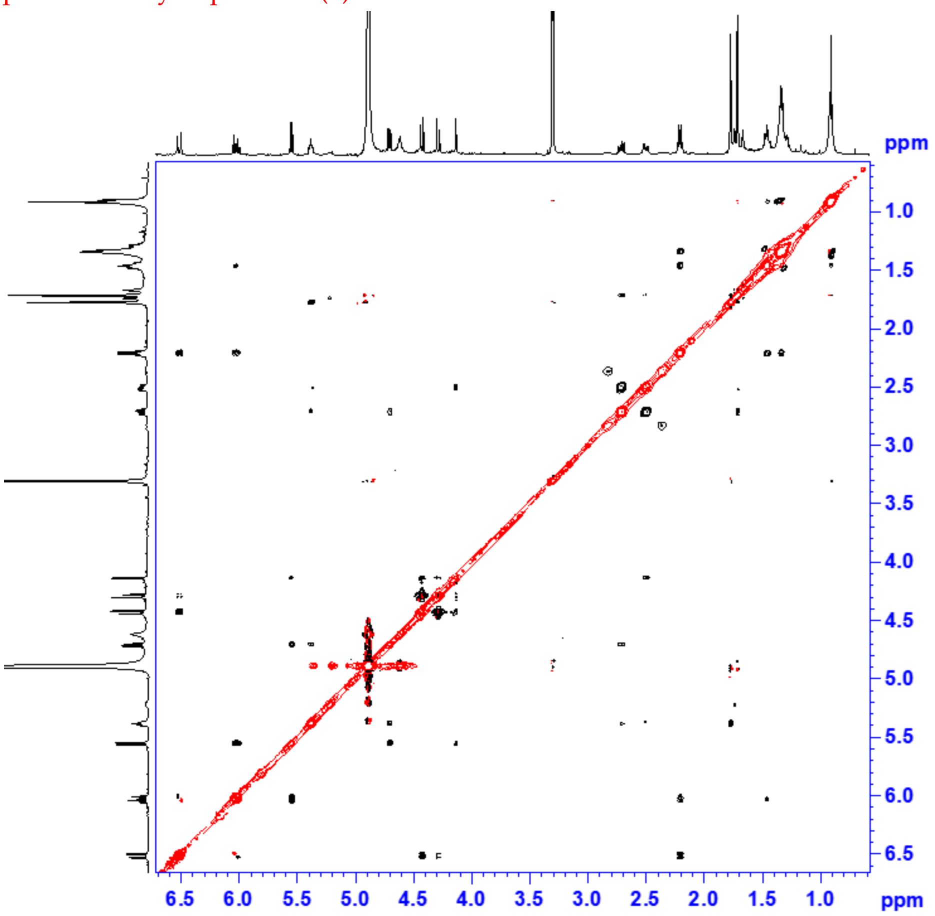

S64. HRESIMS of cytosporin Y_3 (**6**).

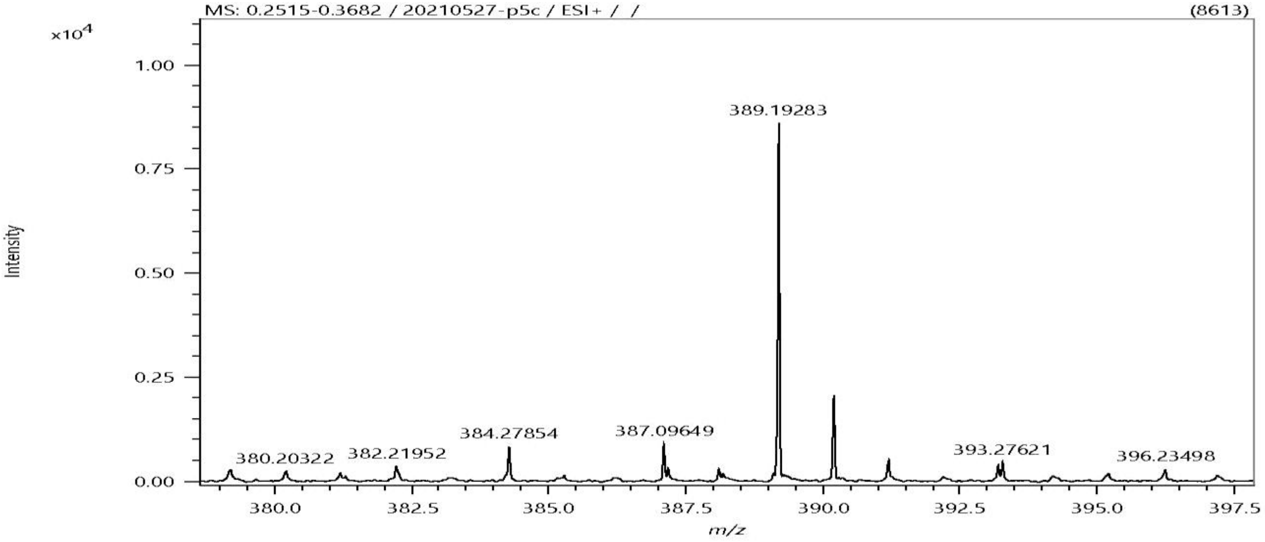

Elemental Composition

Parameters

Tolerance:	±5.00 ppm
Electron:	Odd/Even
Charge:	+1
DBE:	-1.5 - 200.0

Elements Set 1:

Symbol	C	H	N	O	Na	S	Cl	Br
Min	0	0	0	0	1	0	0	0
Max	200	200	0	8	1	0	0	0

Symbol	F	Si
Min	0	0
Max	0	0

Results

Mass	Intensity	Intensity [%]	Formula	Calculated Mass	Mass Difference [mDa]	Mass Difference [ppm]	DBE
389.19283	8613.29	11.79	C20 H30 O6 Na	389.19346	-0.63	-1.62	5.5

S65. UV spectrum of cytosporin Y3 (**6**) in MeOH.

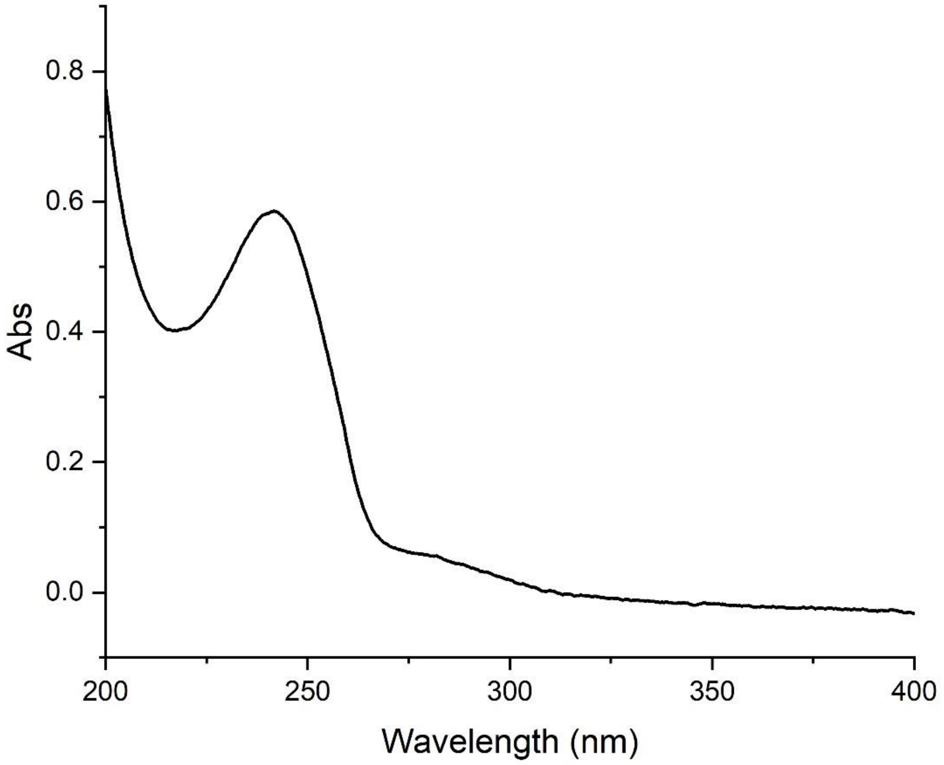

S66. IR spectrum of cytosporin Y3 (**6**) (KBr).

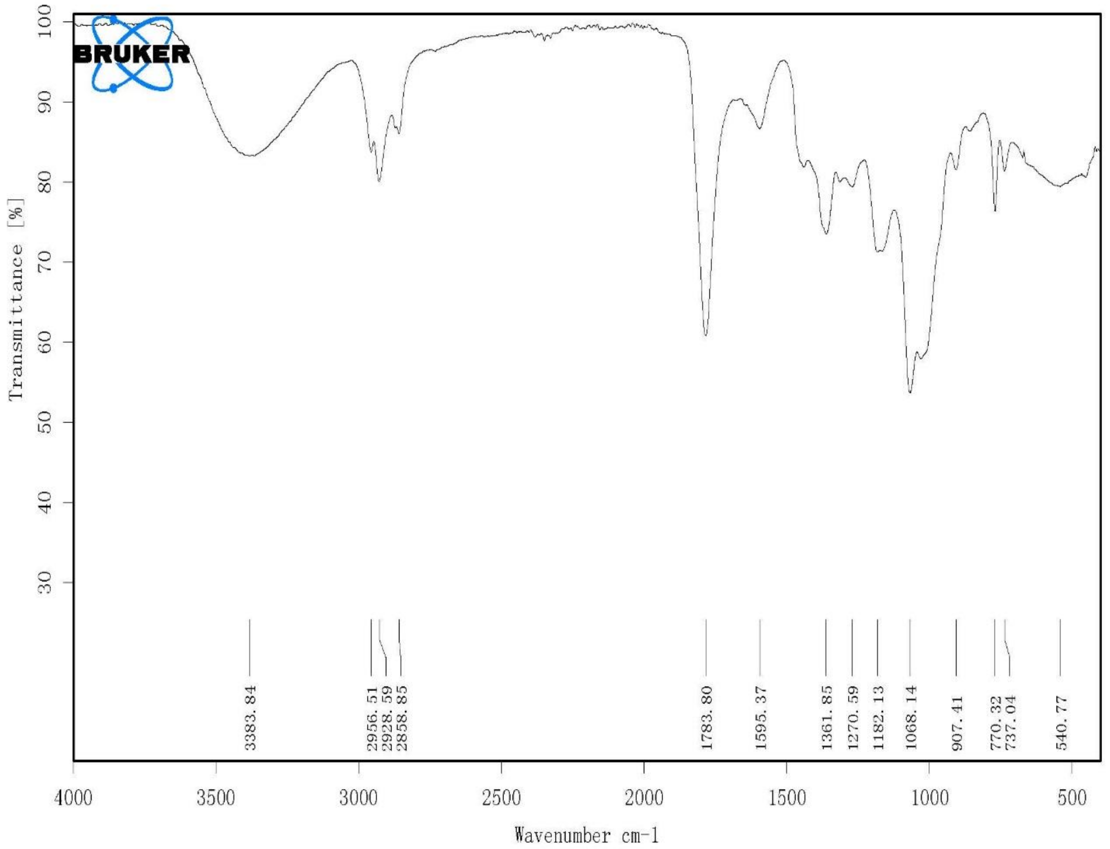

S67. ¹H NMR spectrum of cytosporin E₁ (**7**) in $CDCl_3$..

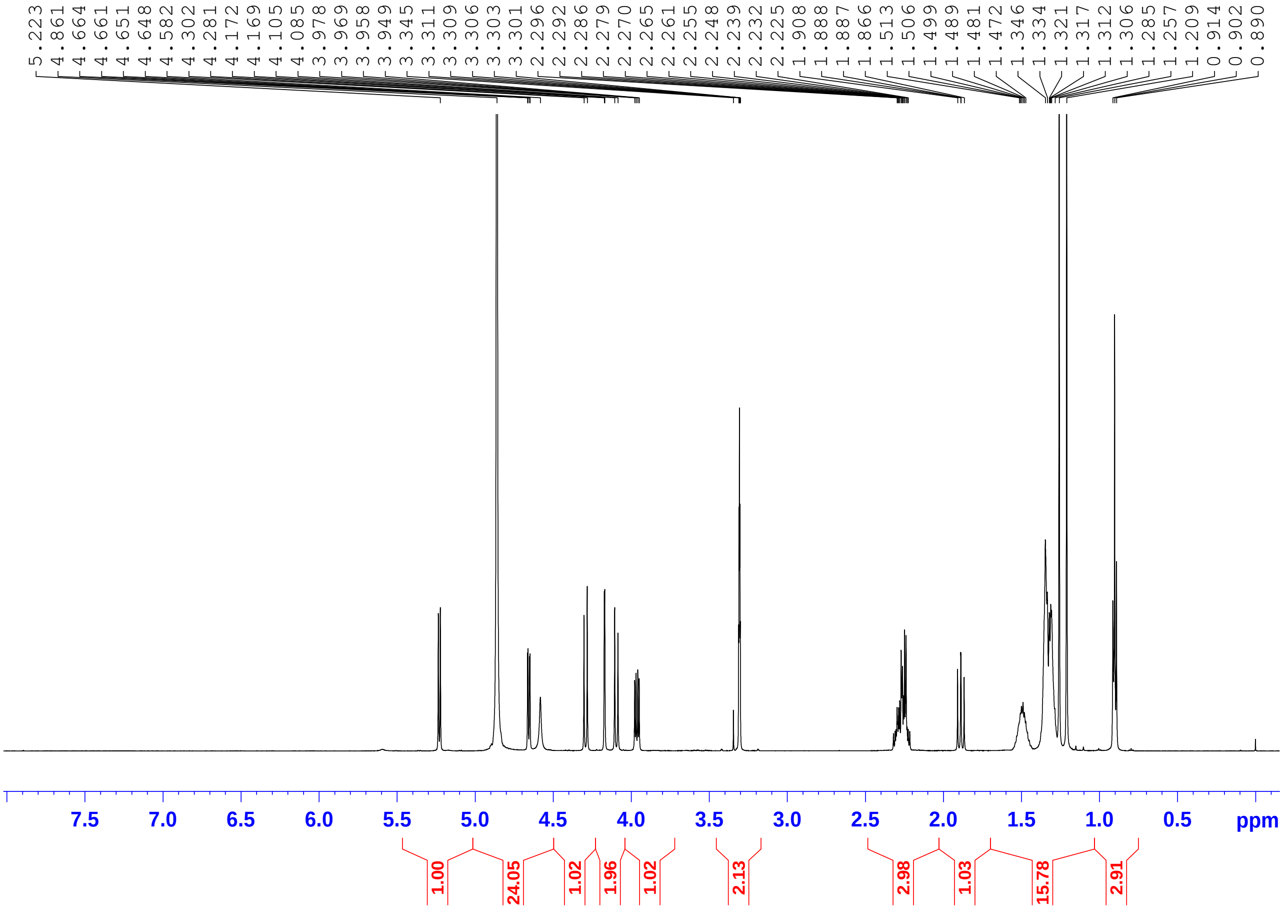

S68. ¹H NMR spectrum of cytosporin E₁ (**7**) in DMSO-*d*₆.

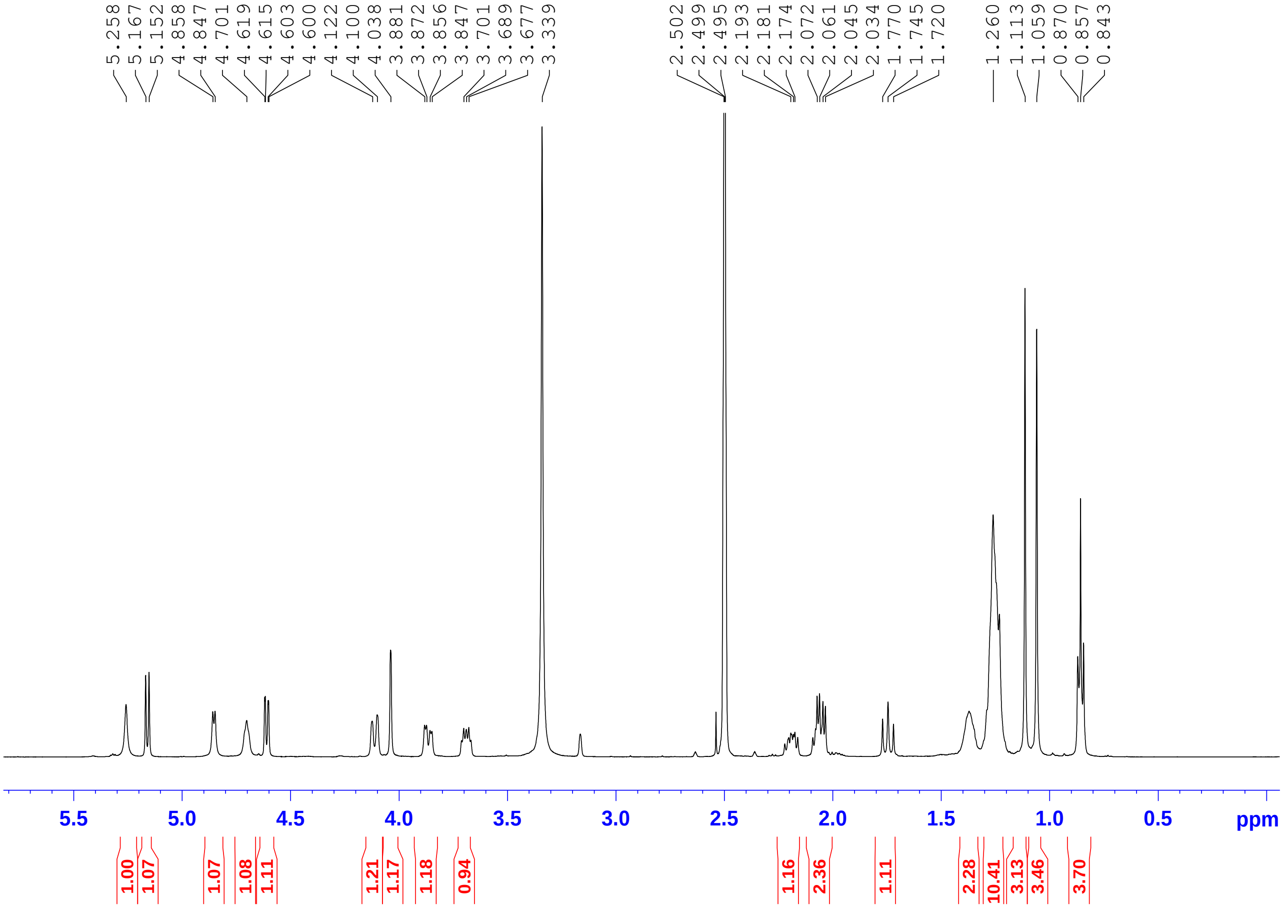

S69. ^{13}C NMR spectrum of cytosporin E_1 (**7**) in $CDCl_3$.

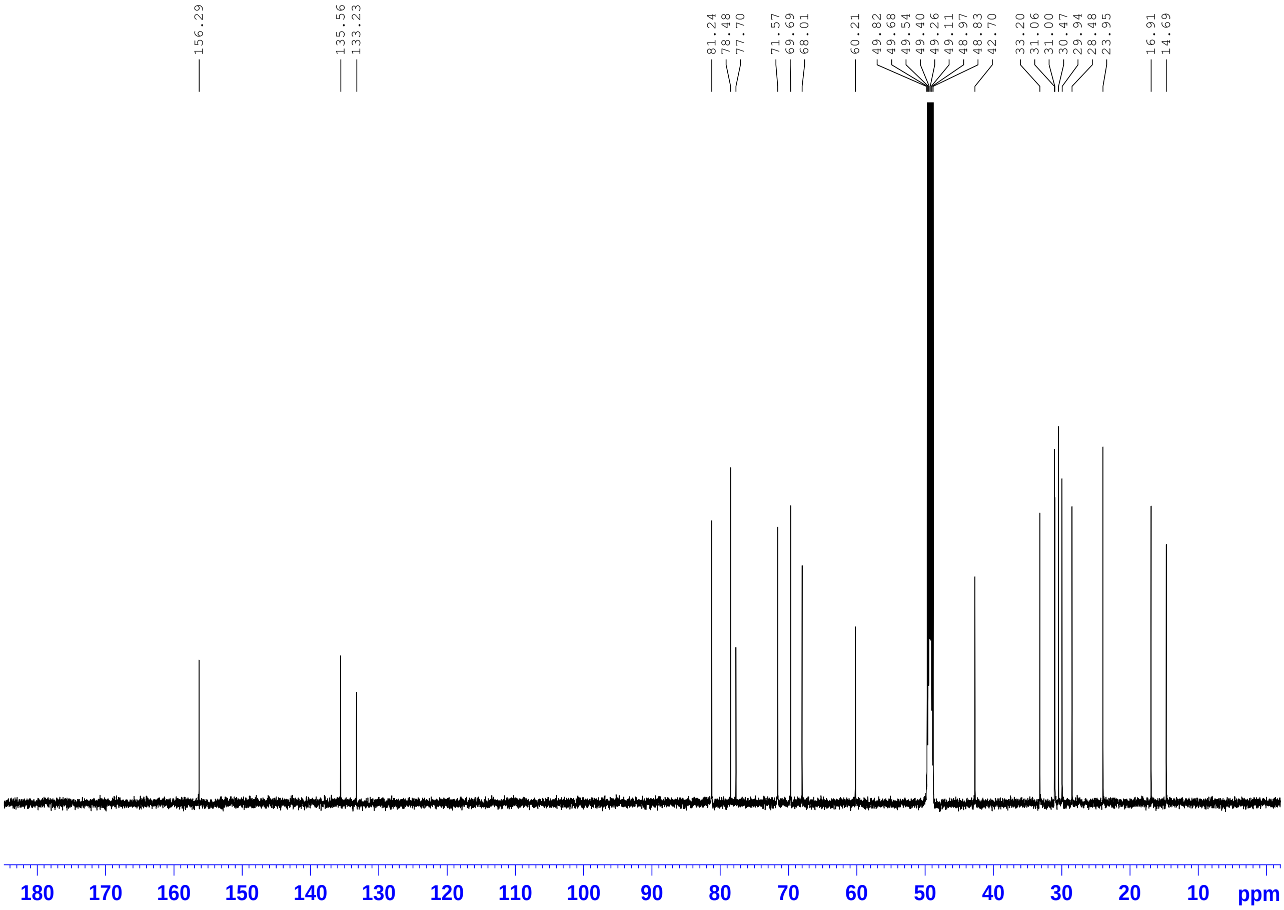

S70. DEPT135 spectrum of cytosporin E_1 (**7**) in $CDCl_3$.

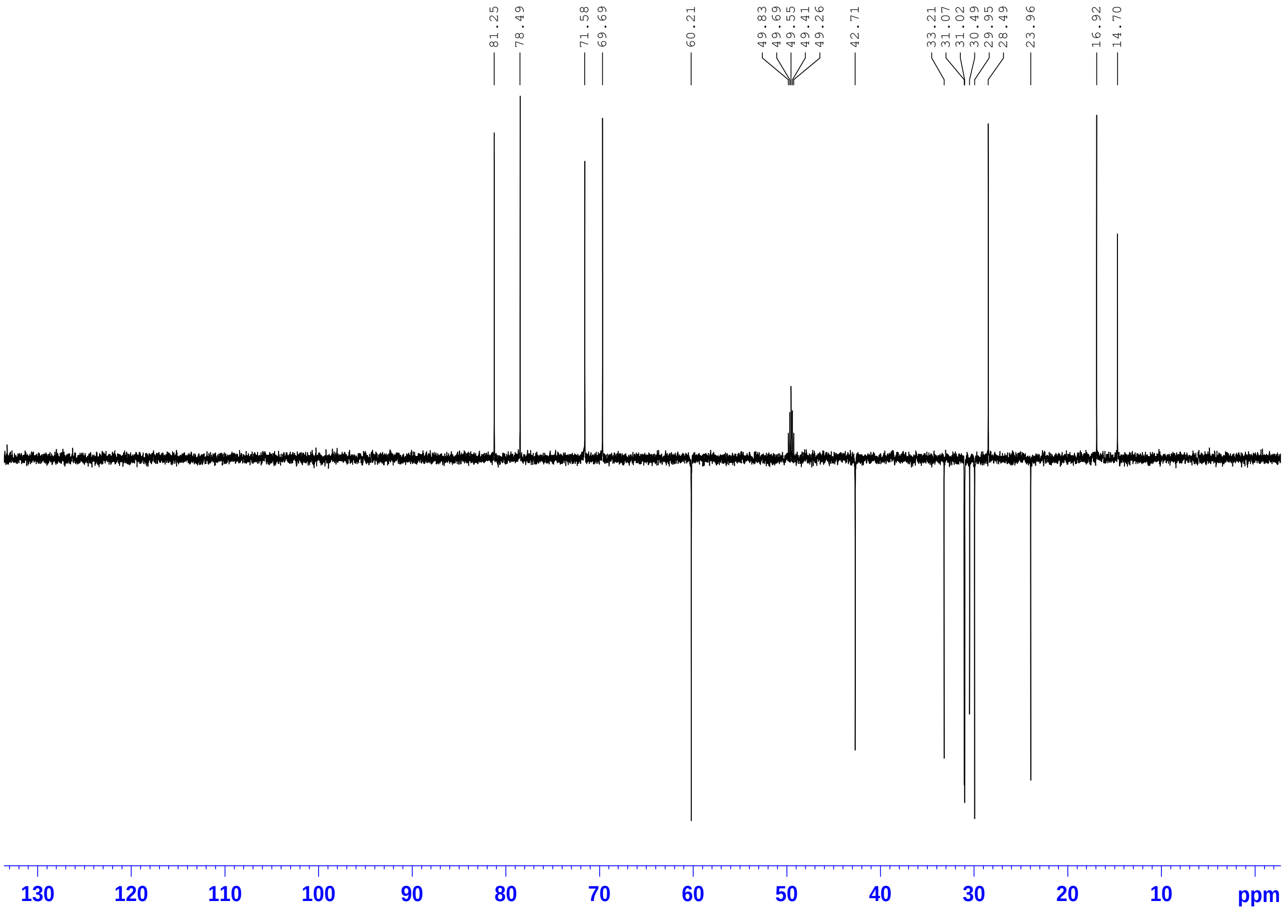

S71. HSQC spectrum of cytosporin E1 (**7**) in $CDCl_3$.

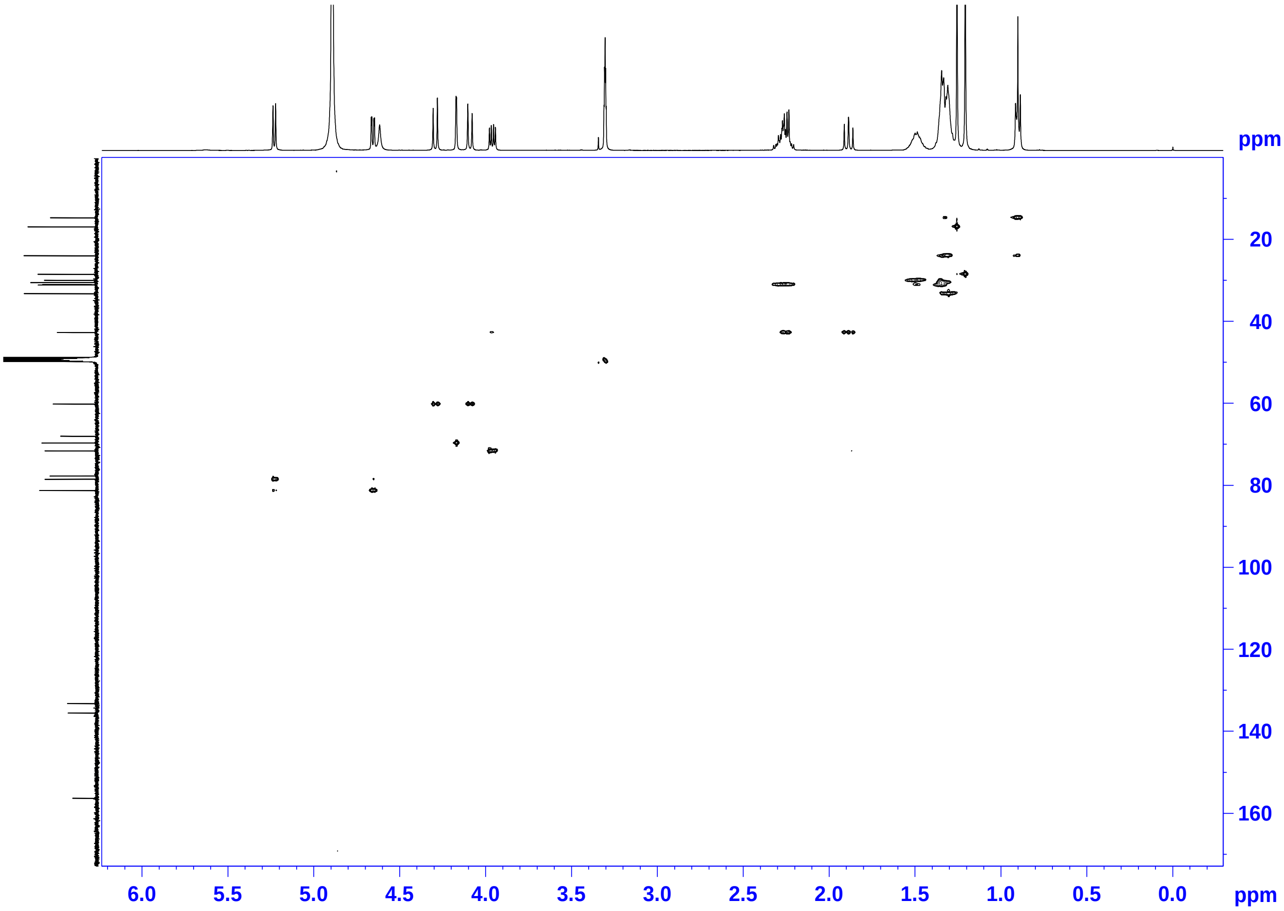

S72. COSY spectrum of cytosporin E1 (**7**) in $CDCl_3$.

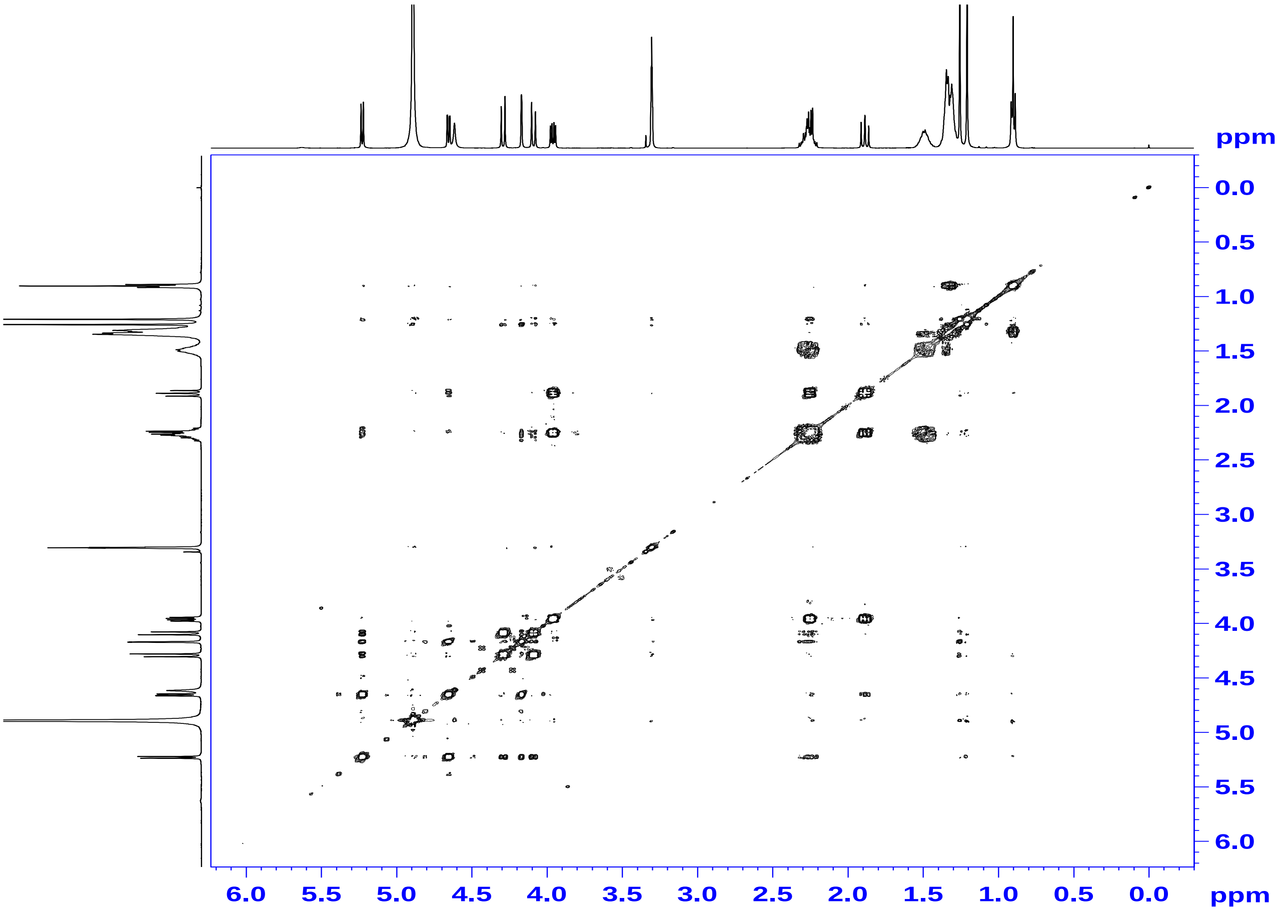

S73. HMBC spectrum of cytosporin E1 (**7**) in $CDCl_3$.

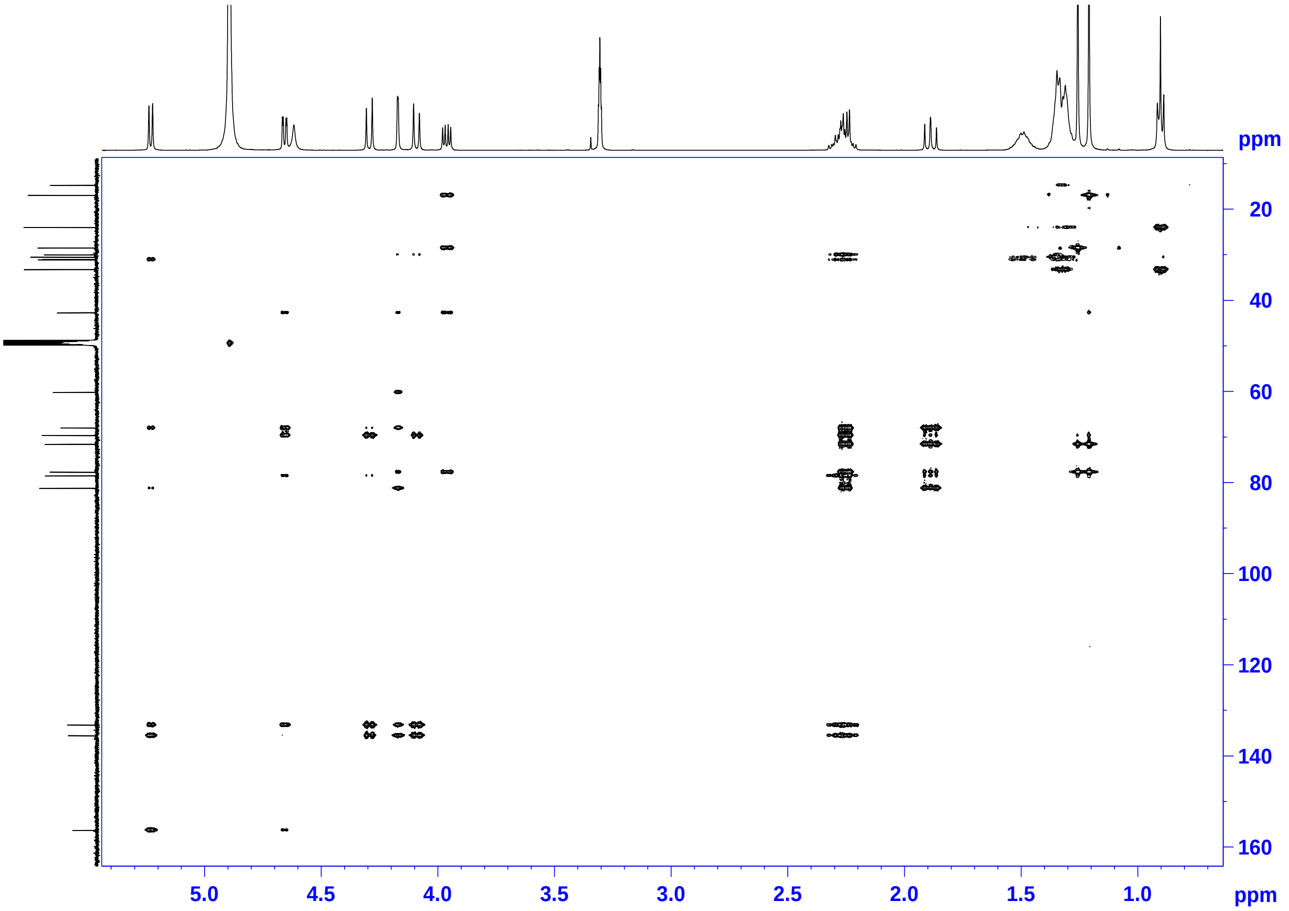

S74. NOESY spectrum of cytosporin E1 (**7**) in $CDCl_3$.

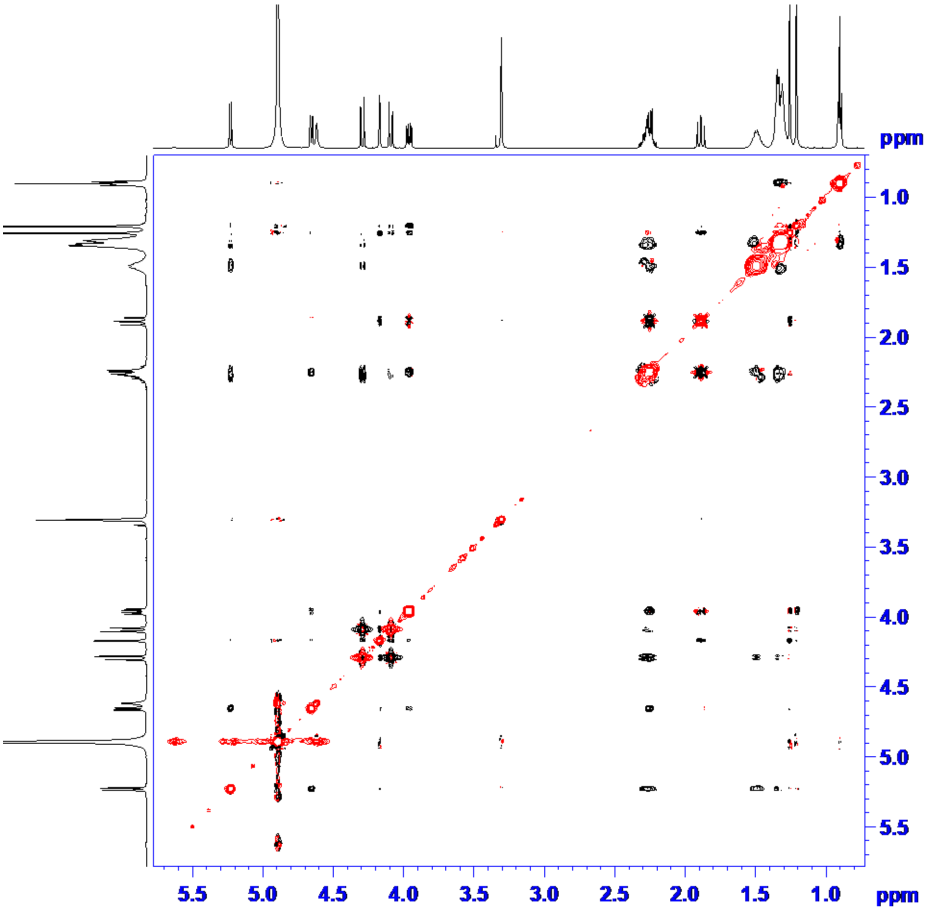

S75. NOESY spectrum of cytosporin E1 (7) in DMSO-d_6.

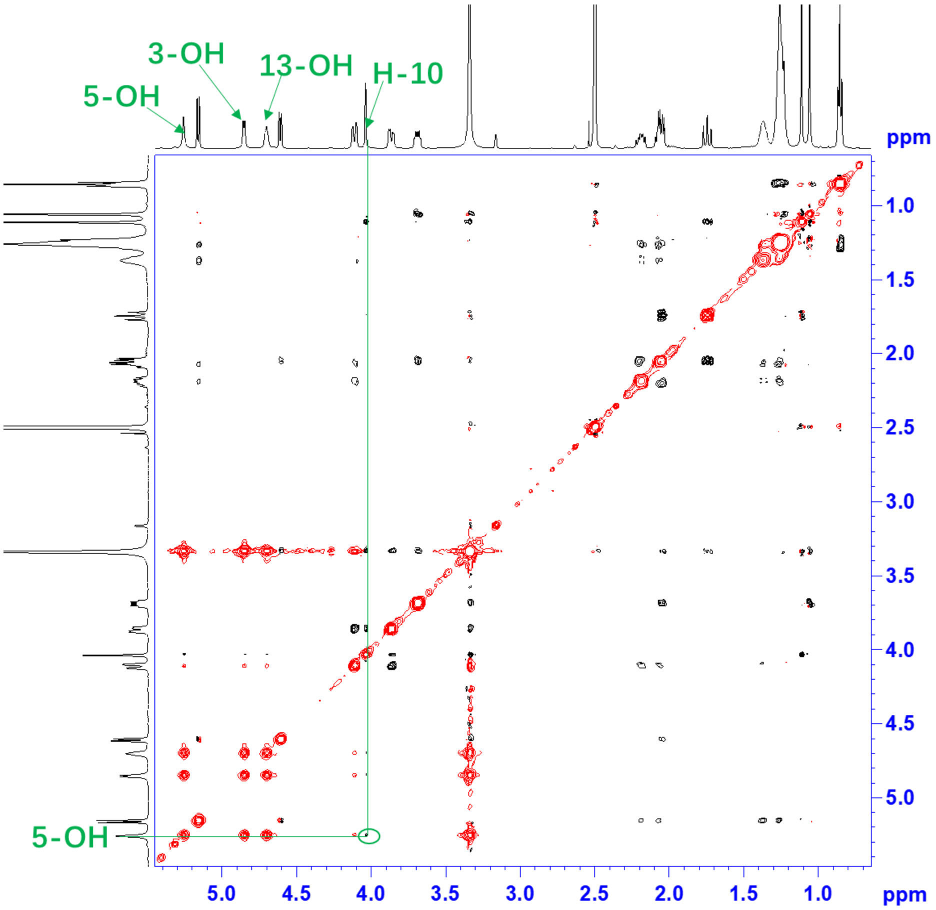

S76. HRESIMS of cytosporin E1 (7).

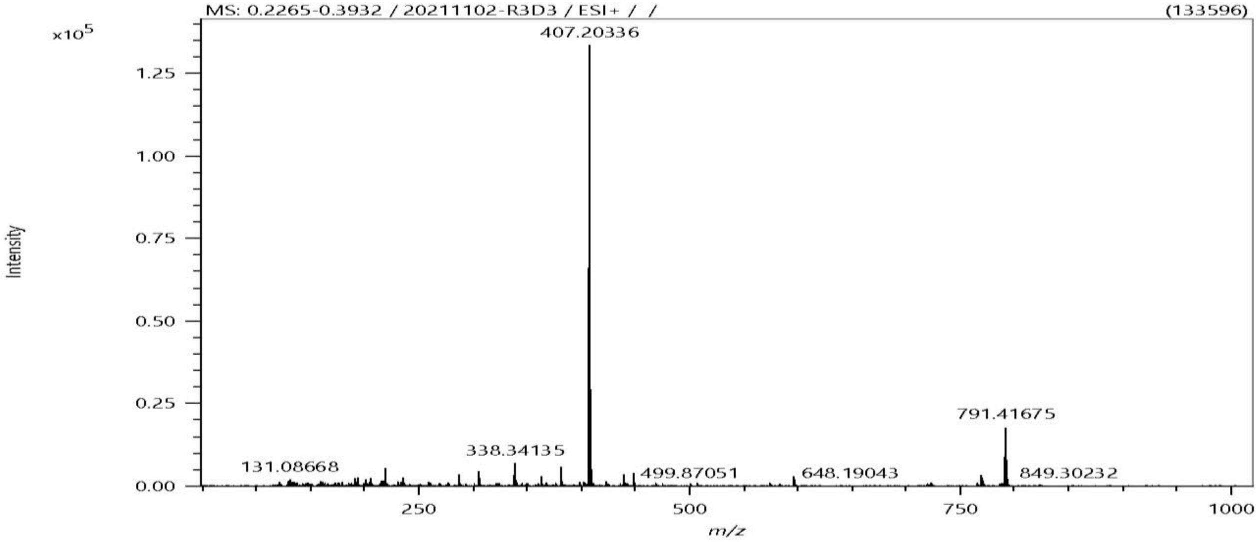

Elemental Composition

Parameters

Tolerance:	±5.00 ppm
Electron:	Odd/Even
Charge:	+1
DBE:	-1.5 - 200.0

Elements Set 1:

Symbol	C	H	N	O	Na	S	Cl	Br
Min	0	0	0	0	1	0	0	0
Max	200	200	0	10	1	0	0	0

Symbol	P	I	F	D
Min	0	0	0	0
Max	0	0	0	0

Results

Mass	Intensity	Intensity [%]	Formula	Calculated Mass	Mass Difference [mDa]	Mass Difference [ppm]	DBE
407.20336	133596.09	100.00	C20 H32 O7 Na	407.20402	-0.66	-1.63	4.5

S77. UV spectrum of cytosporin E1 (**7**) in MeOH.

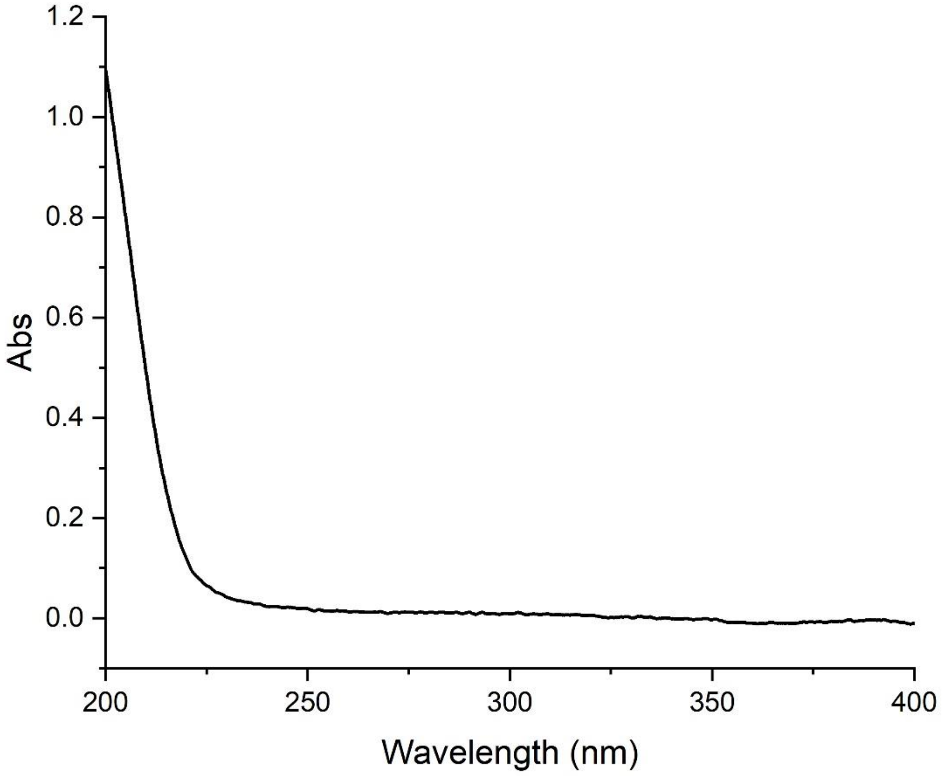

S78. IR spectrum of cytosporin E1 (**7**) (KBr).

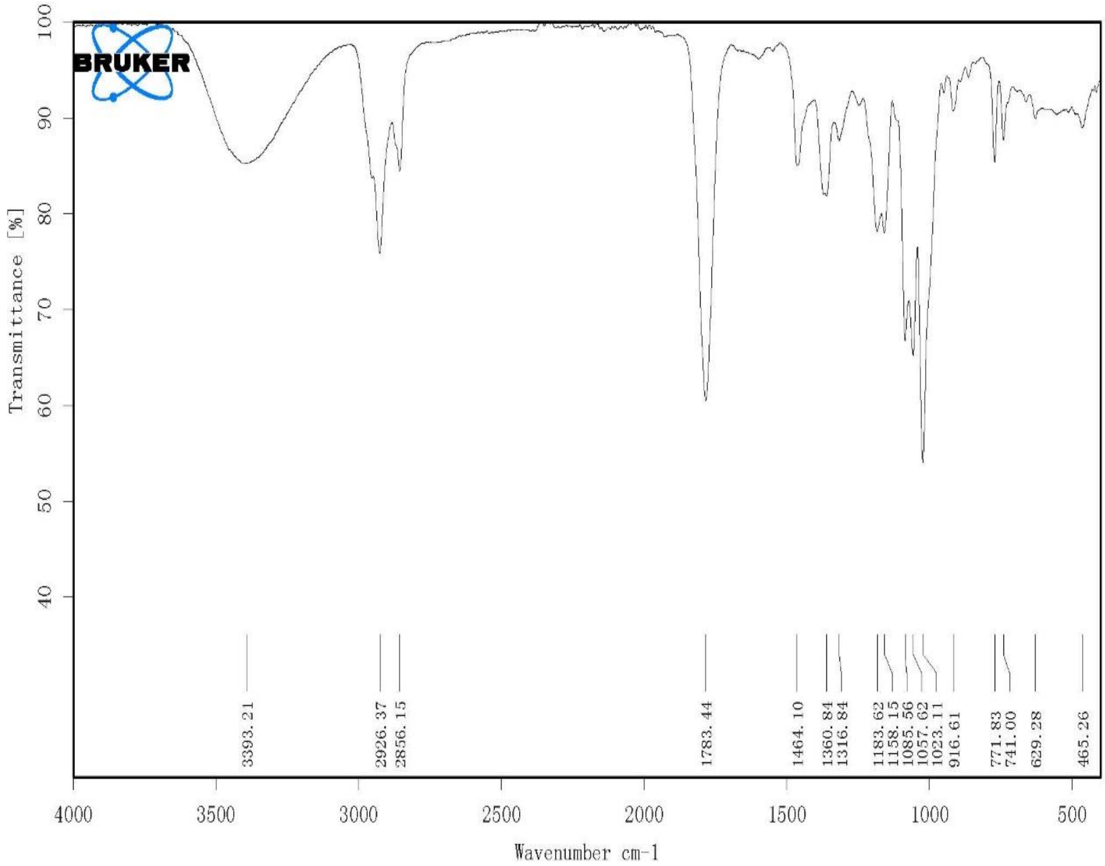

Supplement: Supplementary file 1 [file marinedrugs-21-00382-s001.zip › marinedrugs-2478436-supplementary.pdf]
